# An atlas of evidence-based phenotypic associations across the mouse phenome

**DOI:** 10.1038/s41598-020-60891-w

**Published:** 2020-03-03

**Authors:** Nobuhiko Tanaka, Hiroshi Masuya

**Affiliations:** Integrated Bioresource Information Division, RIKEN BioResource Research Center, 3-1-1 Koyadai, Tsukuba, Ibaraki 305-0071 Japan

**Keywords:** Data integration, Data mining, Data publication and archiving, Functional clustering, Modularity

## Abstract

To date, reliable relationships between mammalian phenotypes, based on diagnostic test measurements, have not been reported on a large scale. The purpose of this study was to present a large mouse phenotype-phenotype relationships dataset as a reference resource, alongside detailed evaluation of the resource. We used bias-minimized comprehensive mouse phenotype data and applied association rule mining to a dataset consisting of only binary (normal and abnormal phenotypes) data to determine relationships among phenotypes. We present 3,686 evidence-based significant associations, comprising 345 phenotypes covering 60 biological systems (functions), and evaluate their characteristics in detail. To evaluate the relationships, we defined a set of phenotype-phenotype association pairs (PPAPs) as a module of phenotypic expression for each of the 345 phenotypes. By analyzing each PPAP, we identified phenotype sub-networks consisting of the largest numbers of phenotypes and distinct biological systems. Furthermore, using hierarchical clustering based on phenotype similarities among the 345 PPAPs, we identified seven community types within a putative phenome-wide association network. Moreover, to promote leverage of these data, we developed and published web-application tools. These mouse phenome-wide phenotype-phenotype association data reveal general principles of relationships among mammalian phenotypes and provide a reference resource for biomedical analyses.

## Introduction

Data analyses that model relationships among omics hierarchies, including genomes, epigenomes, transcriptomes, proteomes, metabolomes, and phenomes, are imperative for holistic understanding of life^1,2^. Elucidation of principles underlying the complex relationships among mouse phenotypes^3–5^ would provide a useful data resource for multi-omics studies in mammalian species and assist in elucidating biological mechanisms underlying pleiotropic gene expression^6^ and multimorbidity^7,8^ (which are understood as secondary phenotype expression^9–11^), as well as human disease etiology. In human, relationships between phenotypes have generally been based on correlations between gene variants and phenotypes, derived from the analyses of genome-wide association studies (GWAS)^12,13^ and phenome-wide association studies (PheWAS)^14–17^. A small number of previous studies have reported phenotype-phenotype relationships by investigating the human phenotype relationships of 24^18^, 42^19^, and 127^20^ traits/diseases, using GWAS/PheWAS summary statistics (LD scores, effect size estimates, and polygenic scores, respectively). Ideally, to derive more reliable data on relationships among phenotypes at the phenome-wide level, study designs including comprehensive phenotype measures and controlling for genetic background and environmental factors, which enable detection of co-expression of abnormal phenotypes in the same individuals, are required; however, in human, it is difficult to meet these conditions due to ethical and experimental constraints. By contrast, comprehensive phenotype analyses of knock-out mouse mutant strains have been performed by the International Mouse Phenotyping Consortium (IMPC)^21^, using standardized genetic backgrounds^22,23^, experimental methods^24–26^, analysis workflows, and control strategies^27^. Enormous amounts of phenotype data, with minimization of the influences of various biases on phenotypic calls (normal/abnormal), are publicly available^28^. The availability of bias-minimized IMPC data to study relationships among phenotypes provides an opportunity to generate a comprehensive and reliable mammalian reference data resource.

The purpose of this study was to provide reliable, weighted phenotype-phenotype relationship data at the mouse phenome-wide level by developing methodologies to identify and evaluate significant phenotype relationships. Using the IMPC data, we first applied association rule mining to derive significant relationships (association rules) among mouse phenotypes. Then, we used a complete set of binaries (normal and abnormal) phenotype data, which included only cases examined for at least two phenotypes, to extract relevant relationships. In addition, we developed an optimal rule selection strategy to extract the maximum amount of reliable relationship data possible. Finally, we obtained 3,686 evidence-based significant association rules, comprising 345 phenotypes involved in 60 biological systems (functions). To clarify the characteristics of these rules, we performed various analyses. For example, to facilitate structural and functional understanding of this resource, we created the concept of a phenotype expression module, referred to as ‘a set of phenotype-phenotype association pairs (PPAPs)’, which includes all phenotypes related to a phenotype of interest. Further, we generated a putative phenome-wide association network, classified into seven sub-networks by hierarchical clustering, based on similarities among phenotypes in each PPAP. We also developed a method to convert PPAPs into pathway-like configurations and, using 283 converted sub-pathways, constructed a putative phenome-wide association pathway. Further, we developed and published web-application tools to promote leverage of these data. This evidence-based mammalian phenome-wide data resource will be useful for elucidation of mechanisms underlying phenotypes and deepening understanding of both pleiotropic gene expression and human disease pathology.

## Results

### Generating high-quality association rules

Using IMPC data (Release 4.3; Methods), after processing (Supplementary Data 1–11; Supplementary Methods), a dataset was prepared for analysis of phenotype relationships (Supplementary Data 12; Supplementary Methods) from comprehensive phenotyping of 3,100 null mutant strains, using 113 standard operating procedures (SOPs), 2,050 measured parameters, and approximately 18,000,000 data points (phenotype measurements); for example, when blood glucose level was measured as 200 mg/dl using a biological chemistry test in an individual mouse, the procedure, the measured parameter, and the data point corresponded to the biological chemistry test, the blood glucose level, and the value ‘200 mg/dl’, respectively. To reduce semantic overlap of phenotypes, we annotated the 2,050 parameters using Mammalian Phenotype (MP)^29^, Mouse Anatomy (MA)^30^, and Edinburgh Mouse Atlas Project (EMAP)^31^ ontologies, by processing the downloaded data containing parameter-ontology relationships. For example, phenotype examinations for the two measured parameters, ‘Fat mass’ and ‘Fat/Body weight’, in the bone density test (DEXA) were summarized as a single phenotype, annotated with the MP ontology term ‘abnormal adipose tissue amount’ (Supplementary Methods). A new criterion, ‘stage_type:ontology_name’, including the stage/type and an ontology term (e.g., ‘adult_trait:abnormal adipose tissue amount’) was defined to distinguish ontology-annotated phenotypes by expression stage (adult/embryo) and type (trait/gene). Mice before and after birth were defined as embryos and adults, respectively, except for mice in which the phenotype ‘embryo_trait:abnormal viability by preweaning’ was analyzed. Using this criterion, we summarized measured parameter level phenotypes into 532 distinct ontology-annotated phenotypes (Supplementary Table 1; Supplementary Methods). For functional (enrichment) analysis of the phenotype dataset, each ontology-annotated phenotype was annotated by its top level term(s), biological system(s) (function); for example, the phenotype ‘adult_trait:abnormal circulating glucose level’ belongs to the biological system ‘homeostasis/metabolism phenotype’.

**Figure 1:**
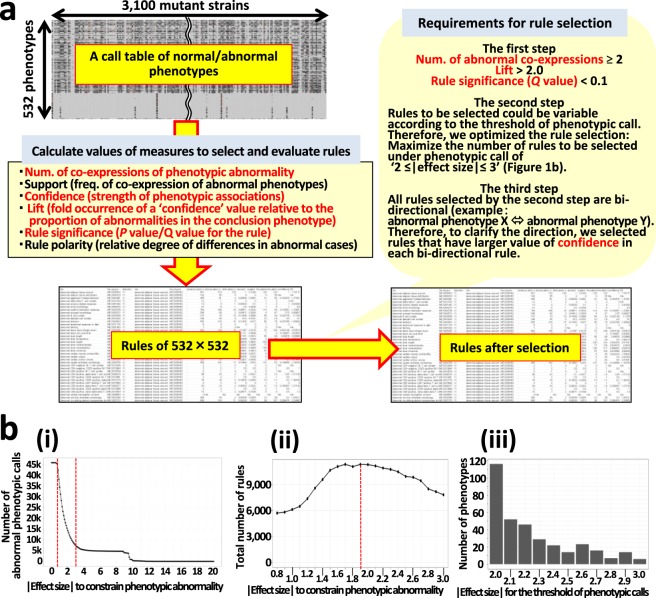
Systematic identification of significant association rules. (**a**) An overview of the workflow for selecting significant phenotypic relationships by association rule mining. Measures highlighted in red font were used for selection. See Methods for detailed descriptions of each measure. (**b**) Optimization of rule selection. (i) Graph showing the number of abnormal cases (y-axis) against variation of the threshold for phenotypic calls with |effect size (ES)| (unbiased Hedge’s g) (x-axis). Red broken line, range of |ES| from 0.8 to 3.0. Note that these abnormal cases were obtained under the conditions where the ES was significant in the 95% confidence interval (*P* value for g < 0.05), and the exact probability obtained by the significance test was < 0.05. (ii) Graph showing the relationships between variation of |ES| in the same range as i (x-axis) and the total number of rules selected under those conditions (y-axis). The number of rules selected under the 23 thresholds of |ES| (bins of 0.1) is presented. Note that these rules were obtained under the conditions in step 2, shown in **a**, and they are all bidirectional. Red dashed line, |ES| (1.9) for the maximum number of rules. The analysis case for each of the 532 phenotypes is available in an interactive application (https://brc-riken.shinyapps.io/effect_size_vs_rules/). (iii) Bar graph showing the relationship between the value of |ES| (the threshold for phenotypic calls) and the number of phenotypes taking its value as optimal. The sum of the number of phenotypes in the y-axis becomes 345.

**Figure 2:**
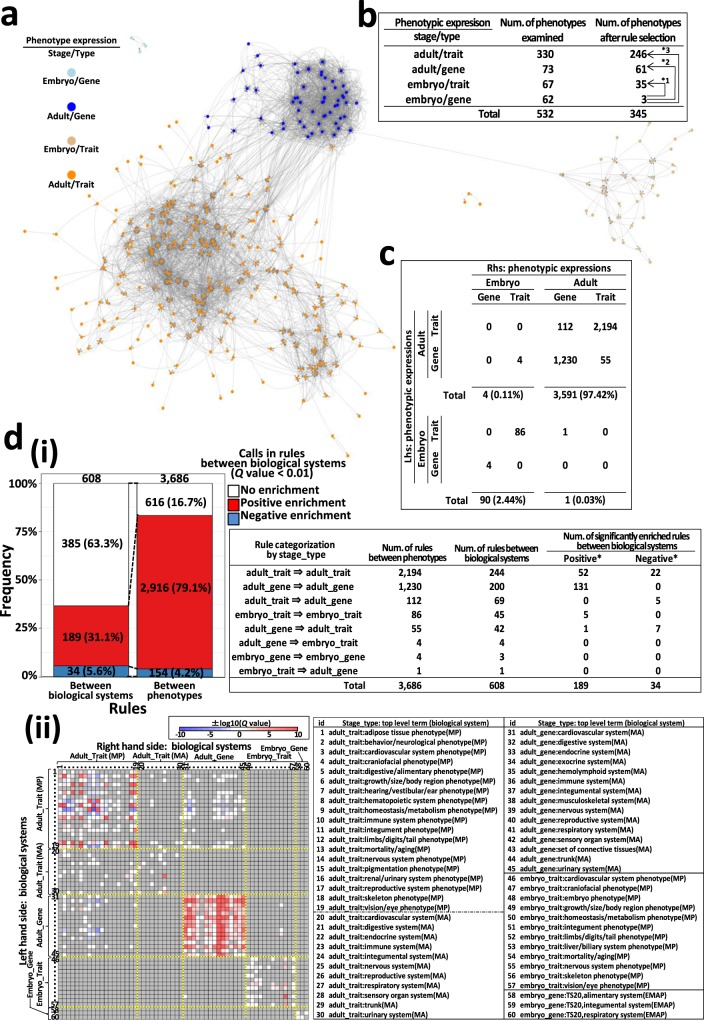
Characterization of 3,686 significant association rules, part 1. (**a**) Visualization. Nodes, abnormal phenotypes (n = 345), classified by ‘stage/type’ category; edges, association rules. (**b**) Phenotypes by ‘stage/type’ category before and after rule selection. *1, *2, and *3, Bonferroni-corrected *P* = 8.8×10^−6^, 1.6×10^−9^, and 1.0×10^−10^, respectively (two-tailed Fisher’s exact test). (**c**) Significant rules by ‘stage/type’. Lhs (left-hand side), premises; Rhs (right-hand side), conclusions of phenotype association rules. (**d**) Enrichment analysis of between-‘biological systems’ rules. (i) Frequency of significantly enriched between-‘biological systems’ rules. Right table, number of significantly enriched between-‘biological systems’ rules by ‘stage/type’ category (two-tailed Fisher’s exact test, *FDR < 0.01). (ii) Heatmap of associations between biological systems. Darker red and blue, greater positive and negative enrichment, respectively. Gray squares, non-existing pairs. Labels 1–60, biological system IDs (see right table). See also Supplementary Table 6.

To extract significant associations among phenotypes, we applied an association rule mining approach^32,33^, where each association of all possible combinations between phenotypes was evaluated strictly by calculating the statistical significance of a confidence value (Methods) for the association rule of ‘Phenotype X => Phenotype Y’ (namely, when Phenotype X is abnormal, the probability that Phenotype Y is abnormal). Based on a normal/abnormal call table, comprising 532 phenotypes × 3,100 mutant genes (mouse strains), we generated a complete set of binary (normal and abnormal) data, which included only cases examined for at least two phenotypes, for each of all possible combinations between phenotypes (532 × 532 = 283,024 patterns), and calculated values for various measures to evaluate those relationships (Fig. 1a; Methods). Analysis of such complete datasets was key for extraction of reliable association rules. For rule selection, we extracted rules fulfilling the following conditions: number of co-expressions between abnormal phenotypes ≥2; lift (probability of rule) > 2; *Q* value (false discovery rate; FDR) for rule significance <0.1 (Fig. 1a). Next, to obtain reliable associations among (abnormal) phenotypes, we adopted an optimal rule selection strategy, which selected the maximum number of association rules possible, under strict phenotypic call thresholds (Fig. 1b). Phenotypic call thresholds were set as effect size (ES) values, maximizing the number of rules selected under the condition ‘2 ≤|ES|≤ 3’, for each phenotype. This optimized condition was generated after determining that numbers of abnormal cases decreased dramatically, from 42,455 to 7,781, in the |ES| range 0.8 to 3.0 (the threshold for phenotypic calls) (Fig. 1b-i); in this |ES| range, the maximum number of total rules selected was at |ES| 1.9 (Fig. 1b-ii), and |ES| values generating the largest number of rules differed for each phenotype (Fig. 1b-iii). We extracted and accumulated rules under optimal conditions for each phenotype and removed duplicates. As all rules extracted were bidirectional, we only selected those with higher confidence values for the individual unidirectional rules, to enable relative comparisons of numbers of abnormal cases between phenotype pairs (Fig. 1a). Finally, we identified 3,686 association rules (1.4% of all 265,036 possible pairwise combinations) (Fig. 2a and Supplementary Table 2), comprising 345 phenotypes, covering 60 biological systems (functions) (Supplementary Table 3).

### Characterization of the 3,686 significant association rules

The 3,686 association rules were extracted using data obtained under the ideal experimental design (as far as possible) and by applying the optimal rule selection strategy. Before these rules could be promoted for use as a mammalian phenotype relationship reference resource, it was essential to clarify their characteristics; therefore, we analyzed the rule characteristics in detail.

Following rule selection, the 532 phenotypes examined decreased to 345 (35.2% reduction, Fig. 2b) and we examined the functional composition of these 345 phenotypes constituting the rules (Fig. 2b and Supplementary Table 4). The reduction rate of phenotypes classified as ‘stage/type’ ‘embryo/gene’ was remarkably high (95.2%, from 62 to 3; *P *= 3.9 × 10^−9^, two-tailed Fisher’s exact test), and differed significantly from that of the other three categories (Bonferroni-corrected *P* = 1.0 × 10^−10^ vs. ‘adult/trait’, 1.6 × 10^−9^ vs. ‘adult/gene’, and 8.8 × 10^−6^ vs. ‘embryo/trait’; two-tailed Fisher’s exact test, Fig. 2b). This specific reduction is because ‘embryo/gene’ phenotypes exhibited a very high proportion of abnormality (approximately 65% in the |ES| range 0.8–3.0), inevitably generating more rules not fulfilling the selection criteria. To examine features associated with decreased numbers of phenotypes in specific biological systems following rule selection, we compared the rates of decrease in phenotype number following rule selection for 82 biological systems covering the 532 phenotypes. No significant differences were observed, indicating no specificity in the rate of phenotype number decrease across the 82 biological systems following rule selection (Supplementary Table 4).

To understand the landscape of phenotype-phenotype relationships constituting the 3,686 association rules, we first classified phenotypes into four ‘stage/type’ categories, and examined the number of rules for each of the 16 possible pairwise category combinations (Fig. 2c). ‘Between-phenotypes’ rules, expressed at adult stages, accounted for 97.4% of the total (3,591/3,686). Furthermore, ‘between-phenotypes’ rules, expressed at the same stage (adult or embryo), accounted for 99.8% of the total (Fig. 2c). We performed enrichment analysis to evaluate the specificity of numbers of rules in each of the 16 categories (Supplementary Table 5). We found significant positive enrichment for the three relationships between the same categories (‘adult_gene => adult_gene’, *P* = 0; ‘adult_trait => adult_trait’, *P* = 1.5 × 10^−40^; ‘embryo_trait => embryo_trait’, *P* = 0.004; Holm’s-corrected *P*), demonstrating positive extraction of those three relationships by rule selection. The remaining 13 ‘stage/type’ rule categories exhibited significant negative enrichment (Holm’s-corrected *P*, 1.0 × 10^−126^ to 3.7 × 10^−17^; Supplementary Table 5), indicating that they were negatively extracted by rule selection. Additionally, we examined the 3,686 rules by between-‘biological systems’ rule category, and identified 608 (8.7%) between-‘biological systems’ rules (among 6,973 possible distinct such rules; Fig. 2d-i and Supplementary Table 6). This number (608) was significantly less than the expected value (2,041) (bootstrap *P* value = 0), representing extreme selectivity of the 608 between-‘biological systems’ rules. Enrichment analysis for the 608 between-‘biological systems’ rules identified 189 positively enriched (31.1%) and 34 negatively enriched (5.6%) relationships (FDR < 0.01; Fig. 2d-i and Supplementary Table 6). We developed a tool to visualize these relationships, according to user-selected biological system (Supplementary Fig. 1), to facilitate understanding of the rules and their degree of enrichment, and promote use of the dataset (Fig. 2d-ii and Supplementary Table 6).

**Figure 3:**
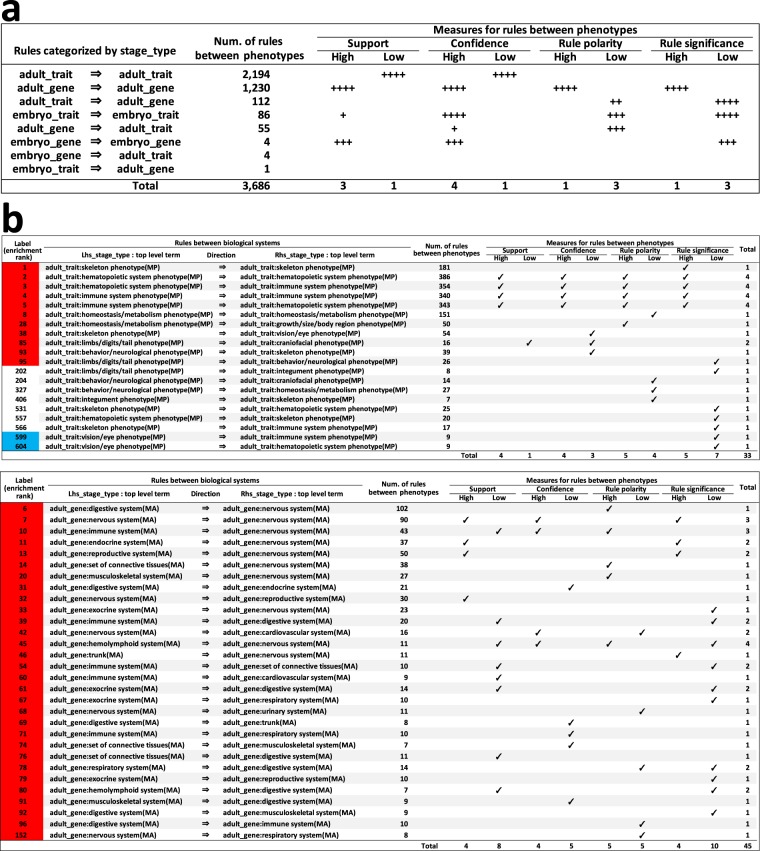
Characterization of 3,686 significant association rules, part 2. Characteristics of significant association rules as values for support, confidence, rule polarity, and rule significance. (**a**) Enrichment for larger and smaller values of the four measures by between-‘stage/type’ rules. +, uncorrected *P* < 0.05; ++, +++, and ++++, Bonferroni-corrected *P* <0.05, 0.01, and 0.001, respectively. **(b**) Between-‘biological systems’ rules with outstanding mean values for the four measures. Tables above and below, summary results for between-‘adult/trait’ and between-‘adult/gene’ rules, respectively. Red (blue) in ‘Label’ indicates that between-‘biological systems’ rules were significantly positively (negatively) enriched among the selected 3,686 rules. Between-‘biological systems’ rules are arranged in descending order of enrichment degree. See also Supplementary Data 13.

To understand the characteristics of the 3,686 selected association rules, we analyzed values of four representative measures (support, confidence, rule polarity, rule significance) for each of the 3,686 association rules, in detail (Supplementary Data 13, Supplementary Fig. 2 and Supplementary Tables 7–13). For example, we conducted enrichment analysis to determine which between-‘stage/type’ rules were concentrated among the upper/lower values of these four measures, demonstrating that ‘adult_trait => adult_trait’ between-‘stage/type’ rules were significantly enriched among lower values of support and confidence, whereas ‘adult_gene => adult_gene’ rules were significantly enriched among higher values of all four measures (Supplementary Table 7 and Fig. 3a). We also conducted a similar enrichment analysis for between-‘biological systems’ rules; the resulting distinctive features are summarized in Fig. 3b. Twenty distinctive between-‘biological systems’ rules were identified among between-‘adult/trait’ rules (Fig. 3b, above), with 30 among between-‘adult/gene’ rules (Fig. 3b, below). Refer to Supplementary Data 13 for a detailed explanation of the results of analysis for these four measures.

### Phenotype-phenotype association pair (PPAP) sets

**Figure 4:**
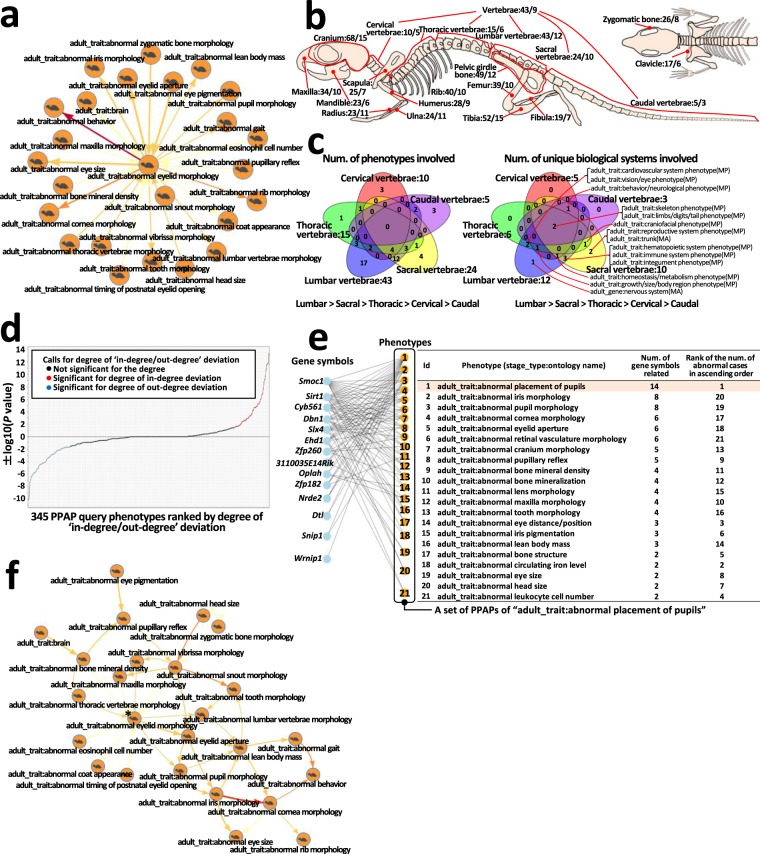
Analysis of PPAPs. (**a**) PPAP with query phenotype ‘adult_trait:abnormal eyelid morphology’ (center) and related phenotypes (nodes at the periphery) via edges (arrows; darker color, larger rule polarity value; wider line, larger confidence value). (**b**,**c**) Example of relationships among 345 PPAPs. Relatedness of anomalies in skeletal parts with other phenotypes was examined. **(b)** Whole skeleton. Two numbers at the end of the name of each part of the skeleton indicate ‘num. of PPAP-constituting phenotypes” (first number) and ‘num. of PPAP-constituting distinct biological systems’ (second number). (**c**) Five vertebrae regions. (**d**) Characterization of PPAPs by ‘indegree/outdegree’ deviation (y-axis; -log10 (*P* value)) for ‘indegree/degree’ vs. ‘outdegree/degree’ (two-tailed Fisher’s exact test)) for each query phenotype. Negative if (outdegree) – (indegree)>0. Positive if (indegree) – (outdegree) ≥ 0. PPAPs (x-axis) are arranged in ascending order of signed log value. Of phenotypes showing significant deviation (FDR <0.05), 60 negatives (blue) were considered relatively rare, and 49 positives (red) were considered relatively common. (**e**) PPAP-driven gene-phenotype association for the PPAP with query phenotype ‘adult_trait:abnormal placement of pupils’. (**f**) Conversion from PPAP, shown in a, to pathway-like configuration. Asterisk, query phenotype. See also Methods and Supplementary Fig. 3.

We defined all association rules between phenotypes of interest and their directly/indirectly related phenotypes as PPAP sets, and assigned this as an analysis unit, or a phenotypic expression module (Fig. 4a). As an example of PPAP analysis, we focused on PPAPs with query phenotypes relating to skeletal system parts. Comparative analysis of the PPAP-constituting phenotypes and biological systems (functions) demonstrated a general principle regarding the range of relationships among skeletal parts (Fig. 4b). In addition, comparative analysis among PPAPs, focusing on five regions of the vertebral column, revealed that the numbers of PPAP-constituting phenotypes and their distinct biological systems decreased in the following order: ‘Lumbar > Sacral > Thoracic > Cervical > Caudal’, and identified region-specific phenotypes and biological systems (Fig. 4c). We next conducted comparative analyses of PPAP-constituting phenotypes according to ‘stage/type’ and ‘biological system’ query phenotype categories, and summarized the results in Supplementary Data 14 and Supplementary Tables 14–17.

### Detection of sub-networks consisting of the largest numbers of phenotypes and distinct biological systems

The phenotypes constituting each PPAP were presumed to belong to the same phenotype expression association networks and be components of sub-networks in a putative phenome-wide association network. Therefore, larger numbers of phenotypes constituting a PPAP represent sub-networks, consisting of more abnormal phenotypes, and larger numbers of distinct biological systems among PPAP-constituting phenotypes represent sub-networks covering wider ranges of biological systems. For each of the 345 PPAPs, we examined the number of constituting phenotypes, distinct biological systems, and phenotypes belonging to the same biological system as the query phenotype (Supplementary Tables 18–20, respectively).

Among all 345 PPAPs, the PPAP comprising the largest number of phenotypes had the query phenotype ‘adult_trait:abnormal lean body mass’ (biological system, ‘adult_trait:growth/size/body region phenotype(MP)’) (Supplementary Table 18), and comprised 82 abnormal phenotypes, all of the ‘adult/trait’ category, corresponding to 33.3% of all 246 ‘adult/trait’ phenotypes. This PPAP comprised phenotypes covering 17 distinct biological systems; the largest number of distinct ‘adult/trait’ phenotype biological systems (Supplementary Table 14). Among PPAPs with ‘adult/gene’ query phenotypes, the PPAP with the query ‘adult_gene:aorta’ (biological system, ‘adult_gene:cardiovascular system(MA)’) comprised the largest number of both phenotypes (65) and distinct biological systems (22) (Supplementary Tables 18 and 19, respectively). Of phenotypes within PPAPs from the same biological system as the query phenotype, a PPAP with the query ‘adult_trait:abnormal CD4-positive, alpha beta T cell number’ (biological systems: ‘adult_trait:hematopoietic system phenotype(MP)’ and ‘adult_trait:immune system phenotype(MP)’) contained the largest number (29) of abnormal phenotypes (Supplementary Table 20). Among all 345 PPAPs, that comprising the largest number of distinct biological systems was for the query ‘adult_trait:abnormal mean corpuscular volume’ (biological system, ‘adult_trait:hematopoietic system phenotype(MP)’) (Supplementary Table 19), including 24 biological systems, comprising 77 abnormal phenotypes, and 11 ‘adult/gene’ biological systems, corresponding to 29 abnormal phenotypes (Supplementary Table 14).

### Identifying phenotypes with relatively more/less abnormal cases

The numbers of incoming/outgoing phenotypes relative to the query phenotypes in each PPAP indicate phenotypes with fewer/more abnormal cases, compared with the number of abnormal query phenotype cases, respectively. Therefore, the degree of deviation between the numbers of incoming and outgoing phenotypes was used to measure relative rank (position) for the number of abnormal query phenotype cases in each PPAP (Methods). Using this measure, we analyzed the relative frequency of abnormal cases for each query phenotype for all 345 PPAPs (Fig. 4d and Supplementary Table 3), and identified 60 and 49 less and more frequently occurring phenotypes (two-tailed Fisher’s exact test, FDR < 0.05; Supplementary Tables 21 and 22, respectively). Enrichment analysis for less and more frequently occurring phenotypes, according to the four ‘stage/type’ phenotype categories, demonstrated statistically significant enrichment of ‘adult_gene’ phenotypes for both groups at α = 0.05 (Supplementary Table 23; one-tailed Fisher’s exact test). Also, on classification of phenotypes by biological systems, ‘adult_gene:nervous system’ phenotypes were significantly enriched among ‘more frequently occurring phenotypes’ (Bonferroni-corrected *P* = 0.0068; Supplementary Table 23).

### Relationships among phenotypes in each PPAP

In considering the collection of phenotypic abnormalities within each PPAP as the symptoms of a human disease, presentation of ‘the order (flow), according to the relative level of abnormal case numbers in each phenotype’ among phenotypes in the collection could facilitate understanding of principles underlying gene pleiotropy^34^, secondary phenotypes^9–11^, and comorbidity^7,8^. Therefore, we ranked PPAP-constituting phenotypes linearly, based on rule polarity values for each rule within each PPAP (logarithmic *P* value of the test for differences in the number of abnormal cases between the two phenotypes in each rule; the degree of deviation of the difference) (Fig. 4e; Methods; data available through the web application, Supplementary Fig. 3). Furthermore, to present possible routes from phenotypes with relatively few abnormal cases to those with relatively many abnormal cases, for ‘between-phenotypes’ rules constituting each PPAP, using data from the significant association rules between PPAP-constituting phenotypes, we converted PPAPs to pathway-like configurations, using a strategy that took the longest path from the selected phenotype to each related phenotype (Fig. 4f; Methods). For 283 convertible PPAPs, among the total of 345 (Methods), we both visualized converted pathway-like configurations and added data regarding these relationships to the web application (Supplementary Fig. 3).

### Construction and functional mapping of a putative phenome-wide phenotype-phenotype association pathway

**Figure 5:**
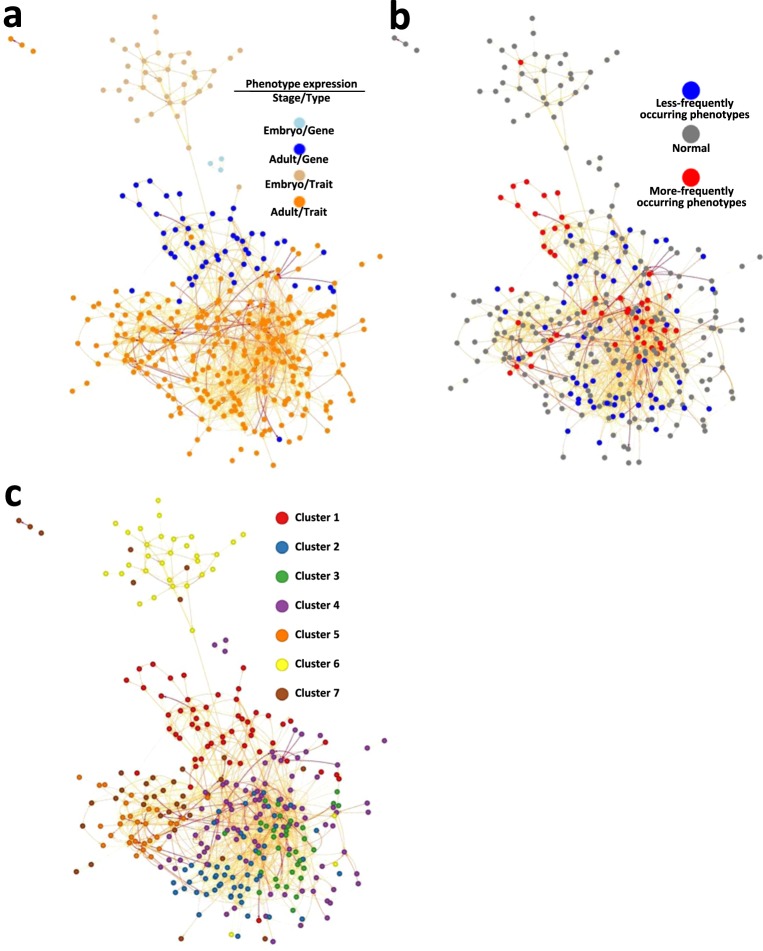
Landscape of a putative phenotype-phenotype association pathway across the mouse phenome. The overall picture is composed of 1,543 association rules and 335 nodes (phenotypes) (Supplementary Table 24) and distinguished by four ‘stage/type’ categories (**a**), three types of abnormal occurrence frequencies **(b)**, and the seven sub-clusters (described in Fig. 6) (**c**). A darker edge color indicates a larger rule polarity value.

**Figure 6:**
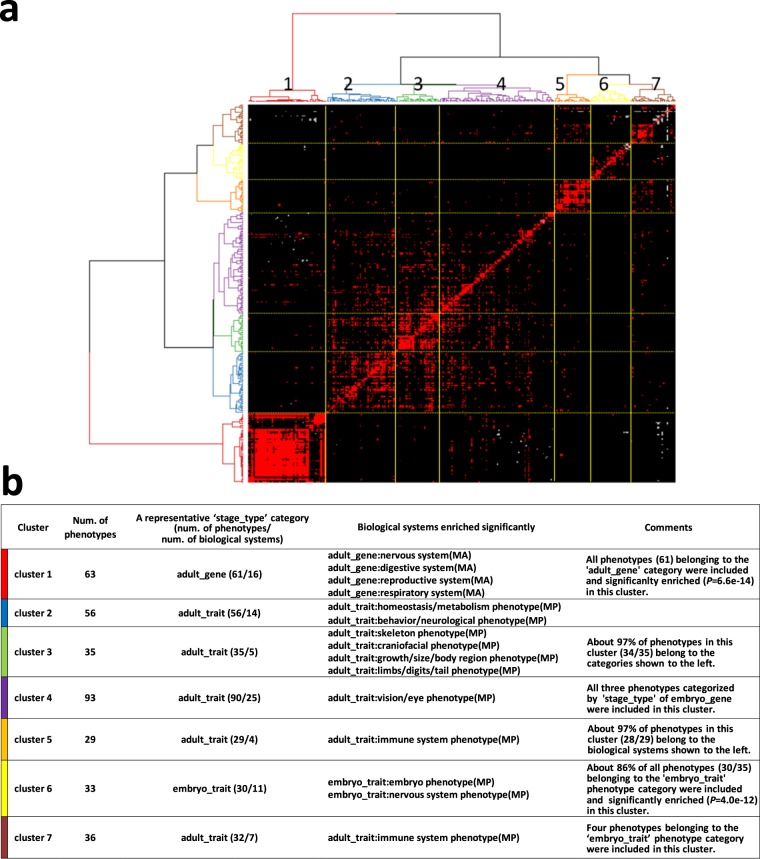
Overall picture of the relationships among the 345 PPAPs. (**a**) Clustered heatmap of components (phenotypes) constituting each of the 345 PPAPs. The longitudinal and horizontal axes represent the 345 PPAPs and their 345 query phenotypes, respectively. The order of the query phenotypes is shown in Supplementary Table 2. Red, positive for phenotypic abnormality; black, negative for phenotypic abnormality; Gray, no test was performed in the corresponding pairs of phenotypes. Hierarchical clustering was conducted using Simpson distance and Ward linkage. Note that this heatmap is symmetrical. (**b**) The characteristics of the seven sub-clusters generated by hierarchical clustering (see Supplementary Data 15 for details).

To comprehensively model the relationships among abnormal phenotypes, we constructed a putative phenome-wide phenotype-phenotype association pathway, comprising 335 nodes (phenotypes) and 1,543 edges (association rules), by accumulating all association rules constituting the 283 converted pathways and eliminating duplicates (Fig. 5 and Supplementary Table 24). Routes could occur that have not been confirmed experimentally for the whole expression pathway; however, all rules between two adjacent phenotypes constituting the pathway were experimentally confirmed and statistically significant. These predictive routes through the whole pathway represent a resource for identification of predicted relationships between phenotypes not included in a PPAP. We also mapped phenotypes according to ‘stage/type’ categories (Fig. 5a), less frequently/more frequently occurring categories, shown in Fig. 4d (Fig. 5b), and the seven subgroups, shown in Fig. 6 (Fig. 5c), onto the pathway.

### Detecting communities in the putative phenome-wide phenotype-phenotype association network

More shared phenotypes between PPAPs indicate higher path sharing between them in the putative phenome-wide phenotype-phenotype association network. To detect communities within the putative phenome-wide network, we performed hierarchical clustering, based on similarities between phenotypes in each of the 345 PPAPs (Fig. 6), which revealed that the putative association network could be classified into seven sub-clusters (Fig. 6a and Supplementary Table 3). The functional features of each cluster were examined in detail (Supplementary Data 15) and are summarized in Fig. 6b.

Briefly, in cluster 1, 61 of 63 phenotypes (97%) belonged to the ‘stage/type’ ‘adult/gene’, and ‘adult/gene’ phenotypes were significantly enriched in this cluster (two-tailed Fisher’s exact test, *P* = 6.6 × 10^−14^). Enrichment analysis of phenotypes in this cluster, according to their biological systems, revealed statistically significant enrichment of phenotypes belonging to the biological systems ‘adult_gene:nervous system (MA)’, ‘adult_gene:digestive system (MA)’, ‘adult_gene:reproductive system (MA)’, and ‘adult_gene:respiratory system (MA)’. In cluster 6, 30 of 35 phenotypes (86%) belonged to the ‘embryo/trait’ category, and ‘embryo/trait’ phenotypes were significantly enriched in this cluster (two-tailed Fisher’s exact test, *P* = 4.0 × 10^−12^). In addition, phenotypes belonging to the biological systems ‘embryo_trait:embryo phenotype (MP)’ and ‘embryo_trait:nervous system phenotype (MP)’ were significantly enriched in this cluster. Clusters 2–4 each comprised a single least common cluster, each with specific features, significantly enriched as follows: ‘adult_trait:homeostasis/metabolism phenotype(MP)’ and ‘adult_trait:behavior/neurological phenotype(MP)’ phenotypes in cluster 2; ‘adult_trait:skeleton phenotype(MP)’, ‘adult_trait:craniofacial phenotype(MP)’, ‘adult_trait:growth/size/body region phenotype(MP)’, and ‘adult_trait:limbs/digits/tail phenotype(MP)’ phenotypes in cluster 3, related to external/internal morphology; and ‘adult_trait:vision/eye phenotype(MP)’ phenotypes in cluster 4. Both clusters 5 and 7 were enriched for biological system ‘adult_trait:immune system phenotype(MP)’ phenotypes. Phenotypes involving T cells, part of the acquired immune system, were enriched in cluster 5. In cluster 7, phenotypes involving B cells, which also contribute to the acquired immune system, and innate immune system phenotypes, such as abnormal monocyte, abnormal dendritic, and abnormal neutrophil cell numbers, were enriched (Supplementary Data 15 and Supplementary Table 3). These results indicate that ‘adult_trait:immune system phenotype(MP)’ biological system phenotypes are classified into at least two sub-networks in the putative phenome-wide phenotype-phenotype network, based on differences in biological functions.

**Figure 7:**
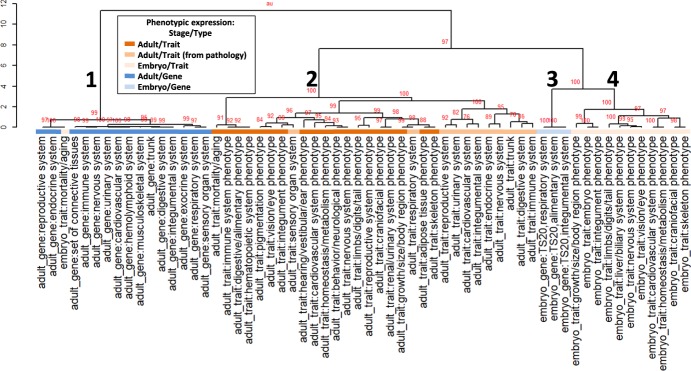
A dendrogram showing the overall relationships among 60 biological systems. The dendrogram was constructed by hierarchical clustering, based on the similarity of biological systems to phenotypes constituting each of the 345 PPAPs. Hierarchical clustering was conducted using Simpson distance and Ward linkage. Red values on the edges of the dendrogram are AU (Approximately Unbiased) *P* values (%). The AU *P* values were calculated by applying the *cd*.*pvclust* function of the R source *pvclust-cd*, with 1,000 bootstrap replications. Biological systems on the dendrogram are colored according to their ‘stage/type’ categorization for phenotypic expression. Refer to Supplementary Fig. 4 for details.

We generated an overview of the relationships among the 60 biological systems by hierarchical clustering, based on the similarity of distinct biological systems in each of the 345 PPAPs (Fig. 7, Supplementary Fig. 4, and Supplementary Table 25). The 60 biological systems were clearly classified into four clusters, corresponding to the four ‘stage/type’ phenotype categories (Fig. 7 and Supplementary Fig. 4), demonstrating selective connections between biological systems in the same ‘stage/type’ category (right table, Fig. 2d-i). Together with the data on pairwise relationships between biological systems (Fig. 2d-ii and Supplementary Fig. 1), this overview of the relationships among biological systems provides a reference resource to facilitate understanding of relationships among biological systems associated with phenotypic abnormality.

### Deriving phenotype-driven modular pleiotropy

The set of abnormal phenotypic expressions constituting each PPAP is presumed to share common genetic factors with human diseases exhibiting symptoms comparable to those phenotypic abnormalities. To understand the genetic factors (background) of each PPAP, we made lists ranking the 3,100 gene-knockout strains available through the web application, where mutant strains with greater numbers of shared abnormal phenotypes, compared with each set of PPAP-constituting phenotypes, are listed in descending order of the number of shared phenotypes (Supplementary Fig. 3 and Fig. 4e; Methods). The resulting 345 datasets of phenotype-driven gene-phenotype relationships, available through the web application, represent genuine modular pleiotropy^6,35–38^, based on empirical evidence.

## Discussion

In this study, we present overviews of the relationships among 345 measured parameter-based phenotypes and connections between their 60 biological systems, based on comprehensive mouse phenotype data analyses, alongside detailed functional and structural analyses of these two landscapes. The relationships between phenotypes in human GWAS/PheWAS are generally obtained indirectly, based on genetic correlations among gene-phenotype relationships^18–20^. By contrast, the mouse phenotype-phenotype relationships presented here are evidence-based phenotype relationships, which were obtained based on abnormal phenotype co-expression, determined by direct measurement of multiple phenotypes per individual. In addition, each relationship (association rule) was given a weight and direction by calculating the values of four measures (support, confidence, rule polarity, and rule significance), allowing deeper insights into each relationship. To our knowledge, this is the most comprehensive and reliable mammalian phenotype relationship data resource, based on empirical evidence, and can be leveraged as a reference resource for various biomedical analyses.

A PPAP represents the maximum possible number of phenotypic abnormalities that can potentially occur alongside a selected abnormal phenotype; therefore, for initial phenotypic analyses of gene mutant strains, screening of these relationships during experimental design could contribute to efficient detection of phenotypic abnormalities^33,39^. Additionally, our data represent a starting point for detailed analyses of the mechanisms underlying the individual identified phenotype associations by interested researchers. For example, we detected phenotypic associations with low abnormality co-occurrence frequencies (i.e., the support value for each association rule was low) as statistically significant rules, providing an opportunity for detailed analyses of rare associations that cannot be detected by small-scale analysis, such as the relationship between eyes and teeth (Fig. 4e), which are both ectoderm-derived. Further, our data represent a potential mammalian phenotype-phenotype associations reference resource for ongoing multi-omics (trans-omics) studies. As the phenome is the bottommost layer of various omics hierarchies, corresponding to all symptoms of human disease, application of our data to trans-omics studies will facilitate understanding of human disease etiology. Our dataset of phenotypic associations, with numerous potential applications, is available online (phenotypes associations, https://brc-riken.shinyapps.io/phenotypic_associations_across_the_mouse_phenome/; biological systems associations, https://brc-riken.shinyapps.io/associations_between_biological_systems/).

Several points should be considered when using our dataset of mammalian phenotype-phenotype associations as a reference resource in future studies. First, each association rule presented here indicates the direction (flow) from the relatively smaller number of abnormal cases to the relatively larger number of abnormal cases, not the causality. In addition, the two phenotypes in each association rule are a mixture of those that are directly or indirectly related to each other, and the existence of confounding factors cannot be excluded. Second, the phenotype data used in this study is derived from the 3,100 IMPC gene knock-out mouse lines, which covers approximately 15.5% of the roughly 20,000 genes that constitute the mouse genome. In the future, by incorporating the updated IMPC phenotype dataset, we may be able to discern novel relationships not present among the association rules presented in this study. Third, the between-phenotype association rules obtained here were derived by considering all examined phenotypes simultaneously; therefore, there is a possibility that cases with positive relationships could be missed by the restrictions on the order of phenotyping tests. To solve this problem, it would be useful to acquire phenotype data in time series and handle phenotypes individually, although this approach would be very time-/cost-/labor-consuming. Fourth, the phenotypes used here were organized into 532 ontology-annotated phenotypes to facilitate normal/abnormal discrimination. For phenotypes derived from quantitative data, by annotating them with ontology terms that can discriminate the direction (positive or negative) of phenotype abnormalities, it is expected that the relationships between phenotypes can be obtained at higher resolution.

Besides relationships between phenotypes, we determined phenotype-driven gene-phenotype relationships, representing associations among abnormal phenotypes constituting 345 PPAPs and their corresponding potential risk genes (Fig. 4e), allowing successful presentation of modular pleiotropy at the phenome-wide level. Associating phenotypes constituting each PPAP with symptoms of specific human diseases (e.g., using the OWLSim algorithm; http://owlsim.org)^32,33,40^ for these gene-phenotype relationships enables identification of candidate risk genes for human diseases. This study also describes a novel methodology for analysis and integration of comprehensive phenotype data. This analysis workflow can be applied to derive significant associations from measured data derived from various biological layers.

## Methods

### Dataset preparation for analysis of phenotypic associations

A dataset (Release 4.3) from the FTP site (ftp://ftp.ebi.ac.uk/pub/databases/impc/) of the IMPC was acquired for analysis. All data processing and analysis in this study was carried out using R programming language^41^. Details of the dataset preparation for analysis of phenotypic associations are described in Supplementary Methods.

### Setting phenotypic call thresholds

In response to the report of Cumming (2014)^42^ and the guideline of the American Statistical Association (2016)^43^, we adopted ES (unbiased Hedges’ g) as the main measure of phenotypic calls, as it is a quantitative measure of the magnitude of the phenotypic difference between the mutation group and the control group, rather than the *P* value of the statistical significance test. When making phenotypic calls using ES, the prerequisites were as follows: the ES was significant in the 95% confidence interval (*P* value for g < 0.05), and the exact probability obtained by the significance test was <0.05. Cohen’s benchmark (1988)^44^ of ‘large effect at 0.8’ is generally used as the threshold for ES for phenotypic calls; however, in this study, to take a more empirical approach, optimal thresholds were examined for each phenotype under the above-mentioned prerequisites, based on the maximum number of significant associations between phenotypes when changing the magnitude of the |ES| from 0.8 to 3.0, in bins of 0.1. Finally, 3,686 significant rules were extracted by filtering the accumulated rules acquired under different optimal conditions for each phenotype (see ‘Generating high-quality association rules’ in the main text for details).

### Association rule mining for 532 phenotypes

To identify significant associations between phenotypes, we applied association rule mining, which is well-known as market basket analysis in the marketing field. Association rules between phenotypes were determined using the ‘if (lhs: left-hand side)-then (rhs: right-hand side)’ style, such as ‘phenotype X => phenotype Y’. Based on the normal/abnormal phenotypic call matrix of 532 phenotypes × 3,100 gene symbols (mutant strains), values for a number of measures were calculated for all pairwise combinations between the 532 phenotypes, to allow evaluation of association rules from various perspectives (refer to the next section). Note that, when calculating each of the measures described in the next section, a pairwise dataset, consisting of only normal and abnormal calls, was used. Hence, in many cases, to investigate associations between two phenotypes within a pairwise dataset, all cases with no test in at least one phenotype were removed for the calculation of values of these measures.

### Measures indicating various features of association rules

Details of representative measures calculated during association rule mining between phenotypes in this study are described below.

#### Num. of abnormal co-expressions

In this study, values of ≥2 for this measure were adopted as requirements for significant rule selection. This threshold was set based on the consideration that we expected to select phenotypic associations, even for phenotypic abnormalities that rarely occur, and that the frequency of phenotypic abnormality was actually only approximately 4% at the ES threshold |2.0|; hence the probability of two cases of abnormal phenotype duplication under this condition is theoretically only 0.026%.

#### Support

Co-expression frequency in two abnormal phenotypes. In this study, we did not adopt this measure for rule selection because, even if a rule shows low frequency by this measure, it can hopefully still be selected by statistical significance.

#### Confidence

When phenotype X is abnormal, the proportion of phenotype Y that is abnormal (conditional abnormal probability). This measure is an indication of how often the rule ‘phenotype X => phenotype Y’ has been found to be true. That is, this measure represents rule accuracy. In this study, because we intended to extract statistically significant rules even if their confidence values were low, we adopted this measure only at the third step of rule selection (in the selection of a single rule with a higher value of this measure from bidirectional rules between two phenotypes).

#### Lift

A measure indicating likelihood in confidence value. This measure indicates, when a rule of ‘phenotype X => phenotype Y’ is raised, how large a confidence value is compared with the proportion of abnormalities of phenotype Y alone. In this study, lift>2 was adopted as a rule selection criterion (lift = 1 represents independence). Note that the two values of this measure in a bidirectional rule take the same value.

#### Rule polarity

A measure representing the magnitude of the difference between the two confidence values in a bidirectional rule (that is, -log10(*P* value) from two-tailed Fisher’s exact test). Considering the formula used to calculate the confidence value, the larger the value of this measure, the larger the difference between the number of abnormal cases in the premise and those in the conclusion of an association rule. Therefore, this measure indicates the relative degree of difference between the number of abnormal cases in the premise and those in the conclusion of an association rule. In this study, because an association rule that had a larger confidence value in a bidirectional rule was selected as one of the 3,686 significant rules, a larger value of this measure represents a relatively smaller number of abnormal cases in the premise.

#### Rule significance

A measure to judge whether or not a rule is statistically significant. In practice, this measure indicates the statistical significance of the lift value (that is, −log10(*P* value) from two-tailed Fisher’s exact test). In this study, *P* values for this measure were calculated only in cases of ‘num. of abnormal co-expressions ≥ 2’. Using these *P* values, *Q* values for multiple testing correction were calculated. The threshold FDR (*Q* value) < 0.1 was adopted as a rule selection criterion. Note that the two values of this measure in a bidirectional rule take the same value, as for the lift.

Of the six measures described above, four (num. of abnormal co-expressions, confidence, lift, and rule significance) were applied for rule selection (Fig. 1). For measuring statistical dependence between the four measures, Spearman’s correlation coefficients and their associated *P* values were calculated using the R package *PerformanceAnalytics* (Supplementary Fig. 2e).

### Calculation of Q values for FDR control

We calculated *Q* values for multiple comparison correction using a sliding linear model^45^ and the *adjust*.*p* function of the R package *cp4p*, with pi0.method = “slim” and alpha = 0.05. For *Q* value calculation, *P* values for the measure rule significance (Fig. 1a), *P* values calculated to examine the degree of enrichment for 608 associations between biological systems (right table, Fig. 2d-i), and *P* values calculated to identify less frequently/more frequently occurring phenotypes by analysis of the 345 association rule sets (Fig. 4d) were applied. For multiple comparison correction of other analyses, Bonferroni or Holm correction of *P* values was applied.

### Enrichment analysis

Many enrichment analyses were conducted for the 3,685 significant rules, to extract features of selected subgroups. Where we only wished to detect significant positive enrichment, *P* values were calculated only for fold-enrichment ≥1 (one-tailed Fisher’s exact test). Where we wished to detect both significant positive and negative enrichments, *P* values were calculated for all pairwise combinations (two-tailed Fisher’s exact test).

### Multiple comparison analysis

Multiple comparisons analyses were conducted for the four interest measures in the 3,686 significant rules by both stage/type and biological system rule classification (Supplementary Data 13 and Fig. 3), and for the numbers of both phenotypes and distinct biological systems in each PPAP by stage/type and biological system classifications of query phenotypes in 345 PPAPs (Supplementary Data 14). A conventional sequence of procedures in these analyses is described below. First, using the R package *userfriendlyscience*, one-way ANOVA was used to determine whether there was a significant difference among categories, where each category had a sample size of ≥7. If a significant difference was detected by ANOVA (*P* < 0.05), the homogeneity of the variances was tested by Levene’s test using the R package *car*. When equal variance was not assumed by the test (*P* < 0.05), *P* values for all pairwise combinations among the groups to be compared were calculated using a Games-Howell post-hoc test in the R package *userfriendlyscience*. The resulting *P* values were then transformed into logarithmic (base-10) scale, and the transformed values (−log10(*P* value)) were used for hierarchical clustering (Euclidean distance and Ward’s linkage) to discover outstanding features for comparisons between biological systems.

### Converting a PPAP into a pathway-like configuration

To understand continuous relationships among phenotypes in each of the 345 PPAPs, we developed a methodology to convert PPAPs to pathway-like configurations, using the 3,686 significant rules (Fig. 4f). The conversion procedure was as follows: **(1)** Focus on outgoing phenotypes of the query phenotype in a PPAP. Select one outgoing phenotype, search all possible paths from the query phenotype to the selected one, and extract the longest (i.e., the path with the most associations with other phenotypes). **(2)** By extracting the longest paths for the remaining outgoing phenotypes by a similar process to that described in (1), and by accumulating all extracted paths, obtain a dataset consisting of all possible paths from the query phenotype to the outgoing phenotypes. **(3)** For incoming phenotypes of the query phenotype, obtain a dataset consisting of all possible paths from the incoming phenotypes to the query phenotype, by a similar process to that described in (1) and (2). **(4)** By accumulating the two datasets obtained in processes (2) and (3), and by selecting unduplicated association rules from the accumulated dataset, the conversion of a PPAP to a pathway-like configuration is completed.

Each converted pathway can be visualized and its constitutive data can be obtained through the application (Supplementary Fig. 3). Note that this pathway represents routes from phenotypes with low numbers of cases of abnormality to phenotypes with many cases of abnormality.

### Constructing a putative phenome-wide phenotype-phenotype association pathway

A putative phenome-wide phenotype-phenotype association pathway was constructed by accumulating 283 sub-pathways from the 345 PPAPs that could be converted to pathway-like configurations, and by removing duplicated association rules from the accumulated dataset. Note that, because 61 sets of PPAPs, where all query phenotypes belonged to the ‘stage/type’ ‘adult/gene’, and a set of PPAPs, where the query phenotype was ‘adult_trait:abnormal_spleen weight (MP)’, had too many associations to convert to pathway-like configuration, 62 PPAP sets were excluded for construction of the putative phenome-wide pathway.

### Extracting communities from overviews of associations between phenotypes and between biological systems by hierarchical clustering

To present an overview of the associations between the 345 phenotypes and to extract communities from that picture, hierarchical clustering, based on the similarities between phenotypes comprising each of the 345 PPAPs, was applied (Fig. 6a). A similar analysis was also applied to the overall picture of associations between the 60 biological systems (Fig. 7). Dendrograms were constructed using both Simpson’s coefficient, with the R package *proxy*, and Ward’s linkage, with the *hclust* function in R or the R package *pvclust*^46^. To make a dendrogram with AU (Approximately Unbiased) *P* values (Fig. 7), which were computed by multiscale bootstrap resampling, both the *pvclust*^46^ and *pvclust-cd* packages were used. The *set* function in the R package *dendextend* was used to change the appearance, color, and size of dendrograms. The *heatmap*.2 function in the R package *gplots* was used to draw heatmaps.

### Presentation of gene sets corresponding to phenotypes in each of the 345 PPAPs

First, all mutant strains (gene symbols), each of which expressed at least one phenotype the same as the PPAP query phenotype, were listed from among the 3,100 mutant strains included in this study. The degree (number and proportion) of overlap between the abnormal phenotypes expressed in each mutant strain (gene) and phenotypes constituting the PPAP of interest was determined, and the listed genes were ranked in descending order of the number of overlapping genes. Datasets of the ranked genes, corresponding to phenotypes in each of the 345 PPAPs (i.e., datasets of phenotype-driven gene-phenotype relationships), are available through the developed application (Supplementary Fig. 3).

### Development of web applications

To construct websites to facilitate understanding of the findings of this study and promote leverage of the obtained datasets as data resources, the package *shiny*, which facilitates the construction of web applications using R, was used to develop three interactive web applications represented in Fig. 1b-ii, Supplementary Figs. 1 and 3, respectively. These web applications use many R packages. For example, the *VisNetwork* package to visualize association rules and the *arules* package to calculate the degree of each node in the graph were used in the development. R Studio’s shinyapps.io hosting service was used to deploy all web applications developed in this study.

## Supplementary information

Supplementary Information.

Supplementary Table 1.

Supplementary Table 2.

Supplementary Table 3.

Supplementary Table 6.

Supplementary Table 8.

Supplementary Table 9.

Supplementary Table 10.

Supplementary Table 11.

Supplementary Table 12.

Supplementary Table 13.

Supplementary Table 14.

Supplementary Table 15.

Supplementary Table 17.

Supplementary Table 23.

Supplementary Table 24.

Supplementary Table 25.

## Data Availability

Data used or generated in this study are available at the following URLs: IMPC dataset (Release 4.3) used for analysis, ftp://ftp.ebi.ac.uk/pub/databases/impc/release-4.3/; Supplementary Data 1–12, 10.6084/m9.figshare.7995911/; website for reviewing optimal conditions for significant rule selection, https://brc-riken.shinyapps.io/effect_size_vs_rules/; website for use and application of phenotypic functional associations obtained from this study, https://brc-riken.shinyapps.io/associations_between_biological_systems/; and website for use and application of phenotypic associations obtained from this study, https://brc-riken.shinyapps.io/phenotypic_associations_across_the_mouse_phenome/. All other data supporting the findings of this study are available within the article and its Supplementary Information files. All source data and R codes for analysis are available from the corresponding author upon reasonable request.
