# Supplementary material for: An atlas of evidence-based phenotypic associations across the mouse phenome
Source: Sci Rep. 2020 Mar 3;10:3957. doi: 10.1038/s41598-020-60891-w (PMC7054260; doi:10.1038/s41598-020-60891-w)
Supplement: Supplementary file 1 — Supplementary Information. [file 41598_2020_60891_MOESM1_ESM.pdf]

Supplementary Information for

An atlas of evidence-based phenotypic associations across the mouse phenome

Nobuhiko Tanaka & Hiroshi Masuya

Integrated Bioresource Information Division, RIKEN BioResource Research Center, Tsukuba, Ibaraki, JAPAN.

Correspondence should be addressed to N. T. (nobuhiko.tanaka@riken.jp)

This supplementary information PDF file includes:

Supplementary Fig. 1 -- 2
Supplementary Fig. 2 -- 3
Supplementary Fig. 3 -- 5
Supplementary Fig. 4 -- 7
Titles of Supplementary Tables (excel files) 1–3, 6, 8–15, 17, 23, 24, and 25 ---------------------- 8
Supplementary Tables 4, 5, 7, 16, and 18–22 -- 9
Supplementary Data 13 -- 19
Supplementary Data 14 -- 44
Supplementary Data 15 -- 48
Supplementary Methods --- 59
References --- 61

Supplementary Fig. 1

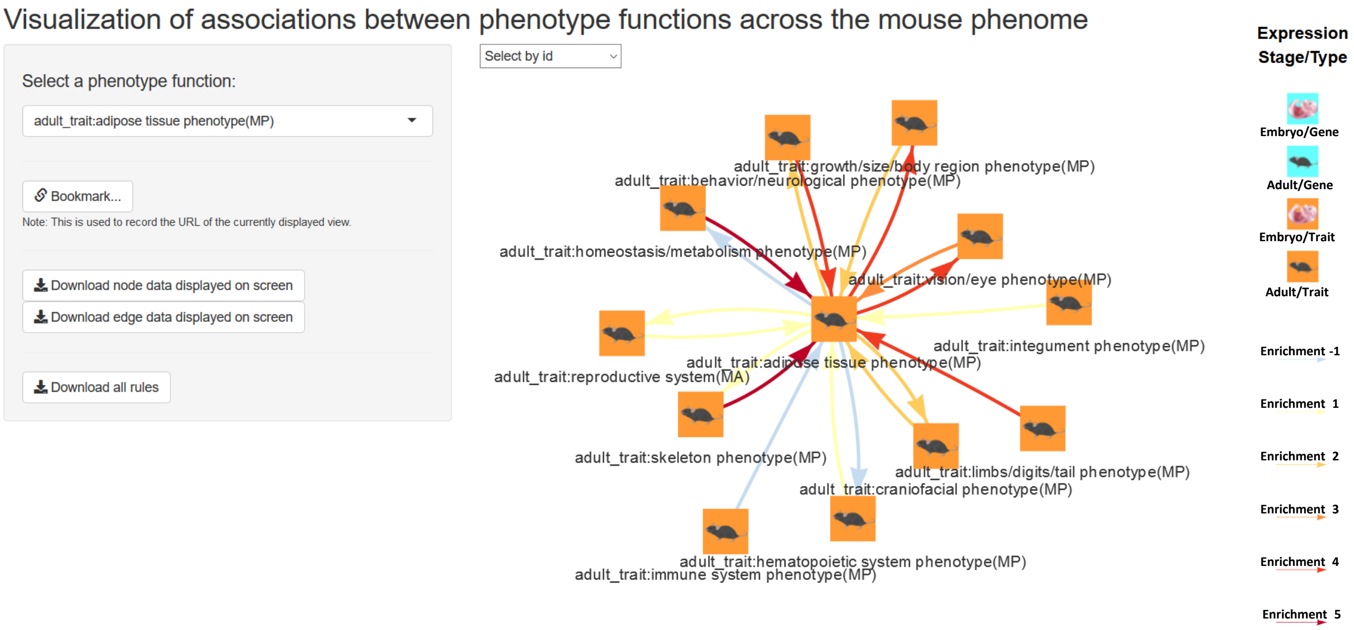

Supplementary Fig. 1 A viewer to display associations between biological systems across the mouse phenome. When selecting a biological system of interest from 60 distinct biological systems using the pull-down menu on the sidebar panel, all the between-‘biological systems’ relationships related to the selected biological system are interactively displayed on the main panel. Darker arrow colors for enrichments 1 to 5 (-1 to -5) indicate a greater degree of positive (negative) enrichment in each between-‘biological systems’ relationship. Data displayed, or all between-‘biological systems’ relationships, can be obtained by pressing the corresponding button on the sidebar panel. This viewer is available at https://brc-riken.shinyapps.io/associations_between_biological_systems/.

Supplementary Fig. 2

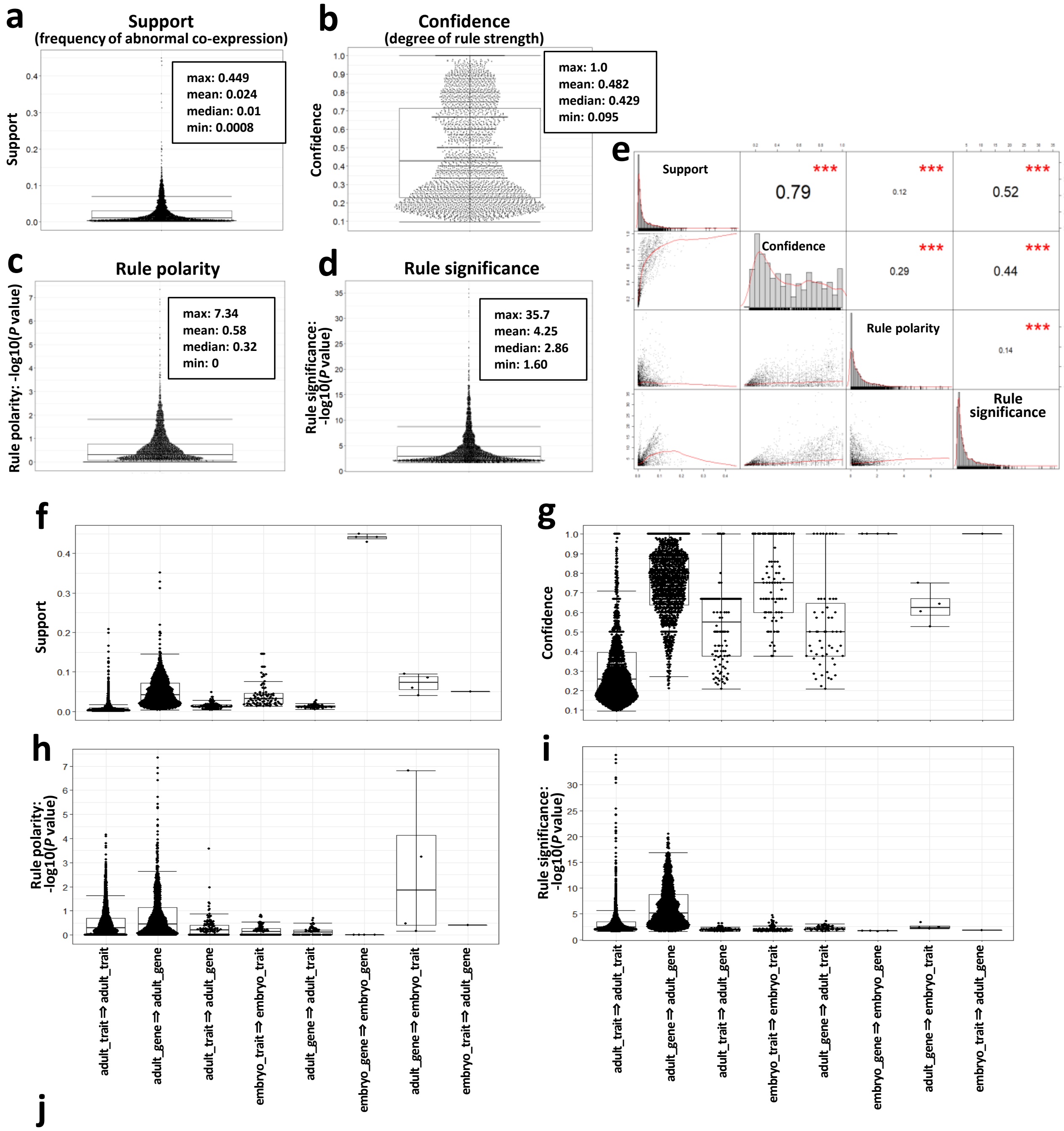

j

	Comparisons between stage_type rule categorizations			Sample size Left	:	Right	*P* values by post-hoc test in each of the measures for association rules: Support	Confidence	Rule polarity	Rule significance
1	adult_trait ⇒ adult_trait	:	adult_gene ⇒ adult_gene	2,194	:	1,230	$<10^{-15}$ ***	$<10^{-15}$ ***	5.4×10^{-12} ***	2.2×10^{-11} ***
2	adult_trait ⇒ adult_trait	:	adult_trait ⇒ adult_gene	2,194	:	112	2.0×10^{-10} ***	6.9×10^{-14} ***	3.0×10^{-4} ***	5.2×10^{-12} ***
3	embryo_trait ⇒ embryo_trait	:	adult_trait ⇒ adult_trait	86	:	2,194	2.8×10^{-10} ***	4.7×10^{-10} ***	5.0×10^{-14} ***	1.6×10^{-14} ***
4	adult_trait ⇒ adult_trait	:	adult_gene ⇒ adult_trait	2,194	:	55	5.7×10^{-7} ***	8.8×10^{-8} ***	4.7×10^{-10} ***	1.0×10^{-15} ***
5	adult_trait ⇒ adult_gene	:	adult_gene ⇒ adult_gene	112	:	1,230	$<10^{-15}$ ***	$<10^{-15}$ ***	1.0×10^{-13} ***	$<10^{-15}$ ***
6	embryo_trait ⇒ embryo_trait	:	adult_gene ⇒ adult_gene	86	:	1,230	0.12	1	$<10^{-15}$ ***	$<10^{-15}$ ***
7	adult_gene ⇒ adult_trait	:	adult_gene ⇒ adult_gene	55	:	1,230	$<10^{-15}$ ***	4.4×10^{-8} ***	9.3×10^{-13} ***	3.9×10^{-13} ***
8	embryo_trait ⇒ embryo_trait	:	adult_trait ⇒ adult_gene	86	:	112	5.7×10^{-7} ***	9.3×10^{-13} ***	4.4×10^{-2} *	0.2
9	adult_trait ⇒ adult_gene	:	adult_gene ⇒ adult_trait	112	:	55	0.25	1	7.7×10^{-3} **	1.4×10^{-3} **
10	embryo_trait ⇒ embryo_trait	:	adult_gene ⇒ adult_trait	86	:	55	4.3×10^{-10} ***	2.6×10^{-7} ***	0.9	0.89

Supplementary Fig. 2 Examination of the four interest measures for the 3,686 significant association rules. (**a–e**) Distributions of values for support (**a**), confidence (**b**), rule polarity (**c**), and rule significance (**d**) for the 3,686 rules and the statistical dependence between the four measures (**e**) are shown. Spearman's correlation coefficients (Rs) between the four measures were computed using the R package *PerformanceAnalytics*. The three red stars in **e** indicate that the probability is practically nil. (**f–i**) Comparisons of values for the four measures in each of 3,686 significant association rules according to between-'stage/type' rules. Distributions of values for each of the four measures of support (**f**), confidence (**g**), rule polarity (**h**), and rule significance (**i**) are shown according to eight between-'stage/type' rules. (**j**) A summary table of comparison test results for each of the four measures according to between-'stage/type' rule categories. *P* values for each of the four measures for all comparable pairwise combinations of between-'stage/type' rule categories were calculated using the Games-Howell post-hoc test. The relationships among 'stage/type' rule categories are arranged in descending order of the sum of the number of 'between-phenotypes' rules constituting their relationships. **P* < 0.05, ***P* < 0.01, ****P* < 0.001. Refer to **Supplementary Data 13** for details.

Supplementary Fig. 3

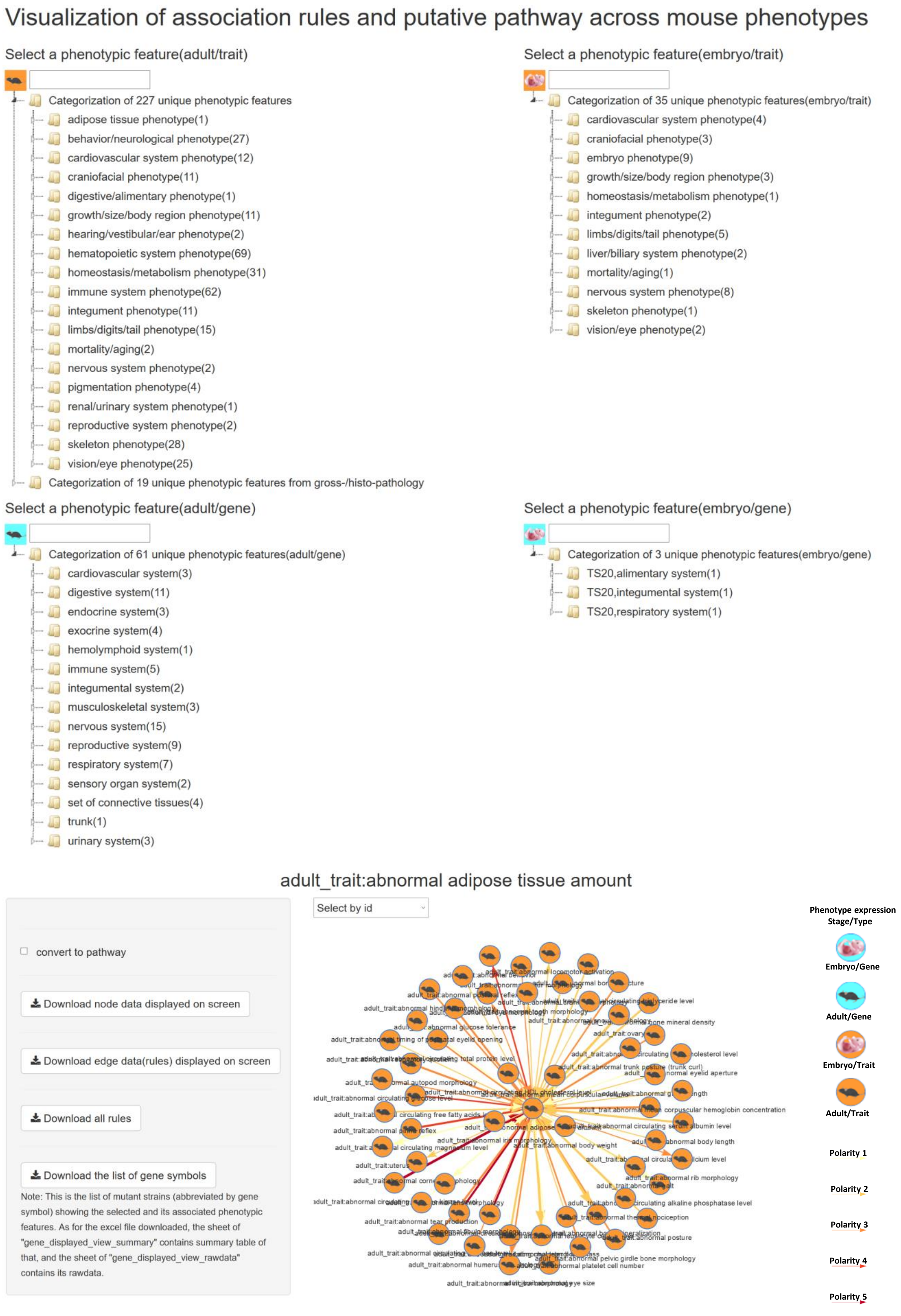

Supplementary Fig. 3 Visualization of both a phenotype-phenotype association pair set (PPAP) and its pathway-like configuration. A web application tool was developed to facilitate use of the 3,686 significant association rules identified in this study for various biological studies (https://brc-riken.shinyapps.io/phenotypic_associations_across_the_mouse_phenome/). A sample screenshot of the application is presented here. The 345 phenotypes constituting the 3,686 association rules are classified into four 'stage/type' categories ('adult/trait', 'adult/gene', 'embryo/trait', 'embryo/gene') of phenotypic expression and are displayed in a tree view according to 60 biological systems (above). When a phenotype of interest (in this case, 'adult_trait:abnormal adipose tissue amount') is selected from the above tree, associations between the selected phenotype and its related phenotypes (PPAP or phenotypic expression module) are interactively displayed (below). When the mouse pointer is positioned over the query phenotype in the middle of the PPAP, the number of incoming phenotypes (indegree), the number of outgoing phenotypes (outdegree), the sum of indegree and outdegree (i.e., degree), and the number of distinct biological systems for phenotypes belonging to the displayed PPAP are designed to appear during the mouse over. By contrast, when the mouse pointer is positioned over an edge (representing a relationship between phenotypes), values of support, confidence, confidence for another in the bidirectional rule, lift, rule polarity, and rule significance are designed to appear during the mouse over (refer to Methods for detailed descriptions of these measures). Wider edge lines represent larger confidence values, whereas darker edge color represents larger rule polarity value. Information on nodes (phenotypes) and edges (relationships) in a displayed PPAP can be acquired by clicking their corresponding buttons, arranged in the bottom left panel. Further, data on the 3,686 association rules can be acquired by clicking the button 'Download all rules' on the bottom left panel. By clicking the uppermost check box on the bottom left panel, a displayed PPAP is interactively converted into a pathway-like configuration (refer to **Fig. 4f** and Methods). In addition, a list of gene symbols, related to phenotypes within a displayed PPAP, can be downloaded by clicking the button 'Download the list of gene symbols' on the bottom left panel.

Supplementary Fig. 4

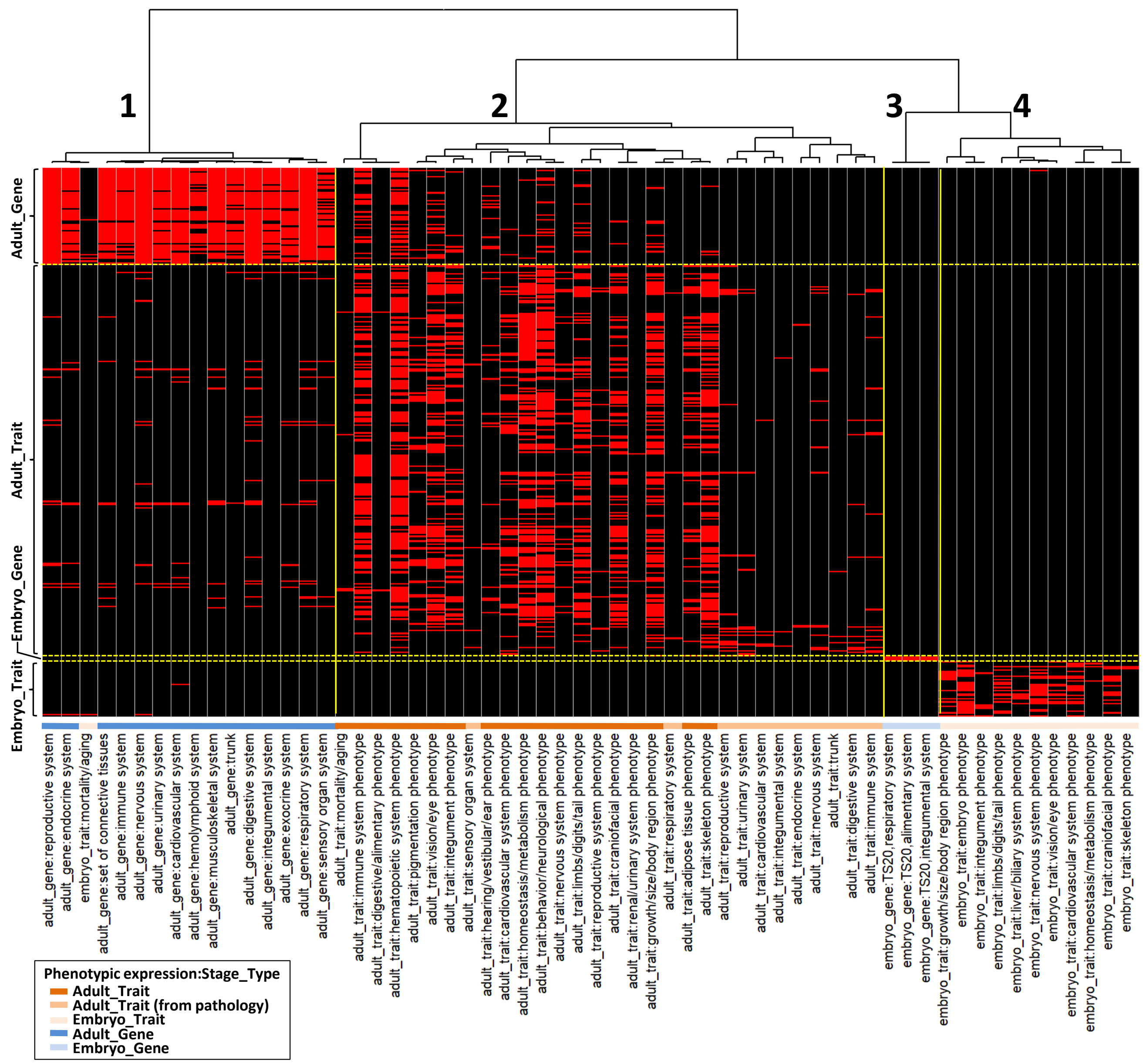

Supplementary Fig. 4 Heatmap with dendrogram showing overall relationships among 60 biological systems. The dendrogram was constructed by hierarchical clustering, based on the similarity of biological systems for phenotypes constituting each of the 345 PPAPs. Hierarchical clustering was conducted using Simpson distance and Ward linkage. The longitudinal and horizontal axes represent 345 PPAPs and PPAP-constituting distinct biological systems, respectively. Note that only relationships between biological systems are clustered. Clustered biological systems are colored by their phenotypic expression 'stage/type' category. In the heatmap, red and black represent positive and negative, respectively, for the existence of phenotypic abnormality at the biological system level. The order of labels on both axes is shown in **Supplementary Table 25**.

Supplementary Table 1 532 phenotypes used for association rule mining (excel file)

Supplementary Table 2 Statistics of various measures of the 3,686 significant association rules (excel file)

Supplementary Table 3 Analysis summary for 345 phenotypes constituting the 3,686 significant association rules (excel file)

Supplementary Table 6 Statistics of various measures for the 608 between-'biological systems' rules (excel file)

Supplementary Table 8 Multiple comparison analysis of values in each of four measures by between-'stage/type' rule categories (excel file)

Supplementary Table 9 Multiple comparison analysis of values in each of four measures by between-'biological systems' rules belonging to the between-'stage/type' rule 'adult_trait => adult_trait' (excel file)

Supplementary Table 10 Multiple comparison analysis of values in each of four measures by between-'biological systems' rules belonging to the between-'stage/type' rule 'adult_gene => adult_gene' (excel file)

Supplementary Table 11 Multiple comparison analysis of values in each of four measures by between-'biological systems' rules belonging to the between-'stage/type' rule 'adult_trait => adult_gene' (excel file)

Supplementary Table 12 Multiple comparison analysis of values in each of four measures by between-'biological systems' rules belonging to the between-'stage/type' rule 'embryo_trait => embryo_trait' (excel file)

Supplementary Table 13 Multiple comparison analysis of values in each of four measures by between-'biological systems' rules belonging to the between-'stage/type' rule 'adult_gene => adult_trait' (excel file)

Supplementary Table 14 The number of phenotypes and distinct biological systems in each of 345 PPAPs according to PPAP query phenotype 'stage/type' category (excel file)

Supplementary Table 15 Multiple comparison analysis of both the number of phenotypes and the number of distinct biological systems in 345 PPAPs by PPAP query phenotype 'stage/type' category (excel file)

Supplementary Table 17 Multiple comparison analysis of both the number of phenotypes and the number of distinct biological systems in each of the 345 PPAPs by biological systems of PPAP query phenotypes (excel file). A series of conventional analyses for these multiple comparisons were performed for each PPAP query phenotype 'stage/type' category.

Supplementary Table 23 Enrichment analysis of less frequently/more frequently occurring phenotypes by 'stage/type' and 'biological systems' phenotypic expression categories (excel file)

Supplementary Table 24 Phenotypes (nodes; n = 335) and associations (edges; n = 1,549) in the putative phenome-wide phenotype-phenotype association pathway (excel file)

Supplementary Table 25 Information about relationships among biological systems across the mouse phenome determined by hierarchical clustering (excel file)

Supplementary Table 4 The number of phenotypes before and after rule selection by biological system categorization

Phenotypic expression stage/type	Top level term (biological system)	Top level id	Number of phenotypes before selection	Number of phenotypes after selection	*P* value	Bonferroni
adult/trait	hematopoietic system phenotype	MP:0005397	79	69	0.130	1
	immune system phenotype	MP:0005387	74	62	0.197	1
	homeostasis/metabolism phenotype	MP:0005376	42	31	0.712	1
	behavior/neurological phenotype	MP:0005386	38	27	0.795	1
	vision/eye phenotype	MP:0005391	32	25	0.580	1
	skeleton phenotype	MP:0005390	28	28	0.162	1
	cardiovascular system phenotype	MP:0005385	22	12	0.723	1
	limbs/digits/tail phenotype	MP:0005371	15	15	0.264	1
	growth/size/body region phenotype	MP:0005378	14	11	0.684	1
	integument phenotype	MP:0010771	14	11	0.684	1
	craniofacial phenotype	MP:0005382	13	11	0.537	1
	renal/urinary system phenotype	MP:0005367	7	1	0.157	1
	pigmentation phenotype	MP:0001186	4	4	0.720	1
	respiratory system phenotype	MP:0005388	4	0	0.157	1
	digestive/alimentary phenotype	MP:0005381	3	1	1	1
	hearing/vestibular/ear phenotype	MP:0005377	3	2	1	1
	nervous system phenotype	MP:0003631	3	2	1	1
	adipose tissue phenotype	MP:0005375	2	1	1	1
	mortality/aging	MP:0010768	2	2	0.652	1
	muscle phenotype	MP:0005369	2	0	0.521	1
	reproductive system phenotype	MP:0005389	2	2	0.652	1
adult/trait from gross-/histo-pathology	digestive system	MA:0002431	9	2	0.217	1
	reproductive system	MA:0000326	6	5	0.762	1
	musculoskeletal system	MA:0002418	5	0	0.163	1
	respiratory system	MA:0000327	5	1	0.412	1
	immune system	MA:0002711	4	3	1	1
	sensory organ system	MA:0002442	4	1	0.654	1
	exocrine system	MA:0002411	3	0	0.281	1
	nervous system	MA:0000016	3	1	1	1
	endocrine system	MA:0000012	2	1	1	1
	integumental system	MA:0000014	2	1	1	1
	urinary system	MA:0000325	2	2	0.652	1
	cardiovascular system	MA:0000010	1	1	1	1
	hemolymphoid system	MA:0000013	1	0	1	1
	trunk	MA:0000004	1	1	1	1
	Total		451	336		
adult/gene	digestive system	MA:0002431	17	11	1	1
	nervous system	MA:0000016	17	15	0.466	1
	reproductive system	MA:0000326	11	9	0.651	1
	respiratory system	MA:0000327	8	7	0.604	1
	exocrine system	MA:0002411	5	4	0.747	1
	immune system	MA:0002711	5	5	0.531	1
	set of connective tissues	MA:0002887	4	4	0.720	1
	cardiovascular system	MA:0000010	3	3	0.687	1
	endocrine system	MA:0000012	3	3	0.687	1
	musculoskeletal system	MA:0002418	3	3	0.687	1
	sensory organ system	MA:0002442	3	2	1	1
	urinary system	MA:0000325	3	3	0.687	1
	integumental system	MA:0000014	2	2	0.652	1
	hemolymphoid system	MA:0000013	1	1	1	1
	limb	MA:0000007	1	0	1	1
	trunk	MA:0000004	1	1	1	1
	Total		87	73		
embryo/trait	embryo phenotype	MP:0005380	20	9	0.443	1
	nervous system phenotype	MP:0003631	10	8	0.809	1
	cardiovascular system phenotype	MP:0005385	9	4	0.581	1
	growth/size/body region phenotype	MP:0005378	9	3	0.383	1
	craniofacial phenotype	MP:0005382	7	3	0.748	1
	limbs/digits/tail phenotype	MP:0005371	6	5	0.762	1
	integument phenotype	MP:0010771	4	2	1	1
	vision/eye phenotype	MP:0005391	4	2	1	1
	homeostasis/metabolism phenotype	MP:0005376	3	1	1	1
	behavior/neurological phenotype	MP:0005386	2	0	0.521	1
	digestive/alimentary phenotype	MP:0005381	2	0	0.521	1
	hearing/vestibular/ear phenotype	MP:0005377	2	0	0.521	1
	liver/biliary system phenotype	MP:0005370	2	2	0.652	1
	mortality/aging	MP:0010768	1	1	1	1
	respiratory system phenotype	MP:0005388	1	0	1	1
	skeleton phenotype	MP:0005390	1	1	1	1
	Total		83	41		
embryo/gene	TS20,limb	EMAP:4011	11	0	0.005	0.38
	TS20,alimentary system	EMAP:3983	9	1	0.099	1
	TS20,cardiovascular system	EMAP:4365	7	0	0.047	1
	TS20,nervous system	EMAP:4124	7	0	0.047	1
	TS20,sensory organ	EMAP:4285	7	0	0.047	1
	TS20,musculoskeletal system	EMAP:33504	5	0	0.163	1
	TS20,mesenchyme	EMAP:4103	3	0	0.281	1
	TS20,head	EMAP:31902	2	0	0.521	1
	TS20,integumental system	EMAP:4146	2	1	1	1
	TS20,reproductive system	EMAP:4596	2	0	0.521	1
	TS20,respiratory system	EMAP:4602	2	1	1	1
	TS20,tail	EMAP:4651	2	0	0.521	1
	TS20,urinary system	EMAP:4580	2	0	0.521	1
	TS20,embryo	EMAP:3980	1	0	1	1
	TS20,liver and biliary system	EMAP:4570	1	0	1	1
	TS20,notochord	EMAP:4109	1	0	1	1
	TS20,trunk	EMAP:31877	1	0	1	1
	Total		65	3		

Note that one phenotype could have more than one top level term (biological system). The *P* values shown above were calculated by two-tailed Fisher's exact test.

Supplementary Table 5 Enrichment analysis of the 3,686 association rules by stage/type rule categorization

Lhs_stage_type	Direction	Rhs_stage_type	Theoretical ratio	Practical ratio	Fold enrichment	*P* value	Corrected *P*
adult_gene	⇒	adult_gene	5,256/265,036	1,230/3,686	16.8	0	0 **
adult_trait	⇒	adult_trait	108,570/265,036	2,194/3,686	1.45	1.7×10^{-41}	1.5×10^{-40}**
embryo_trait	⇒	embryo_trait	4,422/265,036	86/3,686	1.40	3.7×10^{-3}	3.7×10^{-3}*
adult_trait	⇒	adult_gene	24,090/265,036	112/3,686	0.334	4.8×10^{-42}	4.8×10^{-41}**
adult_gene	⇒	adult_trait	24,090/265,036	55/3,686	0.164	4.1×10^{-75}	4.5×10^{-74}**
embryo_gene	⇒	embryo_gene	3,782/265,036	4/3,686	0.076	1.9×10^{-17}	3.7×10^{-17}**
adult_gene	⇒	embryo_trait	4,891/265,036	4/3,686	0.059	1.2×10^{-23}	3.6×10^{-23}**
embryo_trait	⇒	adult_gene	4,891/265,036	1/3,686	0.015	1.2×10^{-27}	9.6×10^{-27}**
adult_trait	⇒	embryo_trait	22,110/265,036	0/3,686	0	7.2×10^{-128}	1.1×10^{-126}**
embryo_trait	⇒	adult_trait	22,110/265,036	0/3,686	0	7.2×10^{-128}	1.1×10^{-126}**
adult_trait	⇒	embryo_gene	20,460/265,036	0/3,686	0	1.2×10^{-118}	1.6×10^{-117}**
embryo_gene	⇒	adult_trait	20,460/265,036	0/3,686	0	1.2×10^{-118}	1.6×10^{-117}**
adult_gene	⇒	embryo_gene	4,526/265,036	0/3,686	0	2.8×10^{-27}	2.0×10^{-26}**
embryo_gene	⇒	adult_gene	4,526/265,036	0/3,686	0	2.8×10^{-27}	2.0×10^{-26}**
embryo_trait	⇒	embryo_gene	4,154/265,036	0/3,686	0	4.0×10^{-25}	2.0×10^{-24}**
embryo_gene	⇒	embryo_trait	4,154/265,036	0/3,686	0	4.0×10^{-25}	2.0×10^{-24}**

P values were calculated by two-tailed Fisher's exact test. A single asterisk indicates significant enrichment at Holm's corrected $P < 0.01$, and two asterisks at Holm's corrected $P < 0.001$. The direction (positive/negative) of the enrichment is presented, depending on whether the value of fold enrichment is greater than 1.

Supplementary Table 7 Enrichment analysis of 'between-phenotypes' rules with greater/smaller values for each of four measures by stage/type rule categorization

Measures for rules	High/Low	Stage_type rule categorization Lhs	Direction	Rhs	Ratio in the selected subset	Ratio in total	Fold enrichment	*P* value	Corrected *P*
support	High	adult_gene	⇒	adult_gene	156/185	1,230/3,686	2.53	1.19E-15***	4.74E-15***
support	High	embryo_gene	⇒	embryo_gene	4/185	4/3,686	19.92	0.000328***	0.00131**
support	High	embryo_trait	⇒	embryo_trait	9/185	86/3,686	2.09	0.04116*	0.16464
support	High	adult_gene	⇒	embryo_trait	1/185	4/3,686	4.98	0.218091	0.872362
support	High	adult_trait	⇒	adult_trait	15/185	2,194/3,686	0.14		
support	Low	adult_trait	⇒	adult_trait	185/185	2,194/3,686	1.68	9.81E-07***	
confidence	High	adult_gene	⇒	adult_gene	113/185	1,230/3,686	1.83	1.3E-06***	5.2E-06***
confidence	High	embryo_trait	⇒	embryo_trait	16/185	86/3,686	3.71	4.03E-05***	0.000161***
confidence	High	embryo_gene	⇒	embryo_gene	4/185	4/3,686	19.92	0.000328***	0.00131**
confidence	High	adult_gene	⇒	adult_trait	8/185	55/3,686	2.90	0.011026*	0.044106
confidence	High	adult_trait	⇒	adult_gene	5/185	112/3,686	0.89		
confidence	High	adult_trait	⇒	adult_trait	39/185	2,194/3,686	0.35		
confidence	Low	adult_trait	⇒	adult_trait	185/185	2,194/3,686	1.68	9.81E-07***	
polarity	High	adult_gene	⇒	adult_gene	198/324	1,230/3,686	1.83	4.87E-10***	9.74E-10***
polarity	High	adult_gene	⇒	embryo_trait	2/324	4/3,686	5.69	0.079188	0.158375
polarity	High	adult_trait	⇒	adult_trait	122/324	2,194/3,686	0.63		
polarity	High	adult_trait	⇒	adult_gene	2/324	112/3,686	0.20		
polarity	Low	embryo_trait	⇒	embryo_trait	33/715	86/3,686	1.98	0.001288**	0.006442**
polarity	Low	adult_gene	⇒	adult_trait	24/715	55/3,686	2.25	0.001443**	0.007215**
polarity	Low	adult_trait	⇒	adult_gene	37/715	112/3,686	1.70	0.005427**	0.027135*
polarity	Low	embryo_gene	⇒	embryo_gene	3/715	4/3,686	3.87	0.090371	0.451855
polarity	Low	adult_trait	⇒	adult_trait	442/715	2,194/3,686	1.04	0.294814	1
polarity	Low	adult_gene	⇒	adult_gene	176/715	1,230/3,686	0.74		
rule significance	High	adult_gene	⇒	adult_gene	151/185	1,230/3,686	2.45	1.85E-14***	
rule significance	High	adult_trait	⇒	adult_trait	34/185	2,194/3,686	0.31		
rule significance	Low	embryo_trait	⇒	embryo_trait	27/185	86/3,686	6.26	1.2E-11***	4.82E-11***
rule significance	Low	adult_trait	⇒	adult_gene	24/185	112/3,686	4.27	7.7E-08***	3.08E-07***
rule significance	Low	embryo_gene	⇒	embryo_gene	4/185	4/3,686	19.92	0.000328***	0.00131**
rule significance	Low	adult_gene	⇒	adult_trait	4/185	55/3,686	1.45	0.315073	1
rule significance	Low	adult_trait	⇒	adult_trait	100/185	2,194/3,686	0.91		
rule significance	Low	adult_gene	⇒	adult_gene	26/185	1,230/3,686	0.42		

For the three measures support, confidence, and rule significance, enrichment analyses were performed for 185 association rules with the top or bottom 5% of values for each of the three measures. For the measure rule polarity, enrichment analyses were performed for 324 association rules identified as having greater values (FDR < 0.2, uncorrected *P* value < 0.0195), and also for 715 association rules identified as having smaller values (uncorrected *P* value = 1). *P* values were calculated by one-tailed Fisher's exact test. A single asterisk, two asterisks, and three asterisks represent significant enrichment at *P* < 0.05, *P* < 0.01, and *P* < 0.001, respectively. Corrected *P* values are *P* values after Bonferroni correction for multiple testing. See **Supplementary Data 13** for detailed explanations of these analyses. The results obtained here are summarized in **Fig. 3a**.

Supplementary Table 16 Enrichment analysis of both the number of phenotypes and the number of distinct biological systems in each of 345 PPAPs by 'stage/type' phenotypic categorization of PPAP query phenotypes

Measures examined	'Stage_Type' categorization of query phenotype	Ratio in the selected subset	Ratio in total	Fold enrichment	*P* value
Num. of phenotypes	adult_trait	15/35	246/345	0.6	2.8×10^{-4}***
	adult_gene	20/35	61/345	3.2	
	embryo_trait	0/35	35/345	0	
	embryo_gene	0/35	3/345	0	
Num. of distinct biological systems	adult_trait	8/40	246/345	0.3	8.9×10^{-8}***
	adult_gene	32/40	61/345	4.5	
	embryo_trait	0/40	35/345	0	
	embryo_gene	0/40	3/345	0	

For the number of phenotypes and distinct biological systems in each of 345 PPAPs, enrichment analyses were performed for association rules with the top 10% of values in each of the two measures. *P* values were calculated by one-tailed Fisher's exact test. Three asterisks (***) represent significant enrichment at $P < 0.001$.

Supplementary Table 18 List of PPAPs including the maximum number of phenotypes by biological systems of PPAP query phenotypes

Stage_type:top level term	Maximum number of phenotypes	Corresponding query phenotype (stage_type:ontology name)
adult_trait:growth/size/body region phenotype(MP)	82	adult_trait:abnormal lean body mass
adult_trait:hematopoietic system phenotype(MP)	77	adult_trait:abnormal mean corpuscular volume
adult_trait:skeleton phenotype(MP)	70	adult_trait:abnormal bone mineralization
adult_trait:craniofacial phenotype(MP)	68	adult_trait:abnormal cranium morphology
adult_trait:homeostasis/metabolism phenotype(MP)	66	adult_trait:abnormal circulating alkaline phosphatase level
adult_trait:adipose tissue phenotype(MP)	57	adult_trait:abnormal adipose tissue amount
adult_trait:vision/eye phenotype(MP)	56	adult_trait:abnormal cornea morphology|adult_trait:abnormal eyelid aperture
adult_trait:behavior/neurological phenotype(MP)	55	adult_trait:abnormal locomotor activation
adult_trait:limbs/digits/tail phenotype(MP)	52	adult_trait:abnormal tibia morphology
adult_trait:integument phenotype(MP)	37	adult_trait:abnormal thermal nociception
adult_trait:immune system phenotype(MP)	35	adult_trait:abnormal CD4-positive, alpha beta T cell number
adult_trait:cardiovascular system phenotype(MP)	33	adult_trait:abnormal retinal vasculature morphology
adult_trait:hearing/vestibular/ear phenotype(MP)	24	adult_trait:abnormal auditory brainstem response
adult_trait:nervous system phenotype(MP)	16	adult_trait:abnormal optic disc morphology
adult_trait:pigmentation phenotype(MP)	16	adult_trait:abnormal coat/hair pigmentation|adult_trait:abnormal eye pigmentation
adult_trait:reproductive system phenotype(MP)	6	adult_trait:abnormal fertility/fecundity|adult_trait:abnormal external genitalia morphology
adult_trait:digestive/alimentary phenotype(MP)	2	adult_trait:abnormal susceptibility to colitis induced morbidity/mortality
adult_trait:mortality/aging(MP)	2	adult_trait:abnormal susceptibility to colitis induced morbidity/mortality| adult_trait:abnormal survival/lethality
adult_trait:renal/urinary system phenotype(MP)	2	adult_trait:abnormal kidney weight
adult_trait:nervous system(MA)	14	adult_trait:brain
adult_trait:immune system(MA)	13	adult_trait:spleen
adult_trait:reproductive system(MA)	11	adult_trait:ovary
adult_trait:urinary system(MA)	11	adult_trait:kidney
adult_trait:digestive system(MA)	9	adult_trait:liver
adult_trait:cardiovascular system(MA)	5	adult_trait:heart
adult_trait:sensory organ system(MA)	4	adult_trait:eye
adult_trait:trunk(MA)	4	adult_trait:pancreas
adult_trait:integumental system(MA)	3	adult_trait:skin
adult_trait:endocrine system(MA)	3	adult_trait:adrenal gland
adult_trait:respiratory system(MA)	3	adult_trait:lung
adult_gene:cardiovascular system(MA)	65	adult_gene:aorta
adult_gene:digestive system(MA)	62	adult_gene:small intestine
adult_gene:nervous system(MA)	60	adult_gene:pituitary gland
adult_gene:reproductive system(MA)	57	adult_gene:oviduct
adult_gene:set of connective tissues(MA)	55	adult_gene:brown adipose tissue
adult_gene:urinary system(MA)	55	adult_gene:kidney
adult_gene:endocrine system(MA)	52	adult_gene:thyroid gland|adult_gene:adrenal gland
adult_gene:musculoskeletal system(MA)	52	adult_gene:skeletal muscle tissue
adult_gene:respiratory system(MA)	52	adult_gene:lung|adult_gene:trachea
adult_gene:trunk(MA)	50	adult_gene:pancreas
adult_gene:integumental system(MA)	49	adult_gene:skin
adult_gene:immune system(MA)	46	adult_gene:thymus
adult_gene:exocrine system(MA)	44	adult_gene:prostate gland|adult_gene:mammary gland
adult_gene:hemolymphoid system(MA)	40	adult_gene:peyer's patch
adult_gene:sensory organ system(MA)	34	adult_gene:eye
embryo_trait:embryo phenotype(MP)	13	embryo_trait:abnormal neural tube closure
embryo_trait:nervous system phenotype(MP)	13	embryo_trait:abnormal neural tube closure
embryo_trait:craniofacial phenotype(MP)	12	embryo_trait:abnormal craniofacial morphology
embryo_trait:vision/eye phenotype(MP)	12	embryo_trait:abnormal eye morphology
embryo_trait:limbs/digits/tail phenotype(MP)	11	embryo_trait:abnormal limb bud morphology
embryo_trait:cardiovascular system phenotype(MP)	9	embryo_trait:abnormal pericardium morphology
embryo_trait:growth/size/body region phenotype(MP)	8	embryo_trait:abnormal embryo size
embryo_trait:liver/biliary system phenotype(MP)	8	embryo_trait:abnormal liver morphology
embryo_trait:mortality/aging(MP)	5	embryo_trait:abnormal viability by preweaning
embryo_trait:integument phenotype(MP)	4	embryo_trait:abnormal skin appearance
embryo_trait:homeostasis/metabolism phenotype(MP)	3	embryo_trait:abnormal fluid regulation
embryo_trait:skeleton phenotype(MP)	2	embryo_trait:abnormal cranium morphology
embryo_gene:TS20,alimentary system(EMAP)	3	embryo_gene:TS20,oral cavity
embryo_gene:TS20,integumental system(EMAP)	3	embryo_gene:TS20,skin
embryo_gene:TS20,respiratory system(EMAP)	3	embryo_gene:TS20,lung

Supplementary Table 19 List of PPAPs including the maximum number of distinct biological systems according to biological system of PPAP query phenotype

Stage_type:top level term	Maximum number of functions (biological systems)	Corresponding query phenotype (stage_type:ontology name)
adult_trait:hematopoietic system phenotype(MP)	24	adult_trait:abnormal mean corpuscular volume
adult_trait:vision/eye phenotype(MP)	22	adult_trait:abnormal cornea morphology
adult_trait:behavior/neurological phenotype(MP)	17	adult_trait:abnormal behavior|adult_trait:abnormal startle reflex|adult_trait:abnormal grip strength
adult_trait:limbs/digits/tail phenotype(MP)	17	adult_trait:abnormal digit morphology
adult_trait:growth/size/body region phenotype(MP)	17	adult_trait:abnormal lean body mass
adult_trait:skeleton phenotype(MP)	16	adult_trait:abnormal bone mineralization|adult_trait:abnormal bone mineral density
adult_trait:homeostasis/metabolism phenotype(MP)	16	adult_trait:abnormal circulating alkaline phosphatase level
adult_trait:cardiovascular system phenotype(MP)	16	adult_trait:abnormal retinal vasculature morphology
adult_trait:craniofacial phenotype(MP)	15	adult_trait:abnormal cranium morphology
adult_trait:hearing/vestibular/ear phenotype(MP)	14	adult_trait:abnormal auditory brainstem response
adult_trait:integument phenotype(MP)	13	adult_trait:abnormal coat appearance
adult_trait:pigmentation phenotype(MP)	12	adult_trait:abnormal coat/hair pigmentation
adult_trait:adipose tissue phenotype(MP)	12	adult_trait:abnormal adipose tissue amount
adult_trait:immune system phenotype(MP)	11	adult_trait:abnormal spleen weight
adult_trait:nervous system phenotype(MP)	10	adult_trait:abnormal optic disc morphology
adult_trait:reproductive system phenotype(MP)	5	adult_trait:abnormal fertility/fecundity|adult_trait:abnormal external genitalia morphology
adult_trait:digestive/alimentary phenotype(MP)	4	adult_trait:abnormal susceptibility to colitis induced morbidity/mortality
adult_trait:mortality/aging(MP)	4	adult_trait:abnormal susceptibility to colitis induced morbidity/mortality
adult_trait:renal/urinary system phenotype(MP)	3	adult_trait:abnormal kidney weight
adult_trait:immune system(MA)	11	adult_trait:spleen
adult_trait:nervous system(MA)	10	adult_trait:brain
adult_trait:urinary system(MA)	7	adult_trait:kidney
adult_trait:reproductive system(MA)	6	adult_trait:testis
adult_trait:digestive system(MA)	6	adult_trait:liver
adult_trait:cardiovascular system(MA)	5	adult_trait:heart
adult_trait:sensory organ system(MA)	5	adult_trait:eye
adult_trait:trunk(MA)	4	adult_trait:pancreas
adult_trait:endocrine system(MA)	3	adult_trait:adrenal gland
adult_trait:integumental system(MA)	3	adult_trait:skin
adult_trait:respiratory system(MA)	3	adult_trait:lung
adult_gene:cardiovascular system(MA)	22	adult_gene:aorta
adult_gene:digestive system(MA)	21	adult_gene:small intestine
adult_gene:respiratory system(MA)	20	adult_gene:lung
adult_gene:reproductive system(MA)	20	adult_gene:uterus
adult_gene:nervous system(MA)	19	adult_gene:pituitary gland
adult_gene:endocrine system(MA)	19	adult_gene:thyroid gland
adult_gene:exocrine system(MA)	18	adult_gene:prostate gland
adult_gene:set of connective tissues(MA)	18	adult_gene:cartilage tissue|adult_gene:white adipose tissue
adult_gene:musculoskeletal system(MA)	18	adult_gene:skeletal muscle tissue
adult_gene:urinary system(MA)	17	adult_gene:kidney|adult_gene:lower urinary tract
adult_gene:trunk(MA)	17	adult_gene:pancreas
adult_gene:immune system(MA)	16	adult_gene:thymus
adult_gene:sensory organ system(MA)	15	adult_gene:eye
adult_gene:integumental system(MA)	15	adult_gene:skin|adult_gene:mammary gland
adult_gene:hemolymphoid system(MA)	13	adult_gene:peyer's patch
embryo_trait:craniofacial phenotype(MP)	9	embryo_trait:abnormal craniofacial morphology
embryo_trait:vision/eye phenotype(MP)	8	embryo_trait:abnormal eye morphology
embryo_trait:nervous system phenotype(MP)	7	embryo_trait:abnormal midbrain development|embryo_trait:abnormal hindbrain development
embryo_trait:liver/biliary system phenotype(MP)	7	embryo_trait:abnormal liver morphology
embryo_trait:embryo phenotype(MP)	6	embryo_trait:abnormal neural tube closure|embryo_trait:abnormal limb bud morphology
embryo_trait:limbs/digits/tail phenotype(MP)	6	embryo_trait:abnormal limb bud morphology
embryo_trait:cardiovascular system phenotype(MP)	6	embryo_trait:abnormal pericardium morphology
embryo_trait:growth/size/body region phenotype(MP)	4	embryo_trait:abnormal embryo size
embryo_trait:mortality/aging(MP)	4	embryo_trait:abnormal viability by preweaning
embryo_trait:homeostasis/metabolism phenotype(MP)	3	embryo_trait:abnormal fluid regulation
embryo_trait:integument phenotype(MP)	3	embryo_trait:abnormal skin appearance
embryo_trait:skeleton phenotype(MP)	2	embryo_trait:abnormal cranium morphology
embryo_gene:TS20,alimentary system(EMAP)	3	embryo_gene:TS20,oral cavity
embryo_gene:TS20,integumental system(EMAP)	3	embryo_gene:TS20,skin
embryo_gene:TS20,respiratory system(EMAP)	3	embryo_gene:TS20,lung

Supplementary Table 20 List of PPAPs with the maximum number of PPAP-constituting phenotypes belonging to the same biological system as the PPAP query phenotype according to biological system of the query phenotype

Stage_type:top_level_term	Maximum number of phenotypes within the function	Corresponding query phenotype (stage_type:ontology name)
adult_trait:hematopoietic system phenotype(MP)	29	adult_trait:abnormal CD4-positive, alpha beta T cell number
adult_trait:immune system phenotype(MP)	29	adult_trait:abnormal CD4-positive, alpha beta T cell number
adult_trait:behavior/neurological phenotype(MP)	25	adult_trait:abnormal pupillary reflex
adult_trait:craniofacial phenotype(MP)	25	adult_trait:abnormal cranium morphology
adult_trait:limbs/digits/tail phenotype(MP)	25	adult_trait:abnormal femur morphology
adult_trait:skeleton phenotype(MP)	25	adult_trait:abnormal cranium morphology|adult_trait:abnormal femur morphology
adult_trait:vision/eye phenotype(MP)	25	adult_trait:abnormal pupillary reflex
adult_trait:homeostasis/metabolism phenotype(MP)	24	adult_trait:abnormal circulating cholesterol level
adult_trait:growth/size/body region phenotype(MP)	14	adult_trait:abnormal tooth morphology
adult_trait:cardiovascular system phenotype(MP)	11	adult_trait:abnormal retinal vasculature morphology
adult_trait:pigmentation phenotype(MP)	10	adult_trait:abnormal eye pigmentation
adult_trait:integument phenotype(MP)	8	adult_trait:abnormal thermal nociception
adult_trait:nervous system phenotype(MP)	8	adult_trait:abnormal optic disc morphology
adult_trait:digestive/alimentary phenotype(MP)	2	adult_trait:abnormal susceptibility to colitis induced morbidity/mortality
adult_trait:hearing/vestibular/ear phenotype(MP)	2	adult_trait:abnormal auditory brainstem response|adult_trait:abnormal ear morphology
adult_trait:mortality/aging(MP)	2	adult_trait:abnormal susceptibility to colitis induced morbidity/mortality
adult_trait:adipose tissue phenotype(MP)	1	adult_trait:abnormal adipose tissue amount
adult_trait:renal/urinary system phenotype(MP)	1	adult_trait:abnormal kidney weight
adult_trait:reproductive system phenotype(MP)	1	adult_trait:abnormal fertility/fecundity|adult_trait:abnormal external genitalia morphology
adult_trait:reproductive system(MA)	5	adult_trait:testis|adult_trait:uterus|adult_trait:epididymis
adult_trait:cardiovascular system(MA)	1	adult_trait:heart
adult_trait:digestive system(MA)	1	adult_trait:liver|adult_trait:stomach
adult_trait:endocrine system(MA)	1	adult_trait:adrenal gland
adult_trait:immune system(MA)	1	adult_trait:spleen|adult_trait:thymus|adult_trait:lymph node
adult_trait:integumental system(MA)	1	adult_trait:skin
adult_trait:nervous system(MA)	1	adult_trait:brain
adult_trait:respiratory system(MA)	1	adult_trait:lung
adult_trait:sensory organ system(MA)	1	adult_trait:eye
adult_trait:trunk(MA)	1	adult_trait:pancreas
adult_trait:urinary system(MA)	1	adult_trait:kidney|adult_trait:urinary bladder
adult_gene:nervous system(MA)	15	adult_gene:cerebellum
adult_gene:digestive system(MA)	11	adult_gene:small intestine|adult_gene:peyer's patch
adult_gene:hemolymphoid system(MA)	11	adult_gene:peyer's patch
adult_gene:immune system(MA)	11	adult_gene:peyer's patch
adult_gene:respiratory system(MA)	10	adult_gene:oral epithelium
adult_gene:reproductive system(MA)	8	adult_gene:oviduct
adult_gene:exocrine system(MA)	7	adult_gene:prostate gland
adult_gene:sensory organ system(MA)	6	adult_gene:tongue
adult_gene:set of connective tissues(MA)	4	adult_gene:white adipose tissue|adult_gene:brown adipose tissue
adult_gene:cardiovascular system(MA)	3	adult_gene:aorta|adult_gene:heart|adult_gene:blood vessel
adult_gene:endocrine system(MA)	3	adult_gene:thyroid gland|adult_gene:parathyroid gland|adult_gene:adrenal gland
adult_gene:integumental system(MA)	3	adult_gene:mammary gland
adult_gene:musculoskeletal system(MA)	2	adult_gene:skeletal muscle tissue|adult_gene:bone
adult_gene:urinary system(MA)	2	adult_gene:kidney|adult_gene:lower urinary tract
adult_gene:trunk(MA)	1	adult_gene:pancreas
embryo_trait:embryo phenotype(MP)	9	embryo_trait:abnormal neural tube closure
embryo_trait:nervous system phenotype(MP)	9	embryo_trait:abnormal neural tube closure
embryo_trait:growth/size/body region phenotype(MP)	5	embryo_trait:abnormal embryo size
embryo_trait:limbs/digits/tail phenotype(MP)	5	embryo_trait:abnormal limb bud morphology
embryo_trait:craniofacial phenotype(MP)	2	embryo_trait:abnormal craniofacial morphology|embryo_trait:abnormal cranium morphology
embryo_trait:liver/biliary system phenotype(MP)	2	embryo_trait:abnormal liver morphology|embryo_trait:abnormal liver size
embryo_trait:skeleton phenotype(MP)	2	embryo_trait:abnormal cranium morphology
embryo_trait:vision/eye phenotype(MP)	2	embryo_trait:abnormal eye morphology|embryo_trait:abnormal eye size
embryo_trait:cardiovascular system phenotype(MP)	1	embryo_trait:abnormal pericardium morphology|embryo_trait:abnormal heart morphology|embryo_trait:abnormal heartbeat|embryo_trait:abnormal blood circulation
embryo_trait:homeostasis/metabolism phenotype(MP)	1	embryo_trait:abnormal fluid regulation
embryo_trait:integument phenotype(MP)	1	embryo_trait:abnormal skin appearance|embryo_trait:abnormal skin coloration
embryo_trait:mortality/aging(MP)	1	embryo_trait:abnormal viability by preweaning
embryo_gene:TS20,alimentary system(EMAP)	1	embryo_gene:TS20,oral cavity
embryo_gene:TS20,integumental system(EMAP)	1	embryo_gene:TS20,skin
embryo_gene:TS20,respiratory system(EMAP)	1	embryo_gene:TS20,lung

Supplementary Table 21 Sixty relatively rarely occurring phenotypes among 345 PPAP query phenotypes

Stage_type:ontology name	Top level term	Deviation of in-degree/out-degree In-degree	Out-degree	*P* value	*Q* value
adult_gene:peyer's patch(MA)	digestive system|immune system|hemolymphoid system	1	39	6.9E-11	2.55E-09
adult_gene:mammary gland(MA)	exocrine system|integumental system	2	42	1E-10	3.28E-09
adult_gene:brown adipose tissue(MA)	set of connective tissues	8	48	4.3E-08	7.95E-07
adult_gene:white adipose tissue(MA)	set of connective tissues	8	46	1.3E-07	2.03E-06
adult_trait:abnormal circulating phosphate level(MP)	homeostasis/metabolism phenotype	3	32	4E-07	5.5E-06
adult_trait:abnormal mandible morphology(MP)	skeleton phenotype|craniofacial phenotype	0	22	4.6E-07	6.03E-06
adult_gene:lymph node(MA)	immune system	6	38	8.9E-07	1.04E-05
adult_trait:abnormal mean corpuscular hemoglobin concentration(MP)	hematopoietic system phenotype	4	33	1E-06	1.14E-05
adult_trait:abnormal glucose tolerance(MP)	homeostasis/metabolism phenotype	2	27	1.6E-06	1.57E-05
adult_gene:small intestine(MA)	digestive system	12	49	1.8E-06	1.72E-05
adult_trait:abnormal placement of pupils(MP)	vision/eye phenotype	0	20	1.9E-06	1.72E-05
adult_trait:abnormal circulating triglyceride level(MP)	homeostasis/metabolism phenotype	6	36	2.7E-06	2.29E-05
adult_trait:abnormal circulating magnesium level(MP)	homeostasis/metabolism phenotype	4	30	5.9E-06	4.37E-05
adult_trait:abnormal fibula morphology(MP)	limbs/digits/tail phenotype|skeleton phenotype	0	18	7.5E-06	5.17E-05
adult_trait:abnormal hindlimb morphology(MP)	limbs/digits/tail phenotype	0	18	7.5E-06	5.17E-05
adult_trait:abnormal platelet cell number(MP)	hematopoietic system phenotype	4	28	1.9E-05	0.000118
adult_trait:abnormal circulating iron level(MP)	homeostasis/metabolism phenotype	3	25	2.7E-05	0.000151
adult_trait:abnormal scapula morphology(MP)	skeleton phenotype	2	22	3.5E-05	0.000194
adult_gene:thalamus(MA)	nervous system	6	31	4E-05	0.000212
adult_gene:oral epithelium(MA)	respiratory system|digestive system	8	35	4E-05	0.000212
adult_trait:abnormal circulating potassium level(MP)	homeostasis/metabolism phenotype	0	15	6E-05	0.000284
adult_trait:abnormal eye pigmentation(MP)	vision/eye phenotype|pigmentation phenotype	0	15	6E-05	0.000284
adult_trait:abnormal head movements(MP)	behavior/neurological phenotype	0	15	6E-05	0.000284
adult_gene:skeletal muscle tissue(MA)	musculoskeletal system	12	41	7.8E-05	0.000351
adult_gene:stomach(MA)	digestive system	12	41	7.8E-05	0.000351
adult_trait:abnormal head size(MP)	growth/size/body region phenotype	8	33	0.00011	0.000478
adult_trait:abnormal autopod morphology(MP)	limbs/digits/tail phenotype	2	19	0.00022	0.000925
adult_gene:blood vessel(MA)	cardiovascular system	10	35	0.00024	0.000993
adult_trait:abnormal trunk posture (trunk curl)(MP)	behavior/neurological phenotype	10	34	0.00037	0.001508
adult_trait:abnormal circulating glycerol level(MP)	homeostasis/metabolism phenotype	1	15	0.00051	0.001956
adult_trait:abnormal spleen weight(MP)	hematopoietic system phenotype|immune system phenotype	5	24	0.00053	0.002004
adult_trait:abnormal bone structure(MP)	skeleton phenotype	10	33	0.00059	0.002164
adult_trait:abnormal KLRG1+ CD4 alpha beta T cell number(MP)	hematopoietic system phenotype|immune system phenotype	0	11	0.00097	0.003407
adult_gene:uterus(MA)	reproductive system	14	38	0.00115	0.003963
adult_trait:abnormal pelvic girdle bone morphology(MP)	skeleton phenotype	15	39	0.00144	0.004769
adult_trait:abnormal humerus morphology(MP)	limbs/digits/tail phenotype|skeleton phenotype	5	22	0.00149	0.004839
adult_gene:parathyroid gland(MA)	endocrine system	12	34	0.00159	0.005109
adult_trait:ovary(MA)	reproductive system	0	10	0.00194	0.006054
adult_gene:thymus(MA)	immune system	12	33	0.00239	0.007154
adult_gene:liver(MA)	digestive system	14	35	0.0037	0.010491
adult_trait:abnormal circulating glucose level(MP)	homeostasis/metabolism phenotype	3	16	0.00438	0.012117
adult_trait:abnormal circulating amylase level(MP)	homeostasis/metabolism phenotype	6	21	0.00584	0.015962
adult_trait:abnormal femur morphology(MP)	limbs/digits/tail phenotype|skeleton phenotype	11	29	0.00629	0.016787
adult_gene:gall bladder(MA)	digestive system	13	32	0.00646	0.017017
adult_trait:abnormal CD4-positive NK T cell number(MP)	hematopoietic system phenotype|immune system phenotype	4	17	0.00712	0.018547
adult_trait:abnormal circulating creatine kinase level(MP)	homeostasis/metabolism phenotype	0	8	0.00778	0.019806
adult_trait:abnormal forelimb morphology(MP)	limbs/digits/tail phenotype	0	8	0.00778	0.019806
adult_trait:abnormal retinal pigmentation(MP)	vision/eye phenotype|pigmentation phenotype	1	10	0.01166	0.029008
adult_trait:abnormal tail movements(MP)	behavior/neurological phenotype	2	12	0.01286	0.031286
adult_trait:abnormal tail morphology(MP)	limbs/digits/tail phenotype	6	19	0.01447	0.034833
adult_gene:bone(MA)	musculoskeletal system	16	34	0.01502	0.035762
adult_gene:urinary bladder(MA)	urinary system	10	25	0.01643	0.038289
adult_trait:abnormal leukocyte cell number(MP)	hematopoietic system phenotype|immune system phenotype	7	20	0.01895	0.042632
adult_trait:abnormal effector memory CD4-positive, alpha-beta T cell number(MP)	hematopoietic system phenotype|immune system phenotype	4	15	0.01906	0.042632
adult_gene:sublingual gland(MA)	respiratory system|digestive system|exocrine system	1	9	0.0214	0.046442
adult_trait:abnormal response to new environment(MP)	behavior/neurological phenotype	6	18	0.02245	0.046442
adult_trait:abnormal blood uric acid level(MP)	homeostasis/metabolism phenotype	2	11	0.02235	0.046442
adult_trait:abnormal circulating free fatty acids level(MP)	homeostasis/metabolism phenotype	2	11	0.02235	0.046442
adult_trait:kidney(MA)	urinary system	1	9	0.0214	0.046442
adult_trait:abnormal Ly6C-positive mature NK cell number(MP)	hematopoietic system phenotype|immune system phenotype	2	11	0.02235	0.046442

The 60 identified abnormal phenotypes are arranged in ascending order of *Q* value from the top.

Supplementary Table 22 Forty-nine relatively commonly occurring phenotypes among 345 PPAP query phenotypes

Stage_type:ontology name	Top level term	Deviation of in-degree/out-degree In-degree	Out-degree	*P* value	*Q* value
adult_gene:testis(MA)	reproductive system	45	0	5E-14	1.141E-11
adult_gene:heart(MA)	cardiovascular system	48	2	2E-12	1.512E-10
adult_trait:abnormal behavior(MP)	behavior/neurological phenotype	48	2	2E-12	1.512E-10
adult_trait:abnormal rib morphology(MP)	skeleton phenotype	39	0	3E-12	1.859E-10
adult_trait:abnormal body weight(MP)	growth/size/body region phenotype	41	1	2E-11	7.968E-10
adult_trait:abnormal lean body mass(MP)	growth/size/body region phenotype	69	13	2E-10	5.517E-09
adult_gene:spinal cord(MA)	nervous system	45	4	8E-10	1.856E-08
adult_gene:striatum(MA)	nervous system	46	5	2E-09	4.727E-08
adult_trait:abnormal locomotor activation(MP)	behavior/neurological phenotype	47	7	2E-08	4.242E-07
adult_gene:hippocampus(MA)	nervous system	46	8	1E-07	2.026E-06
adult_trait:abnormal gait(MP)	behavior/neurological phenotype	45	8	2E-07	3.245E-06
adult_gene:brainstem(MA)	nervous system	45	9	7E-07	8.347E-06
adult_gene:cerebellum(MA)	nervous system	46	10	1E-06	1.222E-05
adult_trait:abnormal retinal vasculature morphology(MP)	cardiovascular system phenotype|vision/eye phenotype	29	3	2E-06	2.174E-05
adult_gene:cerebral cortex(MA)	nervous system	43	10	5E-06	3.982E-05
adult_gene:olfactory lobe(MA)	nervous system	43	10	5E-06	3.982E-05
adult_gene:brain(MA)	nervous system	43	10	5E-06	3.982E-05
adult_trait:abnormal vertebrae morphology(MP)	skeleton phenotype	35	7	1E-05	9.607E-05
adult_trait:abnormal T cell number(MP)	hematopoietic system phenotype|immune system phenotype	26	3	1E-05	9.607E-05
adult_trait:abnormal eye morphology(MP)	vision/eye phenotype	20	1	2E-05	0.0001228
adult_trait:abnormal effector memory CD8-positive, alpha-beta T cell number(MP)	hematopoietic system phenotype|immune system phenotype	20	1	2E-05	0.0001228
adult_gene:midbrain(MA)	nervous system	34	7	2E-05	0.0001412
adult_gene:hypothalamus(MA)	nervous system	42	12	5E-05	0.0002544
adult_trait:abnormal coat/hair pigmentation(MP)	integument phenotype|pigmentation phenotype	15	0	6E-05	0.0002836
adult_trait:abnormal tibia morphology(MP)	limbs/digits/tail phenotype|skeleton phenotype	39	12	0.0002	0.0008226
adult_trait:abnormal startle reflex(MP)	behavior/neurological phenotype	29	7	0.0003	0.0012436
adult_trait:abnormal circulating alkaline phosphatase level(MP)	homeostasis/metabolism phenotype	47	18	0.0004	0.0015866
adult_trait:abnormal tooth morphology(MP)	growth/size/body region phenotype|craniofacial phenotype	30	8	0.0005	0.0017789
adult_trait:abnormal bone mineralization(MP)	skeleton phenotype	49	20	0.0006	0.0021946
embryo_trait:abnormal craniofacial morphology(MP)	craniofacial phenotype	11	0	0.001	0.0034067
adult_trait:abnormal digit morphology(MP)	limbs/digits/tail phenotype	28	8	0.0012	0.0039632
adult_trait:abnormal mean corpuscular volume(MP)	hematopoietic system phenotype	53	24	0.0012	0.0040262
adult_gene:kidney(MA)	urinary system	40	16	0.0018	0.0056179
adult_trait:abnormal lens morphology(MP)	vision/eye phenotype	23	6	0.0023	0.0069913
adult_trait:abnormal KLRG1-positive NK cell number(MP)	hematopoietic system phenotype|immune system phenotype	15	2	0.0023	0.0070519
adult_trait:abnormal effector memory T-helper cell number(MP)	hematopoietic system phenotype|immune system phenotype	17	3	0.0025	0.0075135
adult_trait:abnormal adipose tissue amount(MP)	adipose tissue phenotype	40	17	0.0031	0.0090188
adult_trait:abnormal retina morphology(MP)	vision/eye phenotype	22	6	0.0037	0.0104909
adult_trait:abnormal memory-marker CD4-negative NK T cell number(MP)	hematopoietic system phenotype|immune system phenotype	20	5	0.004	0.0112643
adult_trait:abnormal body length(MP)	growth/size/body region phenotype	38	17	0.0063	0.0167875
adult_trait:abnormal lumbar vertebrae morphology(MP)	skeleton phenotype	31	13	0.0094	0.0235492
adult_gene:adrenal gland(MA)	endocrine system	36	17	0.0124	0.0304148
adult_trait:immune system phenotype(MP)	immune system phenotype	7	0	0.0156	0.0366969
adult_gene:oviduct(MA)	reproductive system	39	20	0.0179	0.0412887
adult_trait:abnormal eye size(MP)	vision/eye phenotype	15	4	0.0191	0.0426316
adult_trait:abnormal cornea morphology(MP)	vision/eye phenotype	37	19	0.0218	0.0464422
adult_trait:abnormal memory-marker gamma-delta T cell number(MP)	hematopoietic system phenotype|immune system phenotype	11	2	0.0223	0.0464422
adult_gene:trachea(MA)	respiratory system	34	17	0.0236	0.0484452
adult_trait:abnormal B cell number(MP)	hematopoietic system phenotype|immune system phenotype	24	10	0.024	0.0487355

The 49 abnormal phenotypes identified are arranged in ascending order of *Q* value from the top.

Supplementary Data 13

Detailed analyses to derive fundamental characteristics among the 3,686 significant association rules

To deepen understanding of the characteristics of individual relationships among the 3,686 significant association rules, values of more than ten measures, which were attached to each of the 3,686 association rules, were calculated (**Supplementary Table 2**). Here, we provide detailed analysis results for the values of four representative measures, **support**, **confidence**, **rule polarity**, **rule significance**. First, to aid understanding of the analysis results, we outline the biological meaning of each measure, as follows (refer to Methods for details):

- **Support**: Co-expression frequency of the two abnormal phenotypes constituting each association rule.
- **Confidence**: In an association rule between phenotypes, when the phenotype on the left-hand side (premise) is abnormal, the probability that the phenotype on the right-hand side (conclusion) is abnormal. Generally, this measure represents the strength of the rule.
- **Rule polarity**: A measure representing the magnitude of the difference between the two confidence values in a bidirectional rule. Considering the formula for calculating the confidence value, the larger the value of this measure, the larger the difference between the number of abnormal cases in the premise and those in the conclusion of an association rule. Therefore, this measure indicates the relative degree of difference between the number of abnormal cases in the premise and those in the conclusion of an association rule. In this study, because an association rule that had a larger confidence value in a bidirectional rule was selected as one of the 3,686 significant rules, a larger value of this measure represents a relatively smaller number of abnormal cases in the premise.
- **Rule significance**: A measure demonstrating the statistical significance of an association rule between phenotypes. The larger the value of this measure, the more reliable the association rule.

We performed various analyses with the following objectives:

1. How are values of the four measures distributed?
2. How are values of the four measures correlated?
3. Which ‘stage/type’ rule categories are enriched among the upper and lower values for each of the four measures?
4. What are the distributions of values of the four measures for each of the eight ‘stage/type’ rule categories constituting the 3,686 significant rules?
5. Are there any differences in the distributions of values of the four measures between ‘stage/type’ rule categories?
6. For each of the four measures, in which comparisons between ‘stage/type’ rule categories can we observe differences?
7. For values of the four measures, what kinds of rules between biological systems show outstanding higher and

lower values, according to the five between-‘stage/type’ rule categories?

Note that Supplementary Data 13 (SD 13) includes SD 13 Figs. 1–5 at the end of the text.

1. How are values of the four measures distributed?

Refer to **Supplementary Fig. 2a–d.** The mean support value was 2.4%, and the median was 1% (**Supplementary Fig. 2a**). The mean confidence value was 48.2%, the median was 42.9%, and the mode was 18% (**Supplementary Fig. 2b**). For rule polarity values, the mean was 0.58 and the median was 0.32 (**Supplementary Fig. 2c**). For rule significance values, the mean was 4.25 and the median was 2.86 (**Supplementary Fig. 2d**). Characteristics of the distributions of values of these four measures by stage/type and biological system rule categories are shown in **Supplementary Fig. 2f–j** and **SD 13 Figs. 1–5**, respectively.

2. How are values of the four measures correlated?

Refer to **Supplementary Fig. 2e**. Spearman’s rank correlation coefficients were calculated to evaluate correlations between the four measures described above (**Supplementary Fig. 2e**). There were strong positive correlations between three pairs of measures: support and confidence, support and rule significance, confidence and rule significance (r = 0.79, 0.52, 0.44, respectively). The probabilities of type I error were practically nil for all the three pairwise comparisons. These results indicate interdependent relationships among these three measures.

3. Which ‘stage/type’ rule categories are enriched among the upper and lower values for each of the four measures?

Refer to **Supplementary Table 7**. To determine which stage/type rule categories were enriched among the upper and lower values for each of the four measures, we performed enrichment analyses (**Supplementary Table 7**). Both between-‘adult/gene’ and between-‘embryo/gene’ rules were positively enriched among rules with higher support values (Bonferroni-corrected P = 7.1×10^{-15}, 0.0013, respectively), and between-‘adult/trait’ rules were positively enriched among rules with lower support values (Bonferroni-corrected P = 1.9×10^{-6}). For rules with higher confidence values, both between-‘adult/gene’ and between-‘embryo/gene’ rules were positively enriched, as for higher support values (Bonferroni-corrected P = 8.6×10^{-6}, 0.0013, respectively); further, between-‘embryo/trait’ and ‘adult/gene => adult/trait’ rules were also positively enriched (Bonferroni-corrected P = 0.00016, 0.044, respectively). In addition, between-‘adult/trait’ rules were positively enriched among rules with lower confidence values (Bonferroni-corrected P = 1.9×10^{-6}), as for lower support values. For rules with higher rule polarity values, only between-‘adult/gene’ rules were positively enriched (Bonferroni-corrected P = 1.5×10^{-9}), and for those with lower rule polarity values, three kinds of between-‘stage/type’ rules (between-‘embryo/trait’, ‘adult/gene => adult/trait’, and ‘adult/trait => adult/gene’) were positively enriched (Bonferroni-corrected P = 0.0083, 0.0095, and 0.047, respectively). Only between-‘adult/gene’ rules were positively enriched among those with higher significance values (Bonferroni-corrected P = 1.7×10^{-14}). These results are summarized in **Fig. 3a**, and we clarified the selective enrichment by between-‘stage/type’ rules in the upper/lower parts of values for these four measures for the 3,686 significant rules (refer to main text for details).

4. **What are the distributions of values of the four measures for each of the eight 'stage/type' rule categories constituting the 3,686 significant rules?**

Refer to **Supplementary Fig. 2f–i**. **Supplementary Fig. 2f–i**, respectively, shows distributions of values for each of the four measures (support, confidence, rule polarity, rule significance) according to eight 'stage/type' rule categories, constituting the 3,686 significant association rules. Summary statistics for these four measures according to the eight between-'stage/type' rules are summarized in the 'basic_statistics' sheet in **Supplementary Table 8**.

5. **Are there any differences in the distributions of values of the four measures between 'stage/type' rule categories?**

Refer to the 'anova_table' sheet in **Supplementary Table 8**. To examine whether there were differences in distributions of values of the four measures between the eight 'stage/type' rule categories, we performed one-way ANOVA. We found significant differences between the 'stage/type' rule categories in all of the four measures (P = 0, 0, 8.1×10^{-44}, 1.7×10^{-169}, respectively).

6. **For each of the four measures, in which comparisons between 'stage/type' rule categories can we observe differences?**

Refer to **Supplementary Fig. 2j**. This figure summarizes the results of the post-hoc test (Games-Howell method, refer to the 'posthoc' sheet in **Supplementary Table 8**) that was performed after the ANOVA described above. That is, P values from the post-hoc test of values in each of the four measures are presented for all ten comparisons between 'stage/type' rule categories, which comprised five 'stage/type' rule categories, excluding those categories with small sample sizes ($n \leq 6$) (**Supplementary Fig. 2j**). In the comparison 'adult_trait => adult_trait: adult_gene => adult_gene', which covered 92.9% (3,424) of all the significant rules (3,686), we found remarkably significant differences for all four measures (P = 0, 0, 5.4×10^{-11}, and 2.2×10^{-10}, respectively). This result indicates that, in comparisons of rules between trait expressions and rules between gene expressions at the adult stage, there are marked differences in the values of all four measures. Also, significant differences were observed in all four measures at 80% or more for all ten comparisons between 'stage/type' rule categories (at the 0.05 significance level). Therefore, the following analyses of the four measures for extracting features of rules between biological systems were performed according to the five types of between-'stage/type' rule categories.

7. **For values of the four measures, what kinds of rules between biological systems show outstanding higher and lower values, according to the five between-'stage/type' rule categories?**

For values of the four measures, characteristic rules between biological systems were explored by five between-'stage/type' rules: 'adult_trait => adult_trait', 'adult_gene => adult_gene', 'adult_trait => adult_gene', 'embryo_trait => embryo_trait', and 'adult_gene => embryo_trait'. The five pairs of results in **Supplementary Tables 9–13** and **SD 13 Figs. 1–5** correspond to analyses by theses five types of between-'stage/type' rules,

respectively. To outline the analyses, we first determined the distributions of values in each of the four measures for each between-‘biological systems’ rule, according to the five types of between-‘stage/type’ rules. Subsequently, we performed one-way ANOVAs and post-hoc tests for values in each of the four measures, according to rules between biological systems for only two types of between-‘stage/type’ rule (‘adult_trait => adult_trait’ and ‘adult_gene => adult_gene’), where the number of between-‘biological systems’ rules with seven or more ‘between-phenotypes’ rules was sufficient for feature extraction (i.e., $n \geq 7$). By hierarchical clustering using the *P* values resulting from the post-hoc tests, we finally identified relationships (rules) between biological systems with outstanding high/low values at each measure. Information on what kinds of ‘between-phenotypes’ rules exist in each between-‘biological systems’ rule can be obtained by applying the filter function to the columns ‘lhs_stage_type: top level term’ and ‘rhs_stage_type: top level term’ in **Supplementary Table 2**. In the following sections, we present detailed results of feature analyses of values in each of the four measures for between-‘biological systems’ rules, according to each of the five types of between-‘stage/type’ rules.

7.1. According to between-‘biological systems’ rules of the between-‘stage/type’ rule ‘adult_trait => adult_trait’

Refer to **Supplementary Table 9** and **SD 13 Fig. 1**. The between-‘stage/type’ rule ‘adult_trait => adult_trait’ consisted of 244 distinct between-‘biological systems’ rules (**Fig. 2d-i**), of which 86 had seven or more ‘between-phenotypes’ rules. We analyzed the 86 rules to extract features of the values of the four measures (support, confidence, rule polarity, rule significance) in ‘between-phenotypes’ rules (**SD 13 Fig. 1a–c, 1d–f, 1g–i, 1j–l**, respectively). For each of the four measures, the results of analysis of basic statistics, Levine’s test for equality of variance, ANOVA, post-hoc test (Games-Howell method), and hierarchical clustering according to the *P* values resulting from the post-hoc test are presented in **Supplementary Table 9**. By hierarchical clustering using *P* values from the post-hoc tests, we specified between-‘biological systems’ rules with remarkably high/low mean values for each of support, confidence, rule polarity, and rule significance (**SD 13 Fig. 1c**, **f**, **i**, **l**, respectively). For the between-‘stage/type’ rule ‘adult_trait => adult_trait’, we finally identified 20 distinct characteristic rules between biological systems, which are summarized in the upper table of **Fig. 3b**. Below, we present representative examples from the 20 distinct rules between biological systems.

- Example 1: Characteristic rules between biological systems in the ‘adult_trait => adult_trait’ category

Label (enrichment rank)	Rules between biological systems			Num. of rules between phenotypes
	Lhs_stage_type : top level term	Direction	Rhs_stage_type : top level term	
2	adult_trait:hematopoietic system phenotype(MP)	⇒	adult_trait:hematopoietic system phenotype(MP)	386
3	adult_trait:hematopoietic system phenotype(MP)	⇒	adult_trait:immune system phenotype(MP)	354
4	adult_trait:immune system phenotype(MP)	⇒	adult_trait:immune system phenotype(MP)	340
5	adult_trait:immune system phenotype(MP)	⇒	adult_trait:hematopoietic system phenotype(MP)	343

The above four between-‘biological systems’ rules comprised only two kinds of biological system, ‘adult_trait:hematopoietic system phenotype(MP)’ and ‘adult_trait:immune system phenotype(MP)’, and exhibited much higher mean values for support, confidence, rule polarity, and rule significance for ‘between-phenotypes’ rules belonging to each of the four between-‘biological systems’ rules (**SD 13 Fig. 1c, f, i, l,** respectively). That is, we detected remarkable features, indicating that between-‘abnormal phenotypes’ rules belonging to each of the

above four between-'biological systems' rules exhibited greater co-expression frequencies in rules comprising two phenotypes, greater strength in 'between-phenotypes' rules, greater difference in the number of abnormal cases between two phenotypes constituting a rule, and greater statistical significance for 'between-phenotypes' rules. Also, because all ontology-annotated phenotypes based on parameters measured by the immunophenotyping (FACS) test were classified into the above two biological systems ('adult_trait:hematopoietic system phenotype(MP)' and 'adult_trait:immune system phenotype(MP)'; refer to **Supplementary Table 1**), almost all of the rules between abnormal phenotypes belonging to each of the above four between-'biological systems' rules were, in fact, derived from relationships among the parameters measured in the FACS test. In addition, note that 'between-phenotypes' rules belonging to each of the above four between-'biological systems' rules were positively selected with statistical significance during the extraction of the 3,686 significant rules (refer to **Fig. 2d** and **Supplementary Table 6**).

- Example 2: Characteristic rules between biological systems in the 'adult_trait => adult_trait' category

Label (enrichment rank)	Rules between biological systems			Num. of rules between phenotypes
	Lhs_stage_type : top level term	Direction	Rhs_stage_type : top level term	
38	adult_trait:skeleton phenotype(MP)	⇒	adult_trait:vision/eye phenotype(MP)	54
85	adult_trait:limbs/digits/tail phenotype(MP)	⇒	adult_trait:craniofacial phenotype(MP)	16
93	adult_trait:behavior/neurological phenotype(MP)	⇒	adult_trait:skeleton phenotype(MP)	39

The above three between-'biological systems' rules were identified as exhibiting lower mean values for confidence in 'between-phenotypes' rules belonging to each of the three between-'biological systems' rules (18.7%, 17.4%, and 19.6%, respectively; **SD 13 Fig. 1f**). That is, we detected remarkable features, indicating that the rules between abnormal phenotypes belonging to each of the above three between-'biological systems' rules exhibited lower values for the strength of rules between phenotypes. In addition, the between-'biological systems' rule, label 85, exhibited extremely low mean values for support (0.2%) in rules between phenotypes belonging to the label 85 rule, enabling us to detect a low-frequency between-'biological systems' rule, which would be difficult to find as a significant relationship without using the rule selection criteria and data from comprehensive phenotyping analyses, both of which were applied in this study. Further, note that 'between-phenotypes' rules belonging to each of the above three between-'biological systems' rules were positively selected with statistical significance during extraction of the 3,686 significant rules (refer to **Fig. 2d** and **Supplementary Table 6**).

- Example 3: A characteristic rule between biological systems in the 'adult_trait => adult_trait' category

Label (enrichment rank)	Rules between biological systems			Num. of rules between phenotypes
	Lhs_stage_type : top level term	Direction	Rhs_stage_type : top level term	
28	adult_trait:homeostasis/metabolism phenotype(MP)	⇒	adult_trait:growth/size/body region phenotype(MP)	50

The above between-'biological systems' rule was identified as exhibiting the highest mean value for rule polarity (0.8; **SD 13 Fig. 1i**) among 'between-phenotypes' rules belonging to this between-'biological systems' rule. That is, we detected a feature that, in 'between-phenotypes' rules belonging to this between-'biological systems' rule, the relative difference in the number of abnormal cases for each of the 'between-phenotypes' rules was remarkably large. This feature indicates that abnormal phenotypes belonging to the biological system 'adult_trait:homeostasis/metabolism phenotype(MP)' exhibit a relatively small number of abnormal phenotypic expression cases, while abnormal phenotypes in the biological system 'adult_trait:growth/size/body region

phenotype(MP)' exhibit a relatively large number of cases of abnormal phenotypic expression. Note that 'between-phenotypes' rules belonging to this between-'biological systems' rule were positively selected with statistical significance during extraction of the 3,686 significant rules (**Fig. 2d** and **Supplementary Table 6**).

- Example 4: Characteristic rules between biological systems in the 'adult_trait => adult_trait' category

Label (enrichment rank)	Rules between biological systems			Num. of rules between phenotypes
	Lhs_stage_type : top level term	Direction	Rhs_stage_type : top level term	
8	adult_trait:homeostasis/metabolism phenotype(MP)	⇒	adult_trait:homeostasis/metabolism phenotype(MP)	151
204	adult_trait:behavior/neurological phenotype(MP)	⇒	adult_trait:craniofacial phenotype(MP)	14
327	adult_trait:behavior/neurological phenotype(MP)	⇒	adult_trait:homeostasis/metabolism phenotype(MP)	27
406	adult_trait:integument phenotype(MP)	⇒	adult_trait:skeleton phenotype(MP)	7

The above four between-'biological systems' rules were identified as exhibiting extremely small mean values of rule polarity for 'between-phenotypes' rules belonging to each of these four between-'biological systems' rules (**SD 13 Fig. 1i**). That is, we detected the feature that, in 'between-phenotypes' rules belonging to each of these four between-'biological systems' rules, the relative difference in the number of abnormal cases for each 'between-phenotypes' rule was remarkably small. This feature demonstrates that, in these four between-'biological systems' rules, there are a large number of 'between-phenotypes' rules, each of which exhibits a similar number of abnormal cases in the two abnormal phenotypes constituting the rule. In addition, note that 'between-phenotypes' rules for the between-'biological systems' rule 'adult_trait:homeostasis/metabolism phenotype(MP) => adult_trait:homeostasis/metabolism phenotype(MP)' (label 8), in which abnormal phenotypes were mainly derived from parameters measured in clinical blood chemistry tests, were positively selected with statistical significance during extraction of the 3,686 significant rules (**Fig. 2d** and **Supplementary Table 6**).

- Example 5: A characteristic rule between biological systems in the 'adult_trait => adult_trait' category

Label (enrichment rank)	Rules between biological systems			Num. of rules between phenotypes
	Lhs_stage_type : top level term	Direction	Rhs_stage_type : top level term	
1	adult_trait:skeleton phenotype(MP)	⇒	adult_trait:skeleton phenotype(MP)	181

The above between-'biological systems' rule was identified as exhibiting the highest mean value for the measure significance (4.7, **SD 13 Fig. 1l**) among 'between-phenotypes' rules belonging to this between-'biological systems' rule. That is, we detected the feature that 'between-phenotypes' rules belonging to this between-'biological systems' rule exhibited higher significance. In addition to this between-'biological systems' rule, the four between-'biological systems' rules presented in example 1 have the same feature. Further, note that 'between-phenotypes' rules belonging to this between-'biological systems' rule were mostly positively selected with statistical significance during extraction of the 3,686 significant rules (**Fig. 2d** and **Supplementary Table 6**).

For further details of the mean values for each of the four measures for between-'biological systems' rules belonging to the between-'stage/type' rule 'adult_trait => adult_trait', refer to the 'heatmap' sheets for each measure in **Supplementary Table 9**.

7.2. According to between-'biological systems' rules belonging to the between-'stage/type' rule 'adult_gene => adult_gene'

Refer to **Supplementary Table 10** and **SD 13 Fig. 3**. In this study, all of the phenotypes annotated with the phenotypic category 'adult_gene' were derived from the parameters measured by the adult LacZ test. Therefore, note that between-'biological systems' rules belonging to the between-'stage/type' rule 'adult_gene => adult_gene' correspond to rules between MA ontology-annotated tissues, where abnormal gene expression was observed. The between-'stage/type' rule 'adult_gene => adult_gene' consists of 200 distinct between-'biological systems' rules (**Fig. 2d-i**), among which, 56 had seven or more 'between-phenotypes' rules. Here, we analyzed these 56 rules to extract features of the values of the four measures (support, confidence, rule polarity, rule significance) of rules between phenotypes (**SD 13 Fig. 2a–c, 2d–f, 2g–i, and 2j–l**, respectively). For each of the four measures, the results of basic statistical analyses, Levine's test for equality of variance, ANOVA, post-hoc test (Games-Howell method), and hierarchical clustering using the *P* values resulting from the post-hoc test are detailed in **Supplementary Table 10**. By hierarchical clustering using *P* values from these post-hoc tests, we specified between-'biological systems' rules with remarkably high/low mean values for support, confidence, rule polarity, and rule significance (**SD 13 Fig. 2c, f, i, and l**, respectively). In the between-'stage/type' rule 'adult_gene => adult_gene', we finally identified 30 distinct characteristic rules between biological systems, which are summarized in the table in the lower part of **Fig. 3b**. Below, we present representative examples from the 30 distinct rules between biological systems.

- Example 1: A characteristic rule between biological systems in the 'adult_gene => adult_gene' category

Label (enrichment rank)	Rules between biological systems			Num. of rules between phenotypes
	Lhs_stage_type : top level term	Direction	Rhs_stage_type : top level term	
7	adult_gene:nervous system(MA)	⇒	adult_gene:nervous system(MA)	90

The above between-'biological systems' rule, comprising a single biological system, 'adult_gene:nervous system(MA)', exhibited extremely high mean values for support (8.7%, **SD 13 Fig. 2c**), confidence (89%, **SD 13 Fig. 2f**), and rule significance (12, **SD 13 Fig. 2l**), among 'between-phenotypes' rules belonging to this between-'biological systems' rule. That is, we detected remarkable features, indicating that the between-'abnormal phenotypes' rules belonging to this between-'biological systems' rule exhibited a greater co-expression frequency in two phenotypes constituting rules, greater strength in 'between-phenotypes' rules, and greater statistical significance in 'between-phenotypes' rules. In addition, note that 'between-phenotypes' rules belonging to this between-'biological systems' rule were positively selected with statistical significance during extraction of the 3,686 significant rules (**Fig. 2d** and **Supplementary Table 6**).

- Example 2: A characteristic rule between biological systems in the 'adult_gene => adult_gene' category

Label (enrichment rank)	Rules between biological systems			Num. of rules between phenotypes
	Lhs_stage_type : top level term	Direction	Rhs_stage_type : top level term	
10	adult_gene:immune system(MA)	⇒	adult_gene:nervous system(MA)	43

The above between-'biological systems' rule exhibited an extremely low mean value for support (2.3%, **SD 13 Fig. 2c**) while having extremely high mean values for both confidence (85%, **SD 13 Fig. 2f**) and polarity (1.5, **SD 13 Fig. 2i**), for 'between-phenotypes' rules belonging to this between-'biological systems' rule. That is, we detected

remarkable features, indicating that between-'abnormal phenotypes' rules belonging to this between-'biological systems' rule exhibited lower co-expression frequency in two phenotypes constituting rules, while exhibiting both greater strength of 'between-phenotypes' rules and greater difference in the number of abnormal cases in each 'between-phenotypes' rule. In addition, note that 'between-phenotypes' rules belonging to this between-'biological systems' rule were positively selected with statistical significance during extraction of the 3,686 significant rules (**Fig. 2d** and **Supplementary Table 6**).

- Example 3: A characteristic rule between biological systems in the 'adult_gene => adult_gene' category

Label (enrichment rank)	Rules between biological systems			Num. of rules between phenotypes
	Lhs_stage_type : top level term	Direction	Rhs_stage_type : top level term	
45	adult_gene:hemolymphoid system(MA)	⇒	adult_gene:nervous system(MA)	11

The above between-'biological systems' rule exhibited extremely low mean values for both support (1.4%, **SD 13 Fig. 2c**) and rule significance (2.8, **SD 13 Fig. 2l**), while exhibiting extremely high mean values for both confidence (86%, **SD 13 Fig. 2f**) and rule polarity (2.3, **SD 13 Fig. 2i**), for 'between-phenotypes' rules belonging to this between-'biological systems' rule. That is, we detected remarkable features, indicating that between-'abnormal phenotypes' rules belonging to this between-'biological systems' rule exhibited both lower co-expression frequencies in the two phenotypes constituting rules and lower statistical significance for 'between-phenotypes' rules, while exhibiting both greater strength of 'between-phenotypes' rules and a greater difference in the number of abnormal cases in each 'between-phenotypes' rule. In addition, note that 'between-phenotypes' rules belonging to this between-'biological systems' rule were positively selected with statistical significance during extraction of the 3,686 significant rules (**Fig. 2d** and **Supplementary Table 6**).

For further details of the mean values for each of the four measures of between-'biological systems' rules belonging to the between-'stage/type' rule 'adult_gene => adult_gene', refer to the 'heatmap' sheets for each measure in **Supplementary Table 10**.

7.3. According to between-'biological systems' rules belonging to the between-'stage/type' rule 'adult_trait => adult_gene'

Refer to **Supplementary Table 11** and **SD 13 Fig. 3**. The between-'stage/type' rule 'adult_trait => adult_gene' comprised 112 'between-phenotypes' rules covering 69 distinct between-'biological systems' rules (right table, **Fig. 2d-i**). Of the 69 between-'biological systems' rules, there were only three rules that had seven or more 'between-phenotypes' rules (table below). Therefore, in this study we present only distributions of values in the four measures (support, confidence, rule polarity, rule significance) for each 'between-phenotypes' rule (**SD 13 Fig. 3a–d**, respectively) and their fundamental statistics (**Supplementary Table 11**), according to the 69 distinct between-'biological systems' rules. Furthermore, note that 'between-phenotypes' rules belonging to each of the three above-described between-'biological systems' rules were not positively/negatively selected with statistical significance during extraction of the 3,686 significant rules (refer to **Fig. 2d** and **Supplementary Table 6**).

Label (enrichment rank)	Rules between biological systems			Num. of rules between phenotypes
	Lhs_stage_type : top level term	Direction	Rhs_stage_type : top level term	
283	adult_trait:hematopoietic system phenotype(MP)	⇒	adult_gene:nervous system(MA)	26
508	adult_trait:immune system phenotype(MP)	⇒	adult_gene:nervous system(MA)	15
579	adult_trait:hematopoietic system phenotype(MP)	⇒	adult_gene:digestive system(MA)	7

7.4. According to between-'biological systems' rules belonging to the between-'stage/type' rule 'embryo_trait => embryo_trait'

Refer to **Supplementary Table 12** and **SD 13 Fig. 4**. The between-'stage/type' rule 'embryo_trait => embryo_trait' comprised 86 'between-phenotypes' rules covering 45 distinct between-'biological systems' rules (right table, **Fig. 2d-i**). Of the 45 between-'biological systems' rules, only four had seven or more 'between-phenotypes' rules (refer to the table below). Therefore, in this study we present only distributions of values of the four measures (support, confidence, rule polarity, rule significance) for each 'between-phenotypes' rule (**SD 13 Fig. 4a–d**, respectively), and their fundamental statistics (**Supplementary Table 12**), according to the 45 distinct between-'biological systems' rules. In addition, note that 'between-phenotypes' rules belonging to each of the two between-'biological systems' rules, 'embryo_trait:nervous system phenotype(MP) => embryo_trait:nervous system phenotype(MP)' (label 48) and 'embryo_trait:embryo phenotype(MP) => embryo_trait:nervous system phenotype(MP)' (label 187), were positively selected with statistical significance during extraction of the 3,686 significant rules (**Fig. 2d** and **Supplementary Table 6**).

Label (enrichment rank)	Rules between biological systems			Num. of rules between phenotypes
	Lhs_stage_type : top level term	Direction	Rhs_stage_type : top level term	
48	embryo_trait:nervous system phenotype(MP)	⇒	embryo_trait:nervous system phenotype(MP)	16
187	embryo_trait:embryo phenotype(MP)	⇒	embryo_trait:nervous system phenotype(MP)	8
216	embryo_trait:nervous system phenotype(MP)	⇒	embryo_trait:embryo phenotype(MP)	7
244	embryo_trait:embryo phenotype(MP)	⇒	embryo_trait:embryo phenotype(MP)	10

7.5. According to the between-'biological systems' rules belonging to the between-'stage/type' rule 'adult_gene => adult_trait'

Refer to **Supplementary Table 13** and **SD 13 Fig. 5**. The between-'stage/type' rule 'adult_gene => adult_trait' comprised 55 'between-phenotypes' rules covering 42 distinct between-'biological systems' rules (right table, **Fig. 2d-i**). Of the 42 between-'biological systems' rules, only two had seven or more 'between-phenotypes' rules (refer to the table below). Therefore, in this study we present only distributions of values for the four measures (support, confidence, rule polarity, rule significance) for each 'between-phenotypes' rule (**SD 13 Fig. 5a–d**, respectively), and their fundamental statistics (**Supplementary Table 13**), according to the 42 distinct between-'biological systems' rules. Furthermore, note that 'between-phenotypes' rules belonging to each of the two above-described between-'biological systems' rules were negatively selected with statistical significance during extraction of the 3,686 significant rules (refer to **Fig. 2d** and **Supplementary Table 6**).

Label (enrichment rank)	Rules between biological systems			Num. of rules between phenotypes
	Lhs_stage_type : top level term	Direction	Rhs_stage_type : top level term	
578	adult_gene:digestive system(MA)	⇒	adult_trait:hematopoietic system phenotype(MP)	7
581	adult_gene:nervous system(MA)	⇒	adult_trait:hematopoietic system phenotype(MP)	7

SD 13 Fig. 1a–c

a

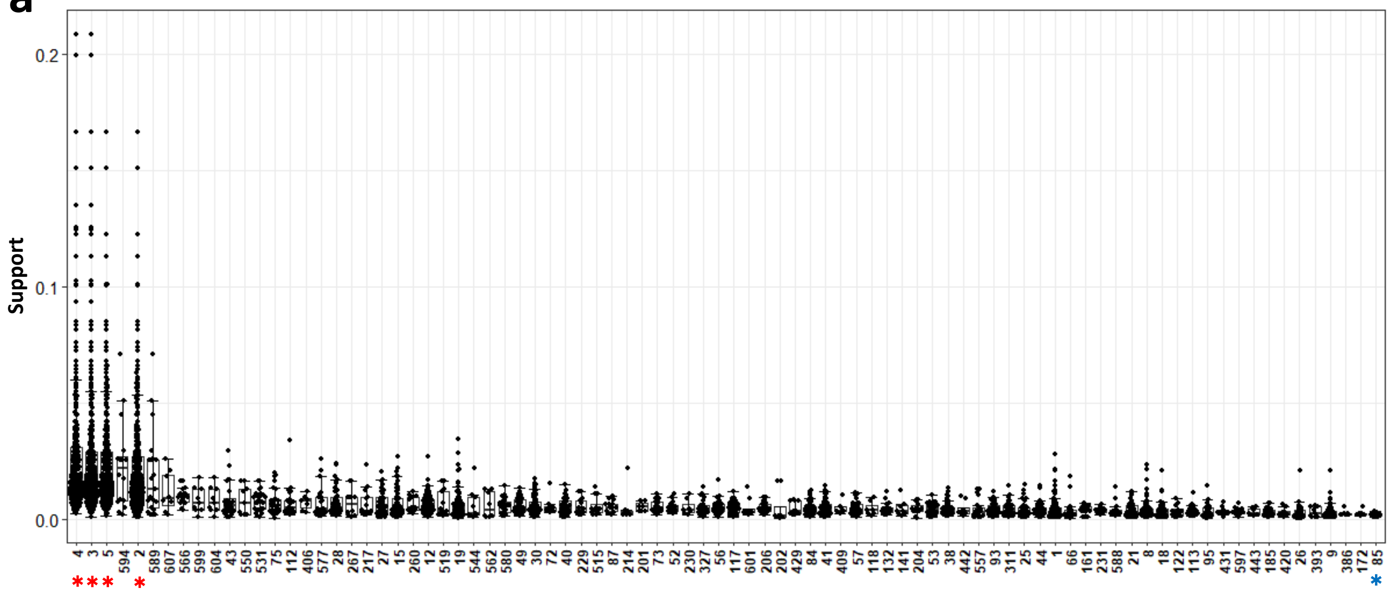

b

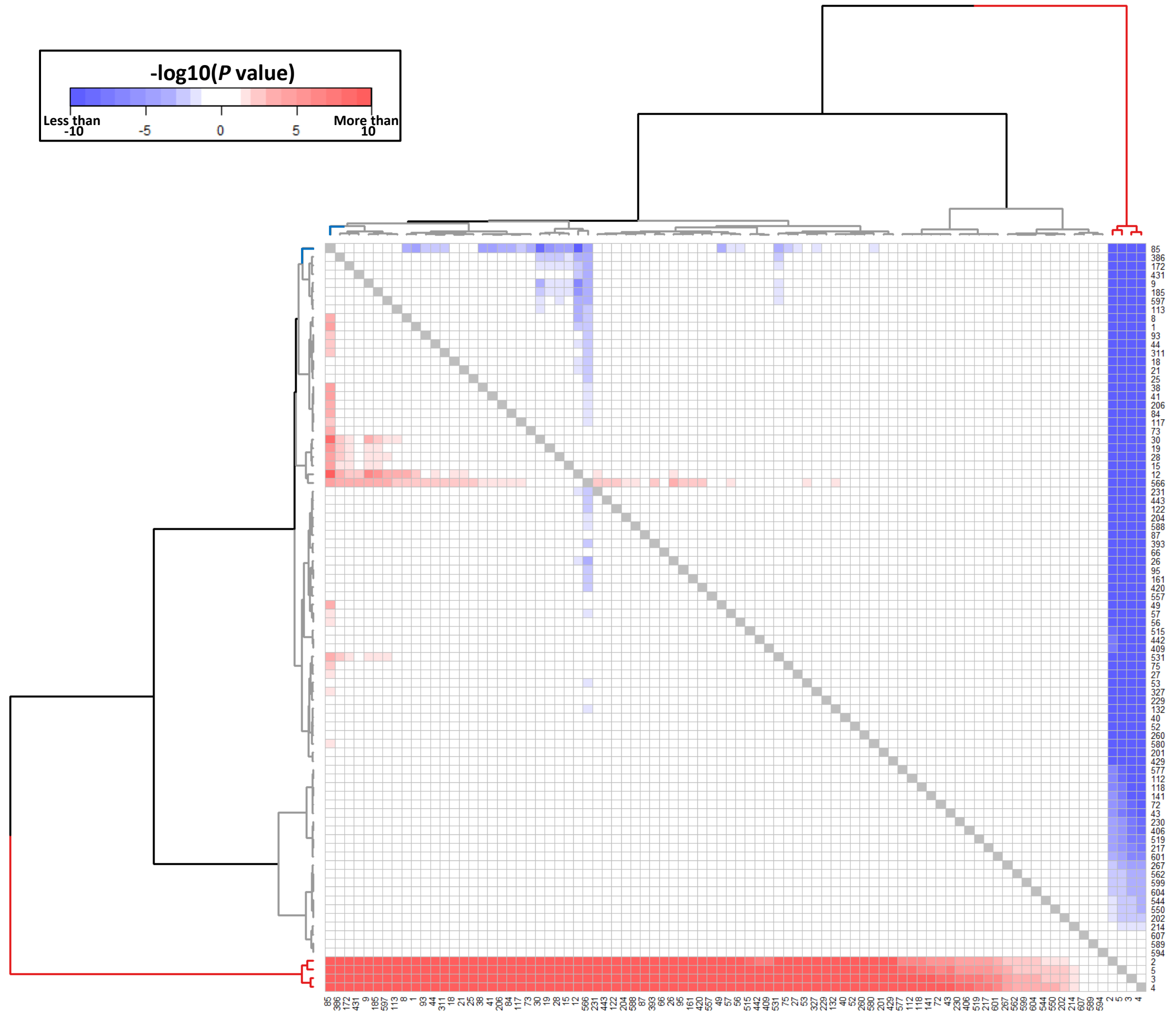

c

Label (enrichment rank)	Rules between biological systems: Lhs_stage_type : top level term	Direction	Rhs_stage_type : top level term	Num. of rules between phenotypes	Mean (support)	Statistics in post hoc test: Num. of significant differences ($P < 0.01$)	Accumulated -log10(P value)
4	adult_trait:immune system phenotype(MP)	⇒	adult_trait:immune system phenotype(MP)	340	0.0274	78	978.4
3	adult_trait:hematopoietic system phenotype(MP)	⇒	adult_trait:immune system phenotype(MP)	354	0.0266	78	969.2
5	adult_trait:immune system phenotype(MP)	⇒	adult_trait:hematopoietic system phenotype(MP)	343	0.0245	78	902.1
2	adult_trait:hematopoietic system phenotype(MP)	⇒	adult_trait:hematopoietic system phenotype(MP)	386	0.0224	75	875.3
85	adult_trait:limbs/digits/tail phenotype(MP)	⇒	adult_trait:craniofacial phenotype(MP)	16	0.0021	24	-171.7

SD 13 Fig. 1d–f

d

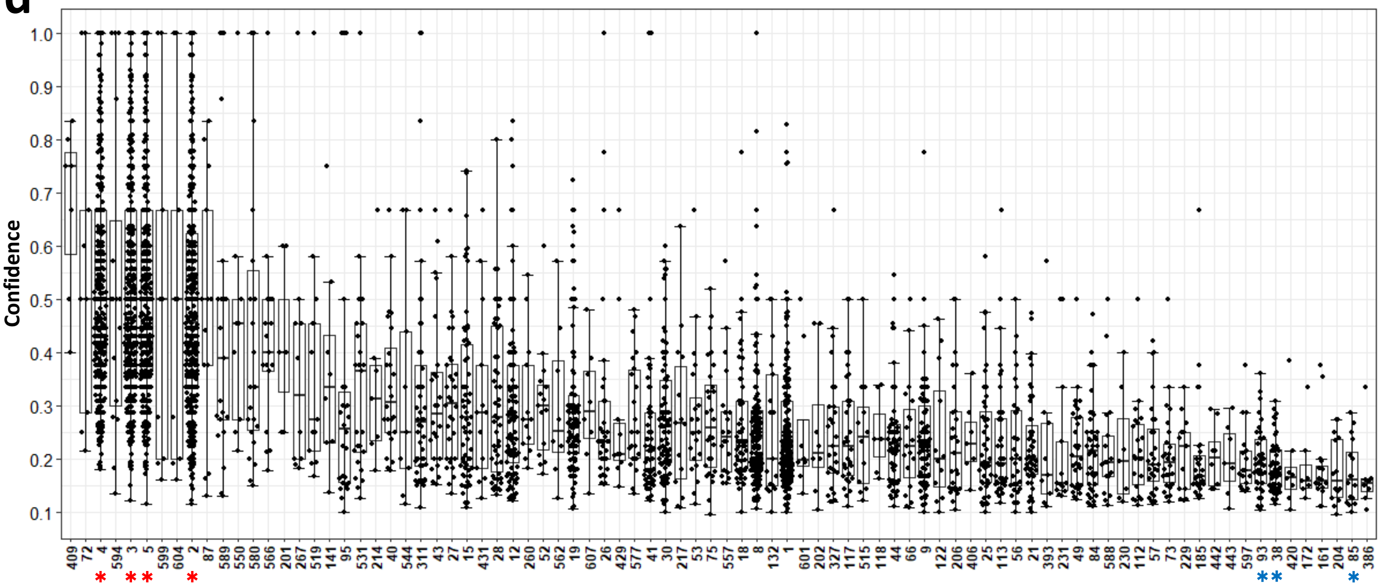

e

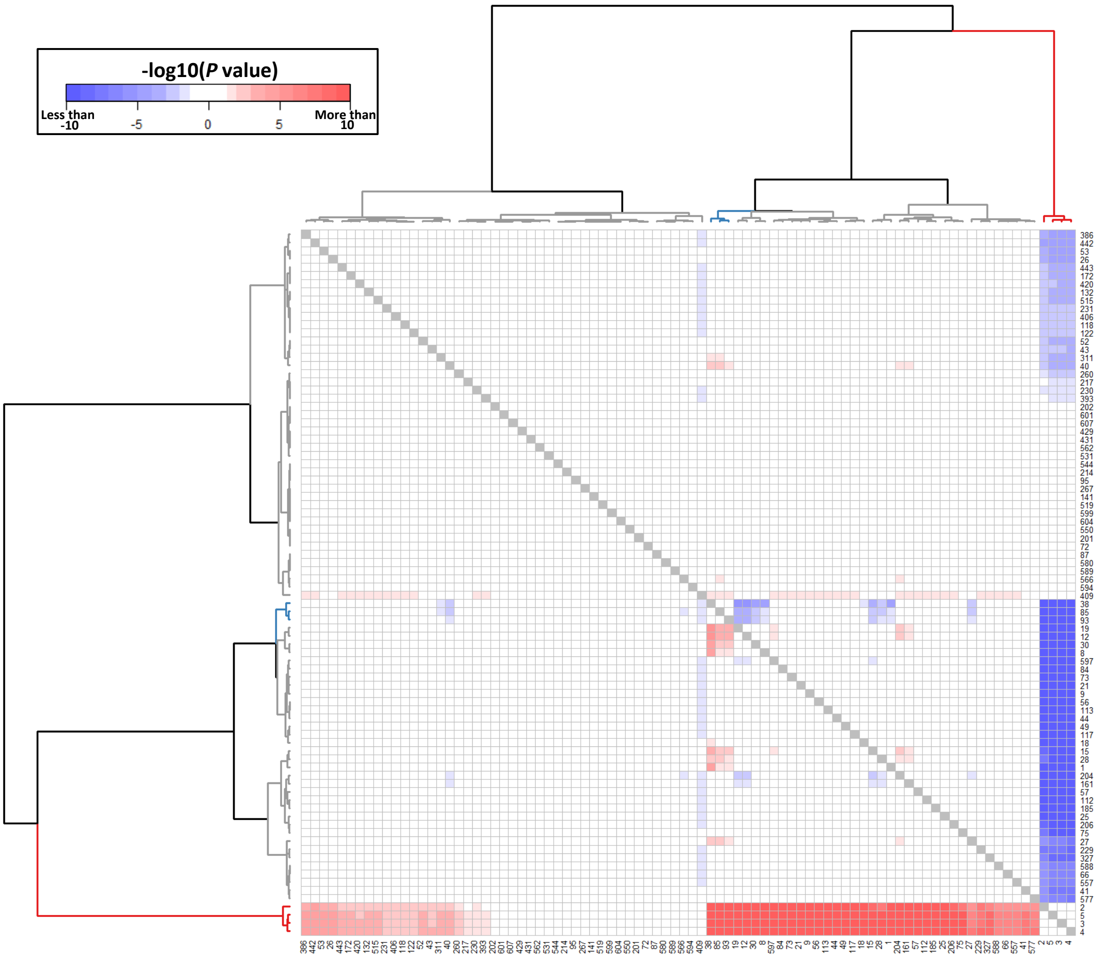

f

Label (enrichment rank)	Rules between biological systems: Lhs_stage_type : top level term	Direction	Rhs_stage_type : top level term	Num. of rules between phenotypes	Mean (confidence)	Statistics in post hoc test: Num. of significant differences ($P < 0.01$)	Accumulated -log10(P value)
4	adult_trait:immune system phenotype(MP)	⇒	adult_trait:immune system phenotype(MP)	340	0.532	55	512.7
3	adult_trait:hematopoietic system phenotype(MP)	⇒	adult_trait:immune system phenotype(MP)	354	0.523	55	508.5
5	adult_trait:immune system phenotype(MP)	⇒	adult_trait:hematopoietic system phenotype(MP)	343	0.521	55	496.7
2	adult_trait:hematopoietic system phenotype(MP)	⇒	adult_trait:hematopoietic system phenotype(MP)	386	0.496	54	463.0
38	adult_trait:skeleton phenotype(MP)	⇒	adult_trait:vision/eye phenotype(MP)	54	0.187	13	-108.3
85	adult_trait:limbs/digits/tail phenotype(MP)	⇒	adult_trait:craniofacial phenotype(MP)	16	0.174	11	-98.2
93	adult_trait:behavior/neurological phenotype(MP)	⇒	adult_trait:skeleton phenotype(MP)	39	0.196	8	-89.7

SD 13 Fig. 1g–i

g

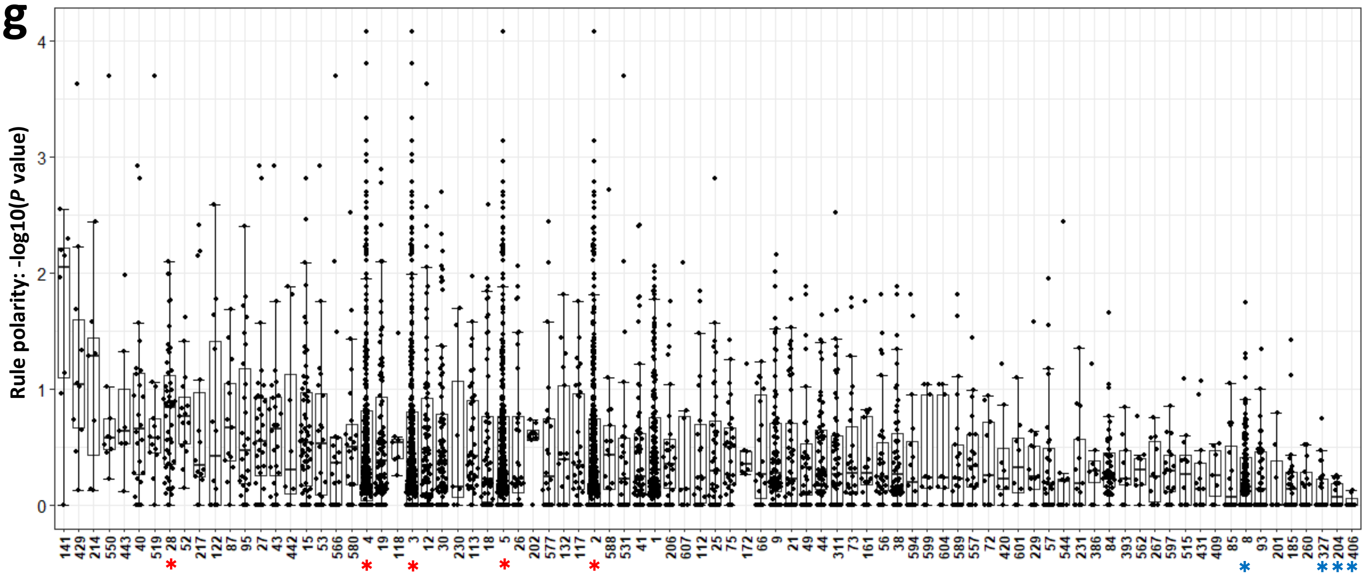

h

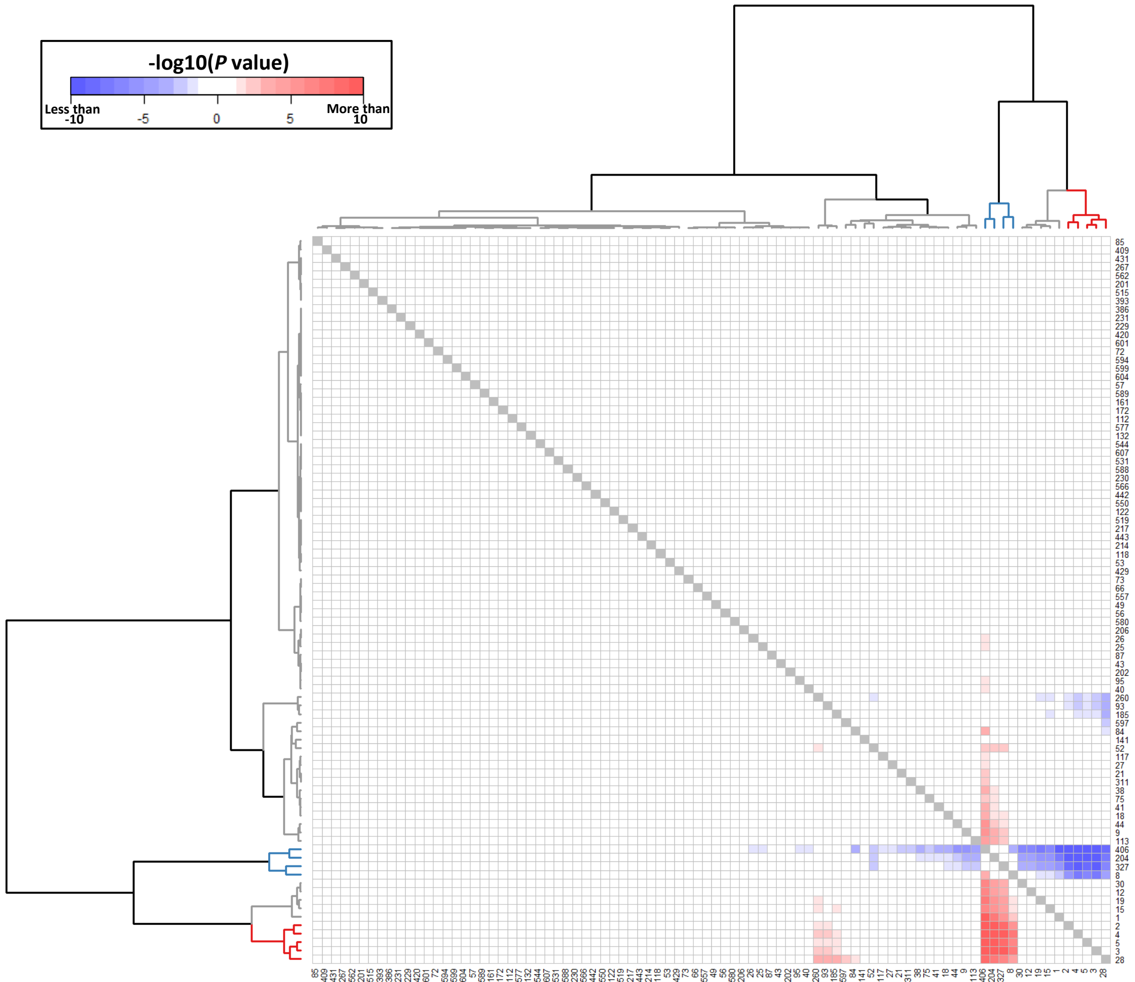

i

Label (enrichment rank)	Rules between biological systems			Num. of rules between phenotypes	Mean (rule polarity)	Statistics in post hoc test	
	Lhs_stage_type : top level term	Direction	Rhs_stage_type : top level term			Num. of significant differences ($P < 0.01$)	Accumulated -log10(P value)
28	adult_trait:homeostasis/metabolism phenotype(MP)	⇒	adult_trait:growth/size/body region phenotype(MP)	50	0.798	8	50.8
4	adult_trait:immune system phenotype(MP)	⇒	adult_trait:immune system phenotype(MP)	340	0.605	6	53.1
3	adult_trait:hematopoietic system phenotype(MP)	⇒	adult_trait:immune system phenotype(MP)	354	0.599	6	46.9
2	adult_trait:hematopoietic system phenotype(MP)	⇒	adult_trait:hematopoietic system phenotype(MP)	386	0.535	4	48.4
5	adult_trait:immune system phenotype(MP)	⇒	adult_trait:hematopoietic system phenotype(MP)	343	0.558	4	40.2
406	adult_trait:integument phenotype(MP)	⇒	adult_trait:skeleton phenotype(MP)	7	0.033	22	-161.9
204	adult_trait:behavior/neurological phenotype(MP)	⇒	adult_trait:craniofacial phenotype(MP)	14	0.100	14	-117.6
327	adult_trait:behavior/neurological phenotype(MP)	⇒	adult_trait:homeostasis/metabolism phenotype(MP)	27	0.126	13	-89.8
8	adult_trait:homeostasis/metabolism phenotype(MP)	⇒	adult_trait:homeostasis/metabolism phenotype(MP)	151	0.262	7	-40.7

SD 13 Fig. 1j–l

j

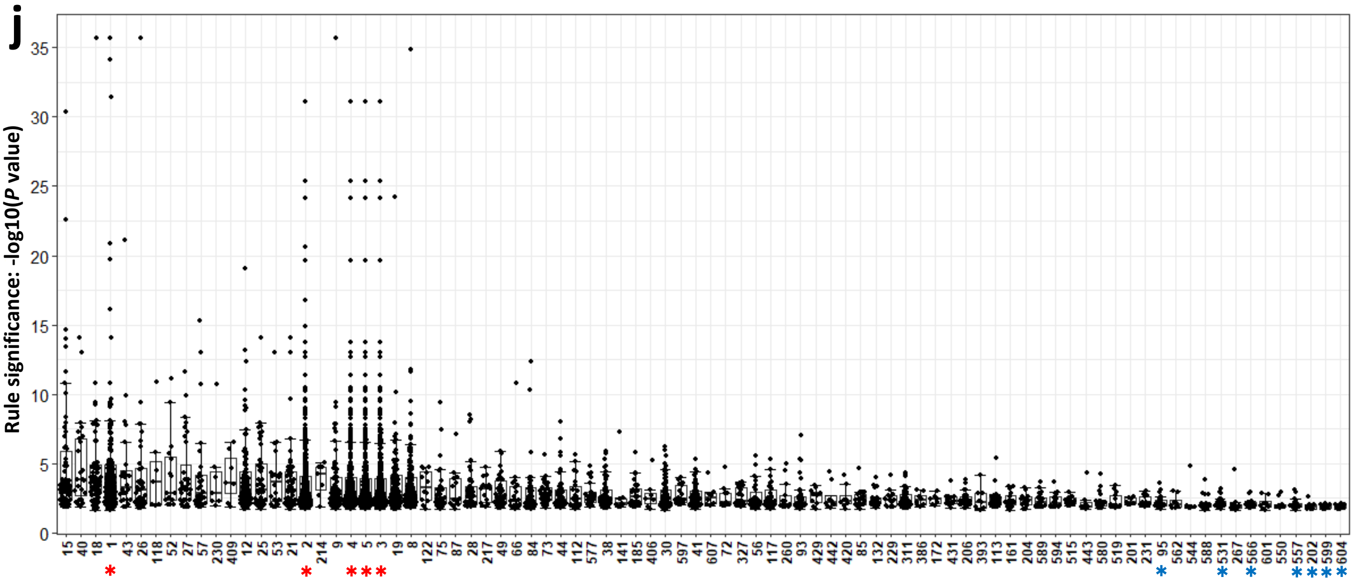

k

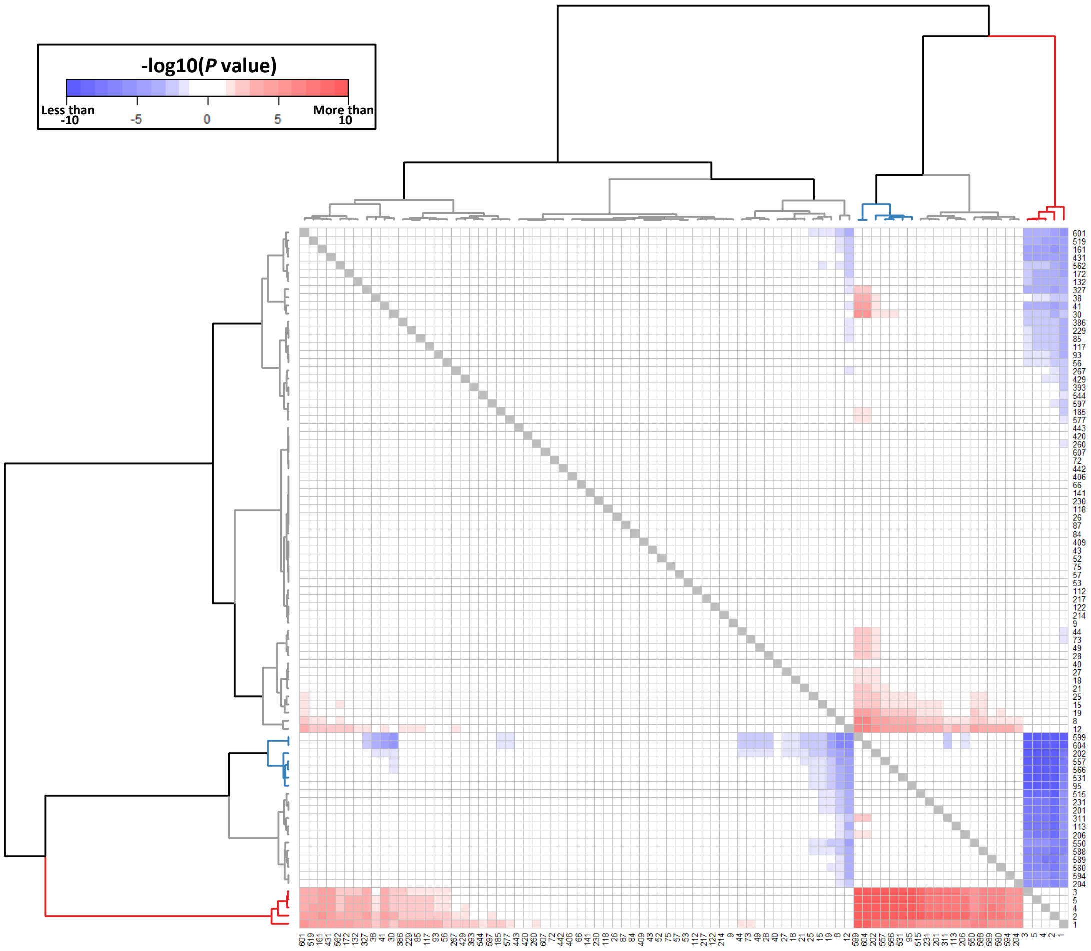

l

Label (enrichment rank)	Rules between biological systems: Lhs_stage_type : top level term	Direction	Rhs_stage_type : top level term	Num. of rules between phenotypes	Mean (rule significance)	Statistics in post hoc test: Num. of significant differences ($P < 0.01$)	Accumulated -log10(P value)
1	adult_trait:skeleton phenotype(MP)	⇒	adult_trait:skeleton phenotype(MP)	181	4.67	41	217.3
2	adult_trait:hematopoietic system phenotype(MP)	⇒	adult_trait:hematopoietic system phenotype(MP)	386	3.81	36	258.9
4	adult_trait:immune system phenotype(MP)	⇒	adult_trait:immune system phenotype(MP)	340	3.77	33	225.6
5	adult_trait:immune system phenotype(MP)	⇒	adult_trait:hematopoietic system phenotype(MP)	343	3.75	33	218.1
3	adult_trait:hematopoietic system phenotype(MP)	⇒	adult_trait:immune system phenotype(MP)	354	3.71	30	212.6
599	adult_trait:vision/eye phenotype(MP)	⇒	adult_trait:immune system phenotype(MP)	9	1.87	20	-137.8
604	adult_trait:vision/eye phenotype(MP)	⇒	adult_trait:hematopoietic system phenotype(MP)	9	1.87	20	-137.8
202	adult_trait:limbs/digits/tail phenotype(MP)	⇒	adult_trait:integument phenotype(MP)	8	1.94	10	-95.5
557	adult_trait:hematopoietic system phenotype(MP)	⇒	adult_trait:skeleton phenotype(MP)	20	2.07	8	-88.3
566	adult_trait:skeleton phenotype(MP)	⇒	adult_trait:immune system phenotype(MP)	17	2.12	8	-84.2
531	adult_trait:skeleton phenotype(MP)	⇒	adult_trait:hematopoietic system phenotype(MP)	25	2.15	8	-82.6
95	adult_trait:limbs/digits/tail phenotype(MP)	⇒	adult_trait:behavior/neurological phenotype(MP)	26	2.20	8	-69.7

SD 13 Fig. 1 Identifying between-‘biological systems’ rules exhibiting marked values for each of the four measures (support, confidence, rule polarity, rule significance) for ‘between-phenotypes’ rules for the ‘adult_trait => adult_trait’ between-‘stage/type’ rule. Of 244 kinds of between-‘biological systems’ rules in this between-‘stage/type’ rule, 86 where each rule category had a sample size ≥ 7 were applied for analysis. The between-‘biological systems’ rules examined were labeled according to rankings from the results of rule enrichment analysis for 608 kinds of between-‘biological systems’ rules (**Supplementary Table 6**). Results of analysis of values of each of the four measures (support, confidence, rule polarity, rule significance) for the ‘between-phenotypes’ rules are shown in **SD 13 Fig. 1a–c**, **SD 13 Fig. 1d–f**, **SD 13 Fig. 1g–i**, and **SD 13 Fig. 1j–l**, respectively. **(a,d,g,j)** Distributions of values for each of the four measures by between-‘biological systems’ rules. In these panels, for each of four measures, the 86 between-‘biological systems’ rules examined are arranged along the x-axis, in descending order (from the left) of mean value. Asterisks in red and blue represent between-‘biological systems’ rules with much larger/smaller values in the measure of interest, respectively. **(b,e,h,k)** Clustered heatmaps with dendrograms exhibiting mean differences in each measure among between-‘biological systems’ rules. By hierarchical clustering (Euclidean distance and Ward’s linkage) of between-‘biological systems’ rules, using the *P* values resulting from the post-hoc test (Games-Howell method) after one-way ANOVAs, between-‘biological systems’ rules with remarkably high/low mean values in each of support, confidence, rule polarity, and rule significance were identified. Note that the heatmap for each measure is symmetrical. Colored squares on heatmaps represent relationships with post-hoc $P < 0.05$, and darker colors represent greater differences between between-‘biological systems’ rules. For each dendrogram, sub-clusters in red and blue indicate between-‘biological systems’ rules with relatively greater and smaller values, respectively. **(c,f,i,l)** Between-‘biological systems’ rules with markedly greater/smaller values in each of the four measures. Between-‘biological systems’ rules identified here are arranged in descending order of the number of pairwise combinations with statistically significant differences. The High/Low columns in red (blue) indicate that the corresponding between-‘biological systems’ rules have greater (smaller) mean values for each measure. Refer to Methods for details.

SD 13 Fig. 2a–c

a

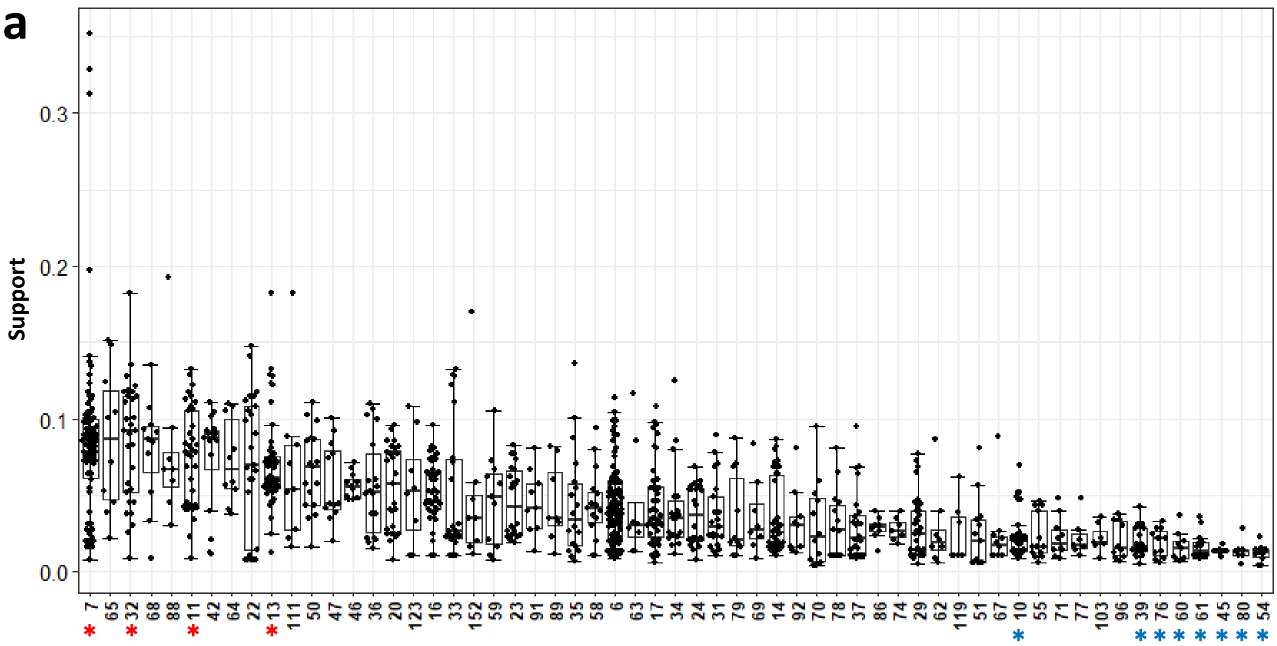

b

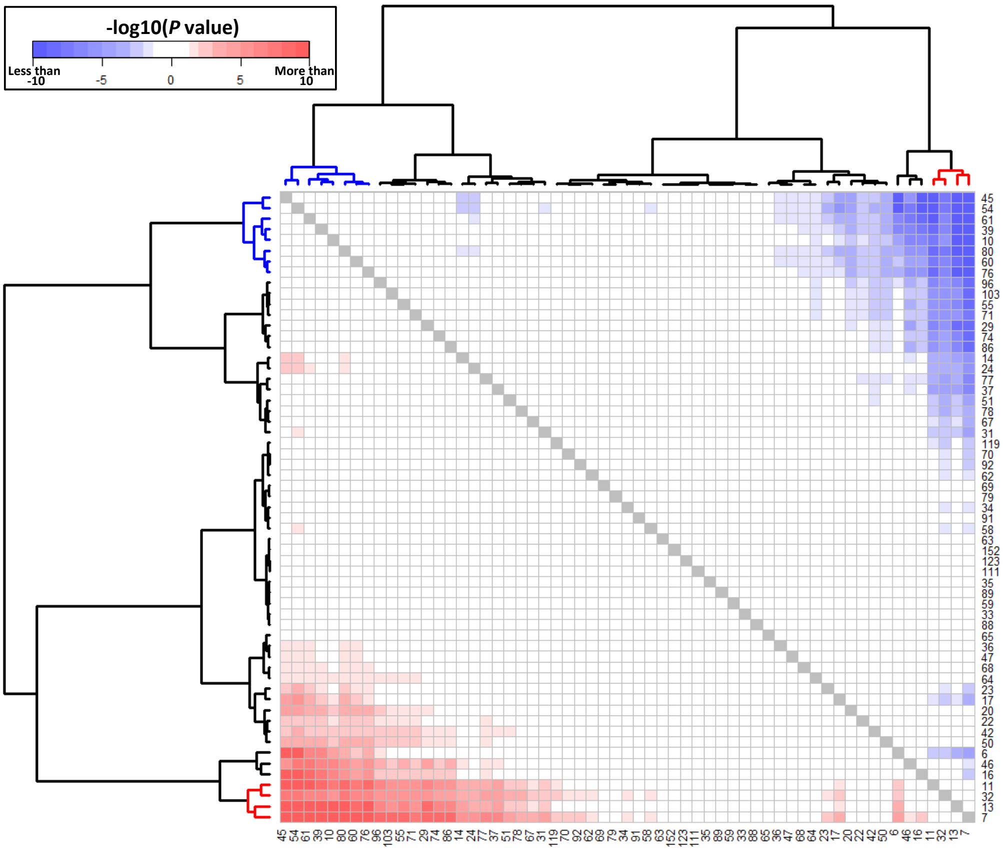

c

Label (enrichment rank)	Rules between biological systems: Lhs_stage_type : top level term	Direction	Rhs_stage_type : top level term	Num. of rules between phenotypes	Mean (support)	Statistics in post hoc test: Num. of significant differences ($P < 0.01$)	Accumulated -log10(P value)
7	adult_gene:nervous system(MA)	⇒	adult_gene:nervous system(MA)	90	0.087	30	211.2
32	adult_gene:nervous system(MA)	⇒	adult_gene:reproductive system(MA)	30	0.086	26	140.9
13	adult_gene:reproductive system(MA)	⇒	adult_gene:nervous system(MA)	50	0.070	23	176.9
11	adult_gene:endocrine system(MA)	⇒	adult_gene:nervous system(MA)	37	0.075	23	150.4
45	adult_gene:hemolymphoid system(MA)	⇒	adult_gene:nervous system(MA)	11	0.014	15	-116.5
54	adult_gene:immune system(MA)	⇒	adult_gene:set of connective tissues(MA)	10	0.012	15	-115.8
61	adult_gene:exocrine system(MA)	⇒	adult_gene:digestive system(MA)	14	0.016	13	-92.7
80	adult_gene:hemolymphoid system(MA)	⇒	adult_gene:digestive system(MA)	7	0.014	13	-87.5
39	adult_gene:immune system(MA)	⇒	adult_gene:digestive system(MA)	20	0.019	12	-87.7
60	adult_gene:immune system(MA)	⇒	adult_gene:cardiovascular system(MA)	9	0.016	12	-71.8
76	adult_gene:set of connective tissues(MA)	⇒	adult_gene:digestive system(MA)	11	0.019	11	-71.3
10	adult_gene:immune system(MA)	⇒	adult_gene:nervous system(MA)	43	0.023	10	-72.4

SD 13 Fig. 2d–f

d

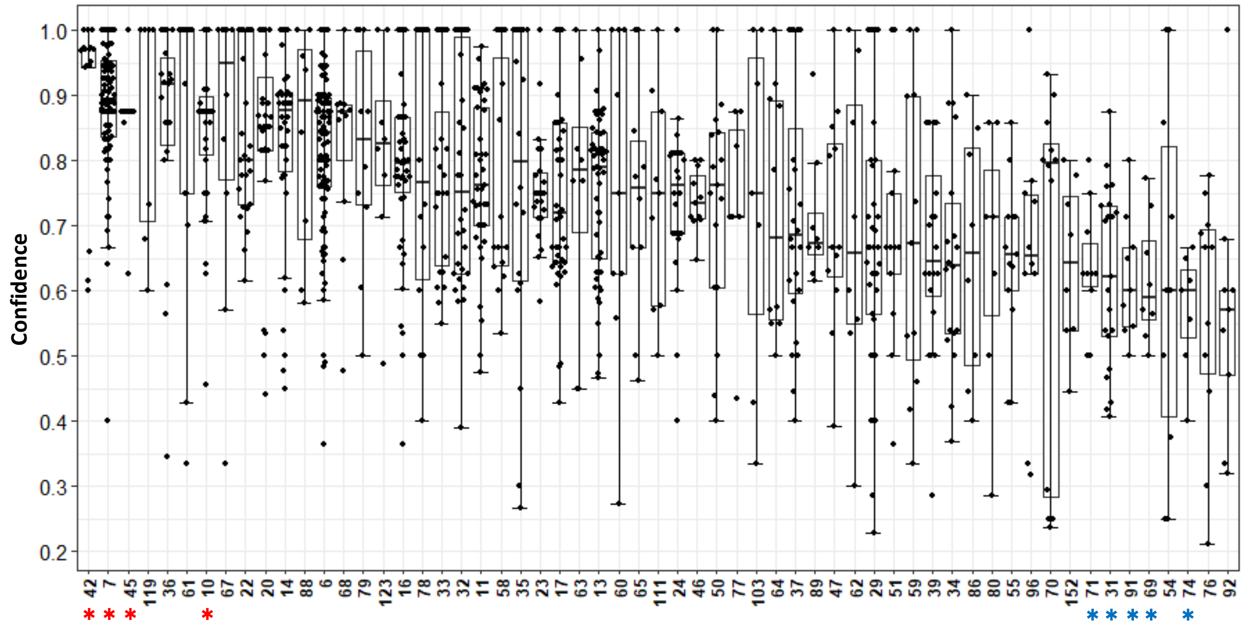

e

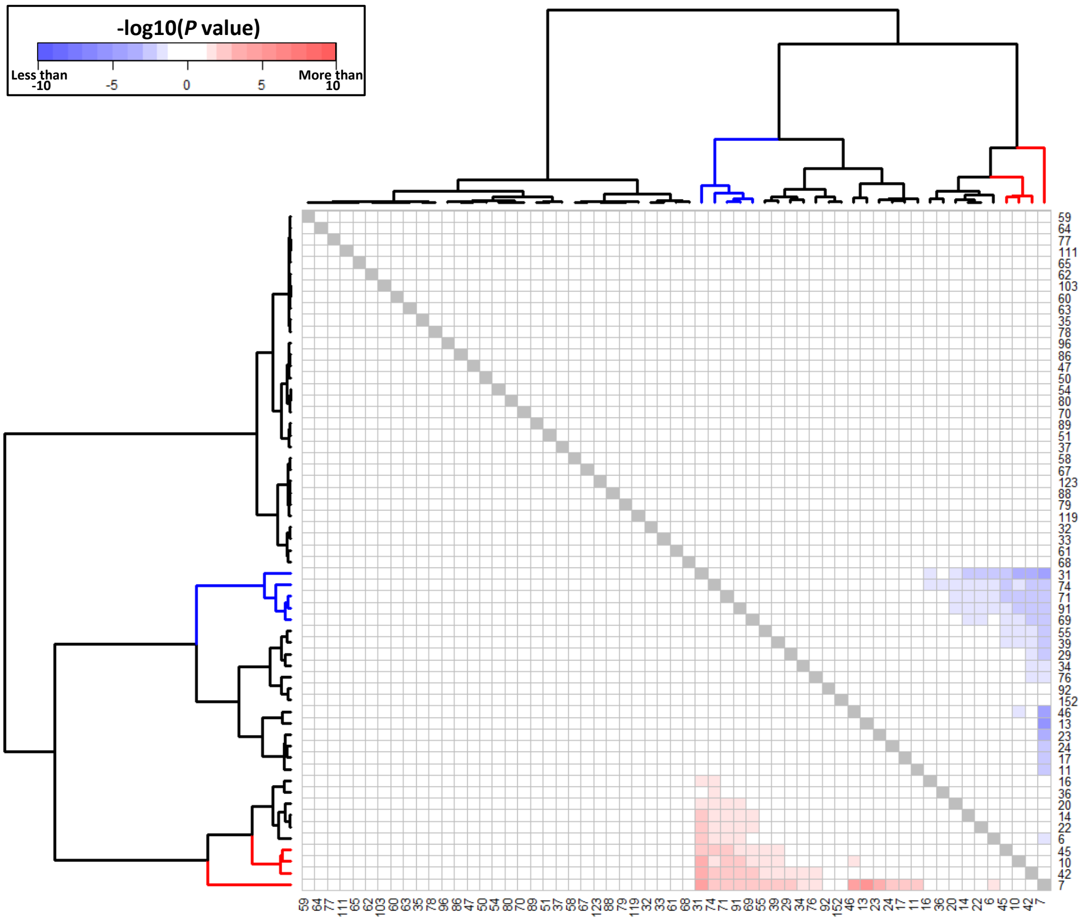

f

Label (enrichment rank)	Rules between biological systems: Lhs_stage_type : top level term	Direction	Rhs_stage_type : top level term	Num. of rules between phenotypes	Mean (confidence)	Statistics in post hoc test: Num. of significant differences ($P < 0.01$)	Accumulated -log10(P value)
7	adult_gene:nervous system(MA)	⇒	adult_gene:nervous system(MA)	90	0.89	14	56.7
42	adult_gene:nervous system(MA)	⇒	adult_gene:cardiovascular system(MA)	16	0.91	5	30.4
10	adult_gene:immune system(MA)	⇒	adult_gene:nervous system(MA)	43	0.85	3	24.7
45	adult_gene:hemolymphoid system(MA)	⇒	adult_gene:nervous system(MA)	11	0.86	3	21.4
31	adult_gene:digestive system(MA)	⇒	adult_gene:endocrine system(MA)	21	0.62	7	-31.9
71	adult_gene:immune system(MA)	⇒	adult_gene:respiratory system(MA)	10	0.63	4	-21.2
74	adult_gene:set of connective tissues(MA)	⇒	adult_gene:musculoskeletal system(MA)	7	0.57	3	-30.9
91	adult_gene:musculoskeletal system(MA)	⇒	adult_gene:digestive system(MA)	9	0.62	3	-22.0
69	adult_gene:digestive system(MA)	⇒	adult_gene:trunk(MA)	8	0.62	2	-19.5

SD 13 Fig. 2g–i

g

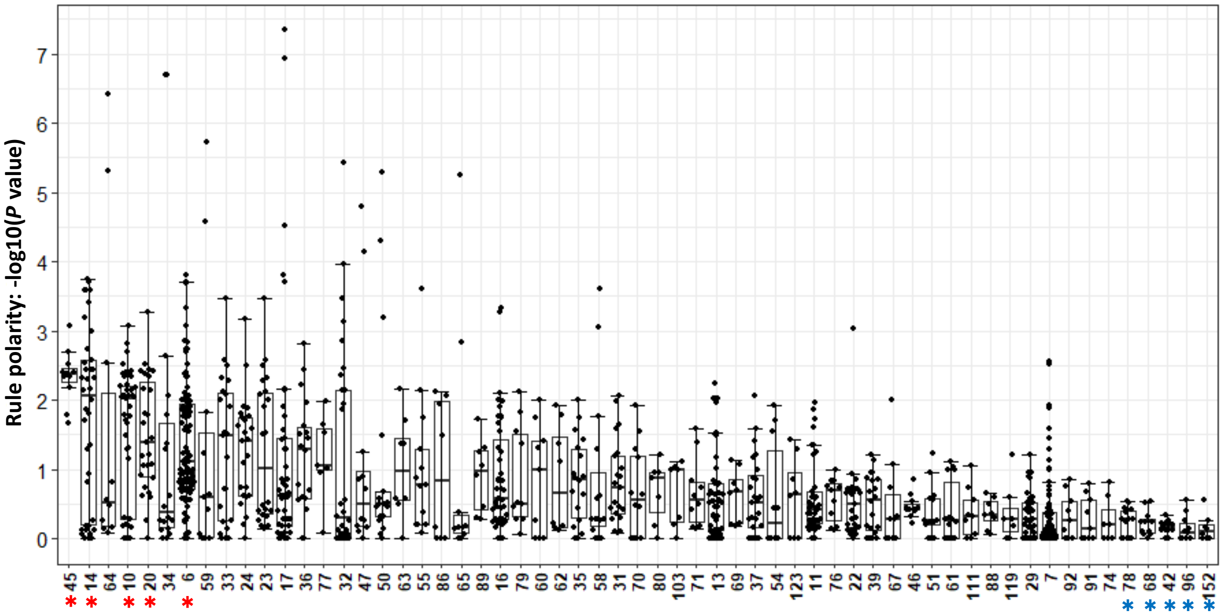

h

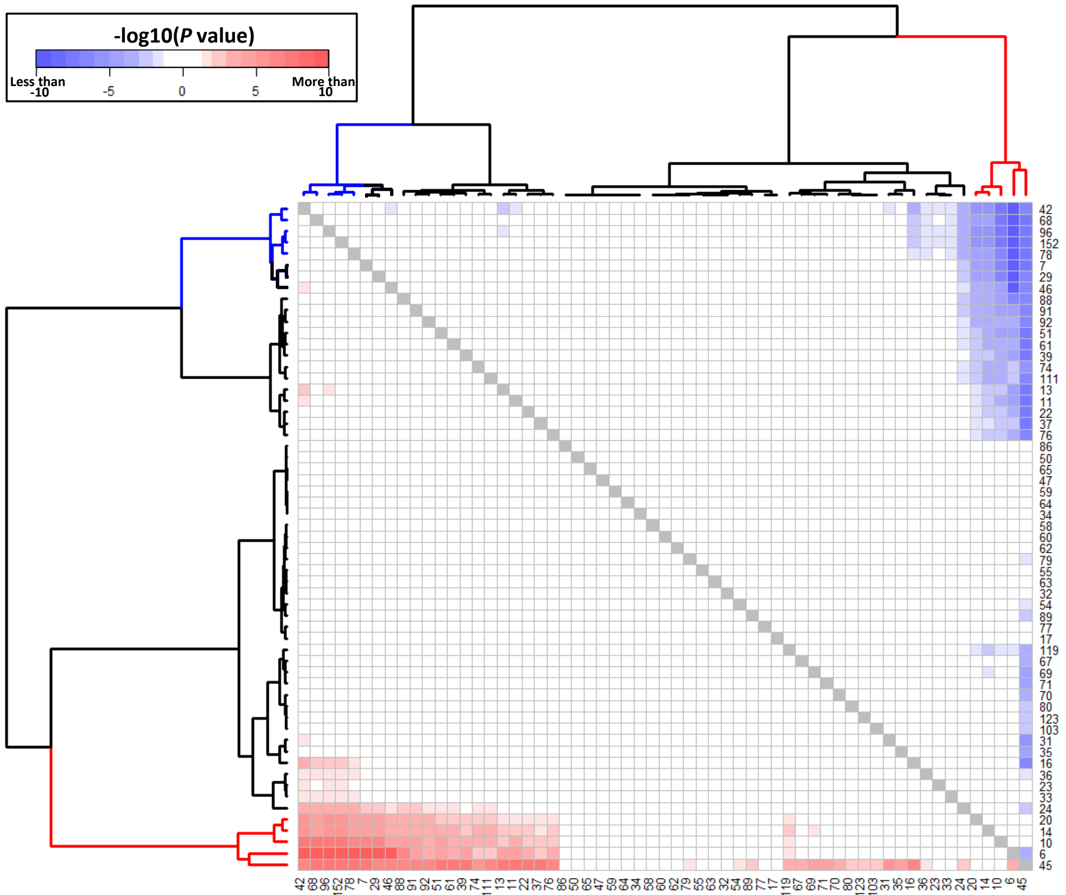

i

Label (enrichment rank)	Rules between biological systems: Lhs_stage_type : top level term	Direction	Rhs_stage_type : top level term	Num. of rules between phenotypes	Mean (rule polarity)	Statistics in post hoc test: Num. of significant differences ($P < 0.01$)	Accumulated -log10(P value)
45	adult_gene:hemolymphoid system(MA)	⇒	adult_gene:nervous system(MA)	11	2.34	35	216.3
6	adult_gene:digestive system(MA)	⇒	adult_gene:nervous system(MA)	102	1.37	22	147.3
10	adult_gene:immune system(MA)	⇒	adult_gene:nervous system(MA)	43	1.53	21	105.7
14	adult_gene:set of connective tissues(MA)	⇒	adult_gene:nervous system(MA)	38	1.76	21	87.5
20	adult_gene:musculoskeletal system(MA)	⇒	adult_gene:nervous system(MA)	27	1.49	16	74.3
42	adult_gene:nervous system(MA)	⇒	adult_gene:cardiovascular system(MA)	16	0.16	8	-66.8
96	adult_gene:digestive system(MA)	⇒	adult_gene:immune system(MA)	10	0.15	7	-58.4
152	adult_gene:nervous system(MA)	⇒	adult_gene:respiratory system(MA)	8	0.15	7	-55.8
68	adult_gene:nervous system(MA)	⇒	adult_gene:urinary system(MA)	11	0.22	7	-54.9
78	adult_gene:respiratory system(MA)	⇒	adult_gene:digestive system(MA)	14	0.24	6	-47.0

SD 13 Fig. 2j–l

j

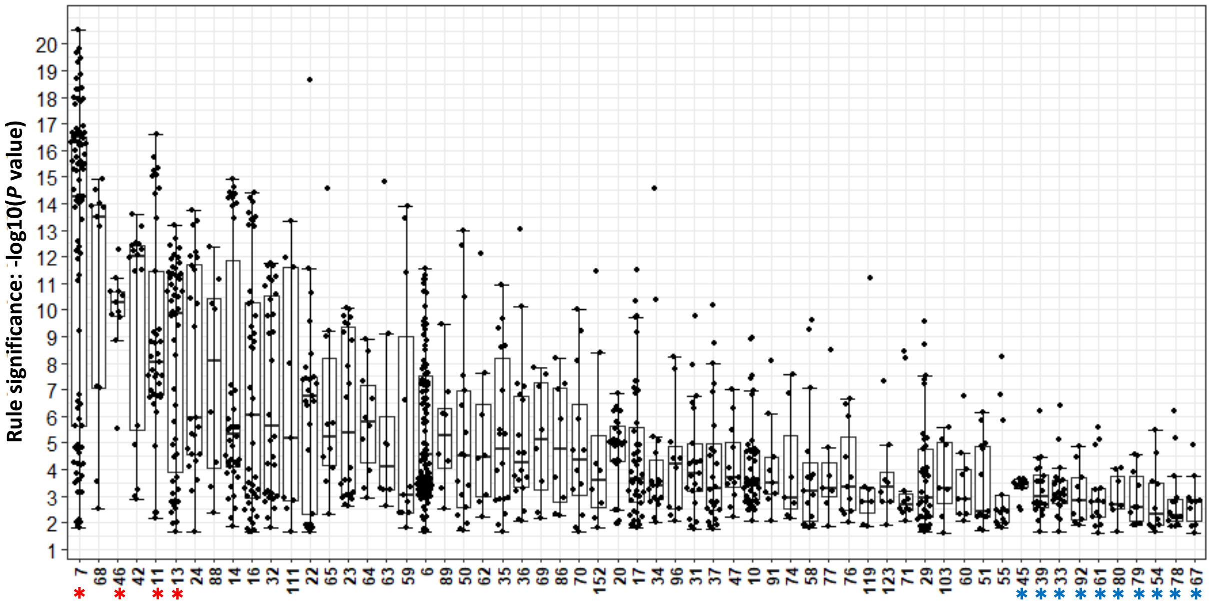

k

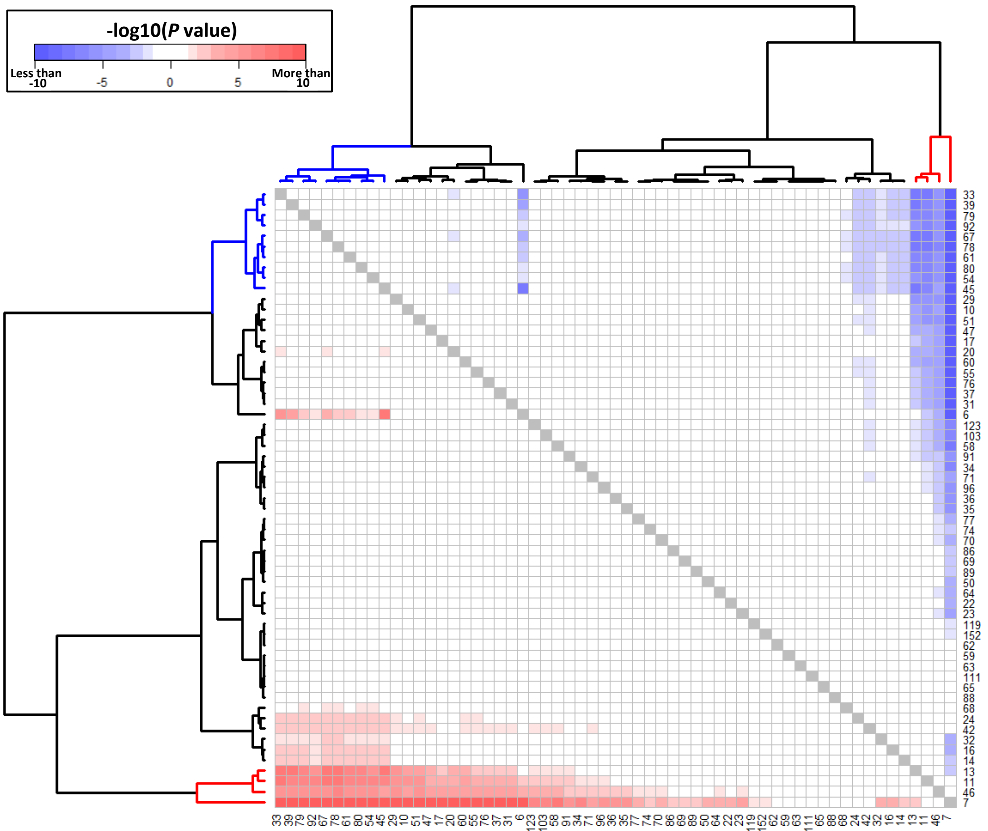

l

Label (enrichment rank)	Rules between biological systems			Num. of rules between phenotypes	Mean (rule significance)	Statistics in post hoc test	
	Lhs_stage_type : top level term	Direction	Rhs_stage_type : top level term			Num. of significant differences ($P < 0.01$)	Accumulated -log10(P value)
7	adult_gene:nervous system(MA)	⇒	adult_gene:nervous system(MA)	90	11.97	45	361.1
46	adult_gene:trunk(MA)	⇒	adult_gene:nervous system(MA)	11	9.95	31	152.4
11	adult_gene:endocrine system(MA)	⇒	adult_gene:nervous system(MA)	37	9.06	26	140.8
13	adult_gene:reproductive system(MA)	⇒	adult_gene:nervous system(MA)	50	8.06	22	116.9
67	adult_gene:exocrine system(MA)	⇒	adult_gene:respiratory system(MA)	10	2.79	10	-55.4
78	adult_gene:respiratory system(MA)	⇒	adult_gene:digestive system(MA)	14	2.87	10	-51.5
33	adult_gene:exocrine system(MA)	⇒	adult_gene:nervous system(MA)	23	3.19	9	-56.3
79	adult_gene:exocrine system(MA)	⇒	adult_gene:reproductive system(MA)	10	2.97	9	-54.2
39	adult_gene:immune system(MA)	⇒	adult_gene:digestive system(MA)	20	3.25	9	-54.1
45	adult_gene:hemolymphoid system(MA)	⇒	adult_gene:nervous system(MA)	11	3.30	9	-52.2
61	adult_gene:exocrine system(MA)	⇒	adult_gene:digestive system(MA)	14	3.05	9	-47.4
80	adult_gene:hemolymphoid system(MA)	⇒	adult_gene:digestive system(MA)	7	2.99	8	-47.3
54	adult_gene:immune system(MA)	⇒	adult_gene:set of connective tissues(MA)	10	2.90	8	-45.6
92	adult_gene:digestive system(MA)	⇒	adult_gene:musculoskeletal system(MA)	9	3.07	6	-49.0

SD 13 Fig. 2 Identifying between-‘biological systems’ rules exhibiting marked values in each of four measures (support, confidence, rule polarity, rule significance) for ‘adult_gene => adult_gene’ between-‘stage/type’ ‘between-phenotypes’ rules. Of 200 kinds of between-‘biological systems’ rules in this between-‘stage/type’ rule, 56 where each rule category had sample size ≥ 7 were applied for analysis. The between-‘biological systems’ rules examined were labeled according to rankings from the results of rule enrichment analysis for 608 kinds of between-‘biological systems’ rules (**Supplementary Table 6**). Results of analysis of values for each of the four measures (support, confidence, rule polarity, rule significance) for the ‘between-phenotypes’ rules are shown in **SD 13 Fig. 2a–c**, **2d–f**, **2g–i**, and **2j–l**, respectively. **(a,d,g,j)** Distributions of values for each of the four measures, according to between-‘biological systems’ rules. In these panels, the 86 between-‘biological systems’ rules examined are arranged along the x-axis in descending order (from the left) of mean value for each of the four measures. Asterisks in red and blue represent between-‘biological systems’ rules with extremely large/small values in the measure of interest, respectively. **(b,e,h,k)** Clustered heatmaps with dendrograms exhibiting mean differences in each measure among between-‘biological systems’ rules. By hierarchical clustering (Euclidean distance and Ward’s linkage) of between-‘biological systems’ rules using the *P* values resulting from the post-hoc test (Games-Howell method) after one-way ANOVAs, between-‘biological systems’ rules with remarkably high/low mean values in each of support, confidence, rule polarity, and rule significance were identified. Note that the heatmaps for each measure are symmetrical. Colored squares on heatmaps represent relationships with post-hoc $P < 0.05$, and darker colors represent greater differences between between-‘biological systems’ rules. For each dendrogram, sub-clusters in red and blue represent between-‘biological systems’ rules having relatively greater or smaller values, respectively. **(c,f,i,l)** Between-‘biological systems’ rules with markedly greater/smaller values for each of the four measures. Between-‘biological systems’ rules identified here are arranged in descending order, according to the number of pairwise combinations with statistically significant differences. The High/Low columns in red (blue) indicate that the corresponding between-‘biological systems’ rules have greater (smaller) mean values for each measure. Refer to Methods for details.

SD 13 Fig. 3a–d

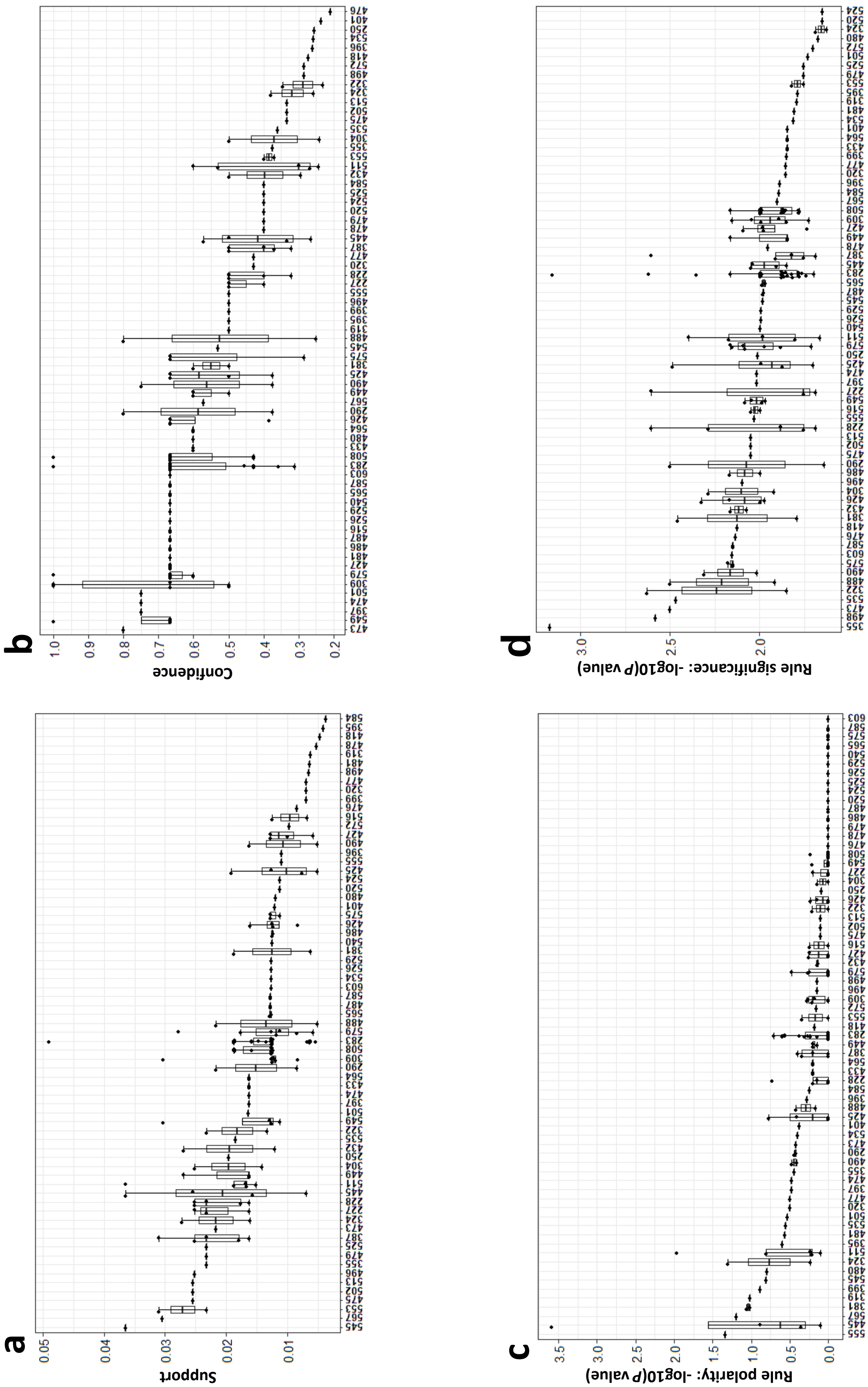
a
Support
b
Confidence
c
Rule polarity: -log10(P value)
d
Rule significance: -log10(P value)

SD 13 Fig. 3 Distributions of values in each of the four measures for 69 ‘adult_trait => adult_gene’ between-‘stage/type’ between-‘biological systems’ rules. Distributions of values for support, confidence, rule polarity, and rule significance are displayed in **a**, **b**, **c**, and **d**, respectively. The 69 between-‘biological systems’ rules are labeled with ranked numbers (defined in **Supplementary Table 6**). In all panels, the 69 between-‘biological systems’ rules are arranged along the x-axis in descending order (from the left) of mean values.

SD 13 Fig. 4a–d

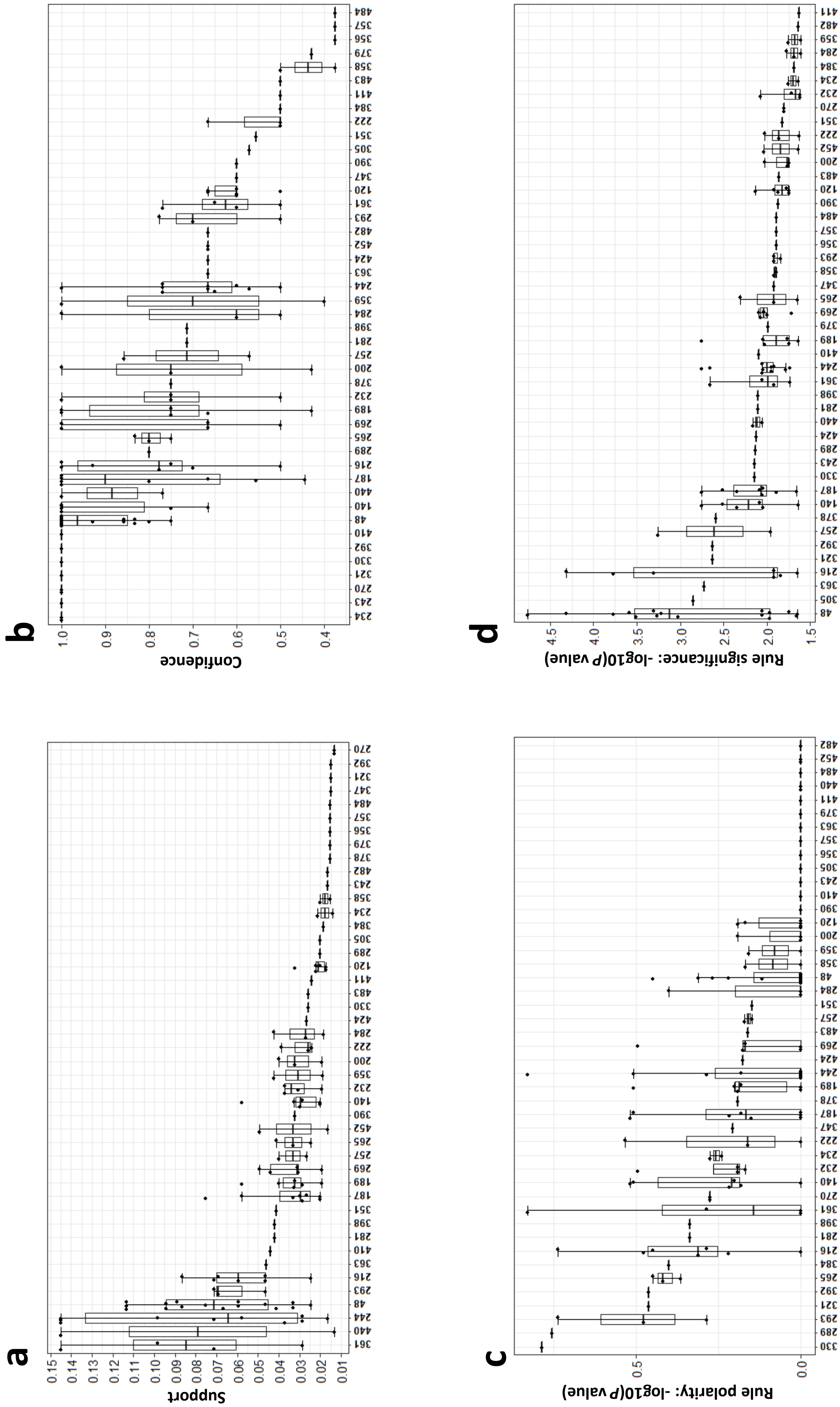
a
Support
b
Confidence
c
Rule polarity: -log10(P value)
d
Rule significance: -log10(P value)

SD 13 Fig. 4 Distributions of values in each of the four measures for 45 'embryo_trait => embryo_trait' between-'stage/type' between-'biological systems' rules. Distributions of values for support, confidence, rule polarity, and rule significance are displayed in **a**, **b**, **c**, and **d**, respectively. The 45 between-'biological systems' rules are labeled with ranked numbers (defined in **Supplementary Table 6**). In all panels, the 45 between-'biological systems' rules examined are arranged along the x-axis in descending order (from the left) of mean value.

SD 13 Fig. 5a–d

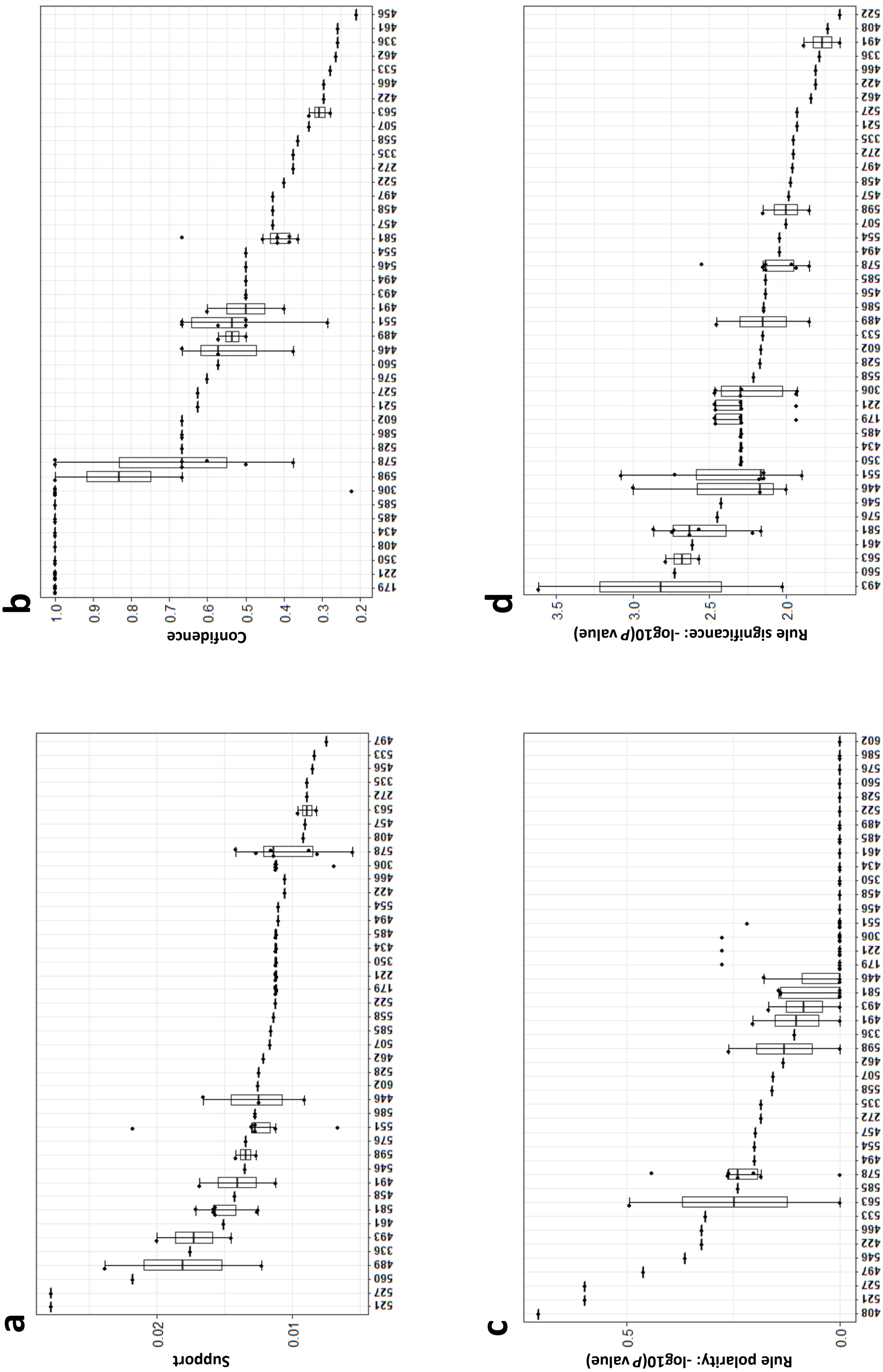
a
Support
b
Confidence
c
Rule polarity: -log10(P value)
d
Rule significance: -log10(P value)

SD 13 Fig. 5 Distributions of values in each of the four measures for 42 'adult_gene => adult_trait' between-'stage/type' between-'biological systems' rules. Distributions of values for support, confidence, rule polarity, and rule significance are displayed in **a**, **b**, **c**, and **d**, respectively. The 42 between-'biological systems' rules are labeled with ranked numbers (defined in **Supplementary Table 6**). In all panels, the 42 between-'biological systems' rules examined are arranged along the x-axis in descending order (from the left) of mean value.

Supplementary Data 14

Comparative analysis of PPAP-constituting phenotypes according to 'stage/type' and 'biological system' query phenotype categories.

We examined the numbers of phenotypes and distinct biological systems for the 345 PPAPs, corresponding to the 345 query phenotypes, to clarify whether differences in characteristics between query phenotypes produced differences in related phenotype characteristics, according to 'stage/type' and 'biological systems' query phenotype categories (**Supplementary Table 14**, and **SD14 Fig. 1**).

Multiple comparison tests among three 'stage/type' query phenotype categories (excluding the 'embryo/gene' category, which only had three phenotypes) revealed significant differences across all combinations of the three 'stage/type' categories, in numbers of both phenotypes and distinct biological systems (**SD14 Fig. 1a, b** and **Supplementary Table 15**) of PPAP-constituting phenotypes, with values for both measures in the order (high to low) 'adult/gene' > 'adult/trait' > 'embryo/trait'. Enrichment analyses for each PPAP query phenotype, corresponding to the top 10% of both measures (**Supplementary Table 16**), demonstrated that 'adult/gene' category phenotypes were significantly enriched for both measures (one-tailed Fisher's exact test, $P = 2.8\times10^{-4}$ for phenotype, and $P = 8.9\times10^{-8}$ for distinct biological systems, numbers), indicating that 'adult/gene' phenotypes have significantly more relationships with abnormal phenotypes and biological systems than 'adult/trait' and 'embryo/trait' phenotypes.

Based on the results of multiple comparison tests (see above), we compared numbers of phenotypes and biological systems, according to the 'stage/type' PPAP query phenotype (**SD14 Fig. 1c-f** and **Supplementary Table 17**). These analysis results are shown below:

Among 'adult/trait' phenotypes, those with more related phenotypes belonged to the biological system 'adult_trait:skeleton phenotype(MP)' (**SD14 Fig. 1c, d**), indicating that skeleton system abnormality phenotypes have more relationships with other abnormal phenotypes than those in other biological systems. Numbers of distinct biological systems in phenotype-constituting PPAPs were remarkably large for 'adult_trait:skeleton phenotype(MP)' and 'adult_trait:vision/eye phenotype(MP)' query phenotypes (**SD14 Fig. 1e, f**), indicating that abnormal phenotypes in these systems are related to phenotypes spanning a wide range of biological systems. Conversely, equivalent numbers were remarkably small for 'adult_trait:hematopoietic system phenotype(MP)' and 'adult_trait:immune system phenotype(MP)' query phenotypes (**SD14 Fig. 1e, f**), indicating that abnormal phenotypes in these systems are related to phenotypes spanning fewer biological systems. Indeed, among all 253 PPAPs comprising seven or more phenotypes, 20 PPAPs consisting of the smallest number (2) of biological systems contained only phenotypes in the biological systems 'adult_trait:hematopoietic system phenotype(MP)' and 'adult_trait:immune system phenotype(MP)' (**Supplementary Table 14**). Of the 20 query phenotypes for these 20 PPAPs, 19 correspond to various immuno-phenotyping (FACs) test measured parameters; one PPAP comprised a maximum of 24 of these phenotypes (query phenotype: 'adult_trait:abnormal NK cell number').

In contrast, for 'adult/gene' query phenotypes, we observed no significant difference among its biological

systems in either number of phenotypes related to the query or number of distinct biological systems among phenotypes comprising the PPAP (P = 0.077 and P = 0.18, respectively; ANOVA) (**Supplementary Table 17**), indicating no distinctive features of these two measures between biological systems.

SD14 Fig. 1 a-f

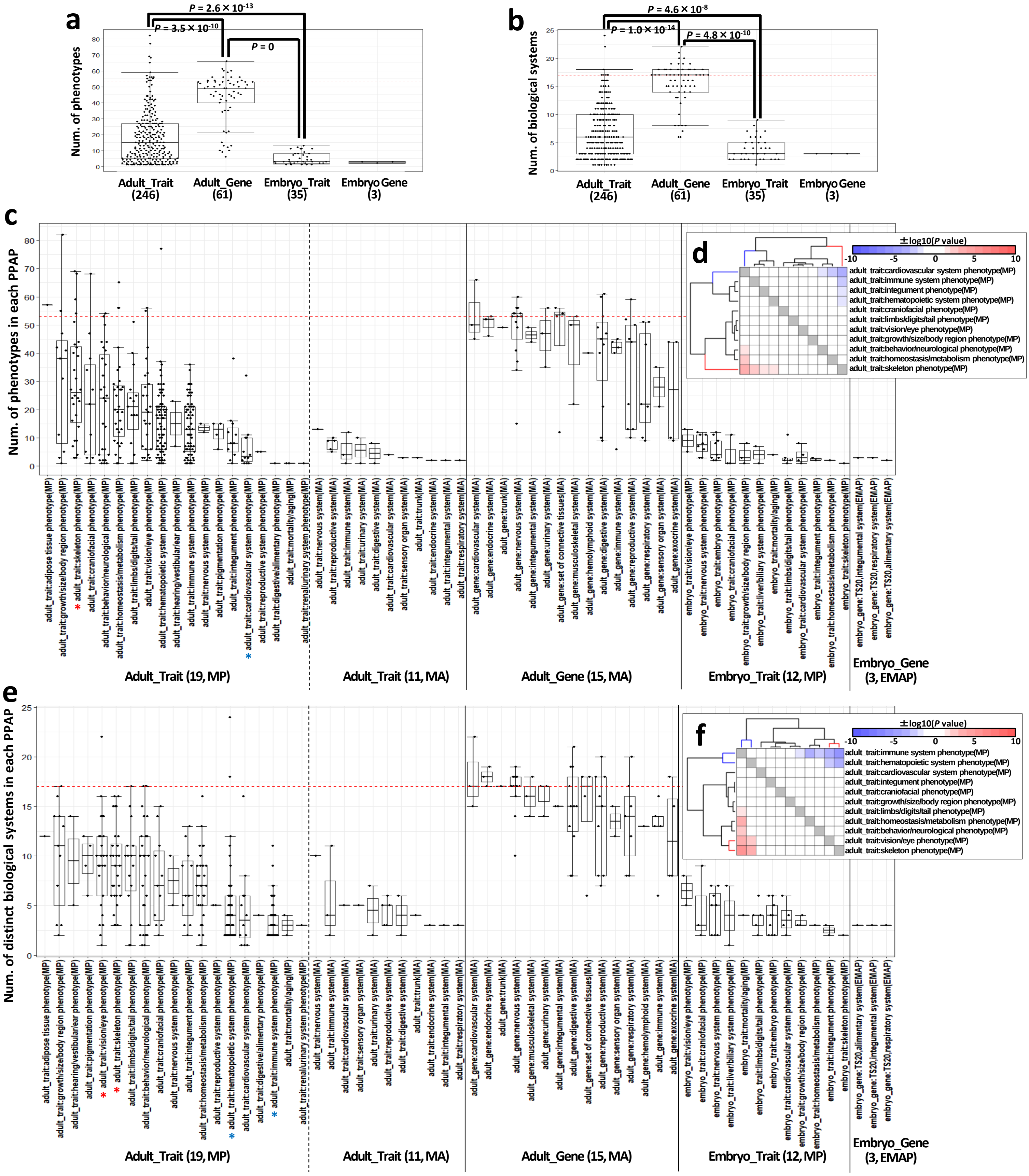
a
P = 2.6 × 10^-13
P = 3.5 × 10^-10
P = 0
Num. of phenotypes
Adult_Trait (246)
Adult_Gene (61)
Embryo_Trait (35)
Embryo Gene (3)
b
P = 4.6 × 10^-8
P = 1.0 × 10^-14
P = 4.8 × 10^-10
Num. of biological systems
Adult_Trait (246)
Adult_Gene (61)
Embryo_Trait (35)
Embryo Gene (3)
c
Num. of phenotypes in each PPAP
Adult_Trait (19, MP)
Adult_Trait (11, MA)
Adult_Gene (15, MA)
Embryo_Trait (12, MP)
Embryo_Gene (3, EMAP)
d
±log10(P value)
adult_trait:cardiovascular system phenotype(MP)
adult_trait:immune system phenotype(MP)
adult_trait:integument phenotype(MP)
adult_trait:hematopoietic system phenotype(MP)
adult_trait:craniofacial phenotype(MP)
adult_trait:limbs/digits/tail phenotype(MP)
adult_trait:vision/eye phenotype(MP)
adult_trait:growth/size/body region phenotype(MP)
adult_trait:behavior/neurological phenotype(MP)
adult_trait:homeostasis/metabolism phenotype(MP)
adult_trait:skeleton phenotype(MP)
e
Num. of distinct biological systems in each PPAP
Adult_Trait (19, MP)
Adult_Trait (11, MA)
Adult_Gene (15, MA)
Embryo_Trait (12, MP)
Embryo_Gene (3, EMAP)
f
±log10(P value)
adult_trait:immune system phenotype(MP)
adult_trait:hematopoietic system phenotype(MP)
adult_trait:cardiovascular system phenotype(MP)
adult_trait:integument phenotype(MP)
adult_trait:craniofacial phenotype(MP)
adult_trait:growth/size/body region phenotype(MP)
adult_trait:limbs/digits/tail phenotype(MP)
adult_trait:homeostasis/metabolism phenotype(MP)
adult_trait:behavior/neurological phenotype(MP)
adult_trait:vision/eye phenotype(MP)
adult_trait:skeleton phenotype(MP)

SD14 Fig. 1 Comparative analysis of PPAP-constituting phenotypes according to 'stage/type' and 'biological system' query phenotype categories. (**a,b**) For each of 345 PPAPs, distributions of the numbers of PPAP-constituting phenotypes (**a**) and numbers of PPAP-constituting distinct biological systems **(b)** are shown according to the four 'stage/type' query phenotype categories. Red broken lines indicate thresholds corresponding to the top 10% for each value measured. *P* values for comparisons between 'stage/type' categories are derived from the post-hoc test (Games-Howell method). Refer to **Supplementary Table 14** and Methods for details. (**c–f**) Comparative analysis of PPAP-constituting phenotypes by biological system categorization of the 345 PPAP query phenotypes. (**c,e**) For each of the 345 PPAPs, distributions of the numbers of PPAP-constituting phenotypes (**c**) and numbers of PPAP-constituting distinct biological systems (**e**) are shown according to the 60 biological systems of query phenotypes for the 345 PPAPs. The 60 biological systems are arranged along the x-axis in descending order (from the left) of mean value. Red broken lines, thresholds corresponding to the top 10% for each value measured. Asterisks in red/blue, biological systems identified by the following analyses (**d** and **f**) as exhibiting relatively greater/smaller mean values. (**d,f**) Biological systems exhibiting marked values for the number of PPAP-constituting phenotypes (**d**) and the number of PPAP-constituting distinct biological systems (**f**) for 'adult/trait' phenotypes. Of 30 kinds of biological system in the 'adult/trait' category, only 11, where each biological systems category had a sample size ≥ 7, were applied for analysis. For each measure, clustered heatmaps with dendrograms are displayed, exhibiting mean differences among biological systems. Biological systems with remarkably high/low mean values in each of the two measures were identified by hierarchical clustering (Euclidean distance and Ward's linkage) of biological systems using *P* values resulting from the post-hoc test (Games-Howell method) after one-way ANOVAs. Note that the heatmap for each measure is symmetrical. Colored squares on heatmaps represent relationships with post-hoc $P < 0.05$, and darker colors represent greater differences between biological systems. For each dendrogram, sub-clusters in red and blue represent biological systems with relatively greater and smaller values, respectively.

Supplementary Data 15

Details of the seven clusters classified by hierarchical clustering based on similarities among 345 sets of PPAP-constituting phenotypes

By examining similarities between 345 sets of PPAP-constituting phenotypes, we can obtain information about positional closeness among the 345 PPAP-derived query phenotypes in the putative phenome-wide phenotypic association network. Based on these similarities, we performed hierarchical clustering of the 345 PPAPs. Consequently, we identified seven clusters within the putative phenome-wide phenotypic association network (**Fig. 6**). We present details of these clusters (clusters 1–7) below.

Note: For detailed information about each PPAP, refer to **Supplementary Table 2** and the web application developed in this study (https://brc-riken.shinyapps.io/phenotypic_associations_across_the_mouse_phenome/). For explanations of the graphical representation of each PPAP, see the legends of both **Fig. 4a** and **Supplementary Fig. 3**.

++

Cluster 1

- Cluster 1 consisted of 63 phenotypes (**Supplementary Table 2**). All phenotypes annotated with the 'stage/type' 'adult/gene' (n = 61) belonged to this cluster and were significantly enriched in this cluster (61/63 vs. 61/345, two-tailed Fisher's exact test, $P = 6.6\times10^{-14}$). Therefore, in this cluster, the number of phenotypes classified in the 'adult/gene' 'stage/type' category is highly statistically significant.
- Enrichment analysis of phenotypes in this cluster according to their biological systems revealed statistically significant enrichment of phenotypes belonging to the biological systems 'adult_gene:nervous system(MA)', 'adult_gene:digestive system(MA)', 'adult_gene:reproductive system(MA)', and 'adult_gene:respiratory system(MA)' (see table below for details).
- In this study, all of the phenotypes annotated with the category 'adult/gene' were derived from measured parameters in the adult LacZ test. Therefore, this cluster mainly consists of abnormal phenotypes detected by the adult LacZ test.
- In this cluster, the remaining two phenotypes that did not belong to the phenotypic category 'adult/gene' were 'adult_trait:abnormal spleen weight(MP)' and 'embryo_trait:abnormal viability by preweaning(MP)'. Both of these phenotypes had numerous relationships with phenotypes belonging to the category 'adult/gene' (see the figure below); hence the two phenotypes were included in this cluster.

Enrichment analysis of phenotypes in cluster 1 according to their biological systems

stage_type:top level term	Ratio in cluster 1	Ratio in total	Fold enrichment	*P* value	Bonferroni
adult_gene:nervous system(MA)	15/76	15/453	5.960526316	9.9E-06	0.00015832***
adult_gene:digestive system(MA)	11/76	11/453	5.960526316	0.00013	0.00213812**
adult_gene:reproductive system(MA)	9/76	9/453	5.960526316	0.00052	0.00832636**
adult_gene:respiratory system(MA)	7/76	7/453	5.960526316	0.00213	0.03403011*
adult_gene:immune system(MA)	5/76	5/453	5.960526316	0.00926	0.14815396
adult_gene:exocrine system(MA)	4/76	4/453	5.960526316	0.01997	0.31945738
adult_gene:set of connective tissues(MA)	4/76	4/453	5.960526316	0.01997	0.31945738
adult_gene:cardiovascular system(MA)	3/76	3/453	5.960526316	0.04446	0.71141567
adult_gene:endocrine system(MA)	3/76	3/453	5.960526316	0.04446	0.71141567
adult_gene:musculoskeletal system(MA)	3/76	3/453	5.960526316	0.04446	0.71141567
adult_gene:urinary system(MA)	3/76	3/453	5.960526316	0.04446	0.71141567
adult_gene:integumental system(MA)	2/76	2/453	5.960526316	0.10412	1
adult_gene:sensory organ system(MA)	2/76	2/453	5.960526316	0.10412	1
adult_gene:hemolymphoid system(MA)	1/76	1/453	5.960526316	0.26923	1
adult_gene:trunk(MA)	1/76	1/453	5.960526316	0.26923	1
embryo_trait:mortality/aging(MP)	1/76	1/453	5.960526316	0.26923	1
adult_trait:immune system phenotype(MP)	1/76	62/453	0.096137521		
adult_trait:hematopoietic system phenotype(MP)	1/76	69/453	0.086384439		

*corrected $P < 0.05$, **corrected $P < 0.01$, ***corrected $P < 0.001$.

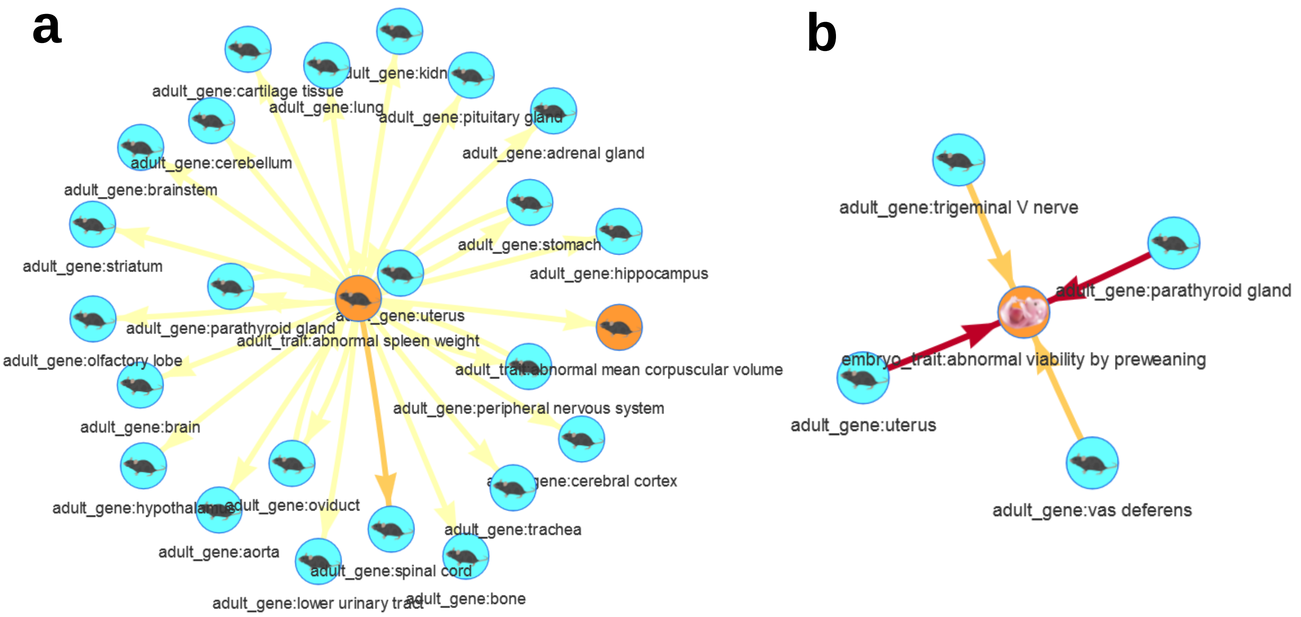

Examples of PPAPs in cluster 1. **(a)** A PPAP with the query phenotype 'adult_trait:abnormal spleen weight'. This query phenotype was related to 24 kinds of abnormal phenotype. Of these 24, 23 belonged to the phenotypic category 'adult/gene' and cover ten kinds of biological systems, corresponding to anatomical locations annotated by MA ontologies. **(b)** A PPAP with the query phenotype 'embryo_trait:abnormal viability by preweaning'. In this case, all four phenotypes related to the query phenotype belonged to the 'stage/type' 'adult/gene'. As edge arrows and edge color gradations represent the direction and strength of rule polarity, respectively, all four phenotypes, particularly 'adult_gene:uterus' and 'adult_gene:parathyroid gland', exhibit a smaller number of abnormal cases than the query phenotype.

Cluster 2

- Cluster 2 consisted of 53 phenotypes (**Supplementary Table 2**), and all 53 phenotypes belonged to the 'stage/type' 'adult/trait' category (56/56 vs. 246/345, two-tailed Fisher's exact test, $P = 0.12$).
- Clusters 2–4 form one large cluster (see **Fig. 6**); hence cluster 2 can be regarded as a sub-cluster of this large cluster.
- See the table below. Enrichment analysis of phenotypes in this cluster, according to their biological systems, showed statistically significant enrichment for phenotypes belonging to the biological systems 'adult_trait:homeostasis/metabolism phenotype(MP)' and 'adult_trait:behavior/neurological phenotype(MP)' (corrected $P = 2.24\times10^{-18}$ and 0.02, respectively). In addition to these two biological system classifications, phenotypes covering 12 biological system classifications were identified in this cluster. Therefore, cluster 2 comprises phenotypes covering 14 biological systems, where phenotypes belonging to the two biological systems ('adult_trait:homeostasis/metabolism phenotype(MP)' and 'adult_trait:behavior/neurological phenotype(MP)') are predominantly responsible for the statistical significance.
- As a representative example of the PPAPs in this cluster, we present a PPAP with the query phenotype 'adult_trait:abnormal circulating HDL cholesterol level', belonging to the biological system 'adult_trait:homeostasis/metabolism phenotype(MP)' (see the figure below).

Enrichment analysis of phenotypes in cluster 2 according to their biological systems

stage_type:top level term	Ratio in cluster 2	Ratio in total	Fold enrichment	*P* value	Bonferroni
adult_trait:adipose tissue phenotype(MP)	1/63	1/453	7.19047619	0.2320486	1
adult_trait:respiratory system(MA)	1/63	1/453	7.19047619	0.2320486	1
adult_trait:homeostasis/metabolism phenotype(MP)	27/63	31/453	6.262672811	2.798E-09	2.238E-08***
adult_trait:behavior/neurological phenotype(MP)	12/63	27/453	3.195767196	0.0029252	0.0234013*
adult_trait:cardiovascular system phenotype(MP)	4/63	12/453	2.396825397	0.1297743	1
adult_trait:integument phenotype(MP)	3/63	11/453	1.961038961	0.247677	1
adult_trait:pigmentation phenotype(MP)	1/63	4/453	1.797619048	0.4821336	1
adult_trait:growth/size/body region phenotype(MP)	2/63	11/453	1.307359307	0.4889224	1
adult_trait:vision/eye phenotype(MP)	3/63	25/453	0.862857143		
adult_trait:craniofacial phenotype(MP)	1/63	11/453	0.653679654		
adult_trait:skeleton phenotype(MP)	2/63	28/453	0.513605442		
adult_trait:limbs/digits/tail phenotype(MP)	1/63	15/453	0.479365079		
adult_trait:hematopoietic system phenotype(MP)	3/63	69/453	0.3126294		
adult_trait:immune system phenotype(MP)	2/63	62/453	0.231950845		

*corrected $P < 0.05$, ***corrected $P < 0.001$.

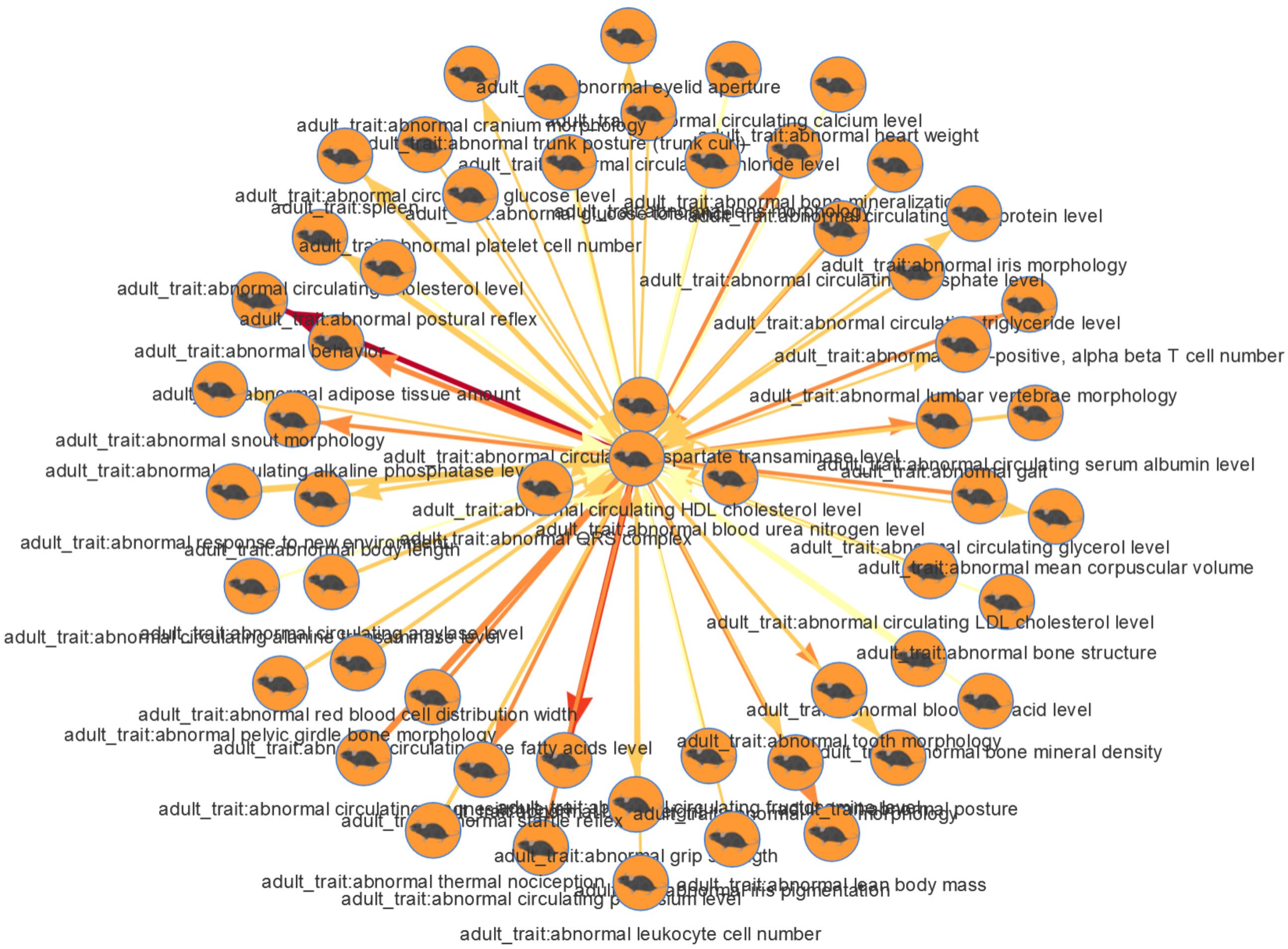

An example PPAP in cluster 2. A PPAP with the query phenotype ‘adult_trait:abnormal circulating HDL cholesterol level’ is shown. This PPAP consists of 56 ‘adult/trait’ category phenotypes covering 13 biological systems.

Cluster 3

- Cluster 3 consisted of 35 phenotypes (**Supplementary Table 2**), all 35 of which belonged to the ‘stage/type’ ‘adult/trait’ (35/35 vs. 246/345, two-tailed Fisher’s exact test, P = 0.2).
- Clusters 2–4 formed one large cluster (see **Fig. 6**); therefore, cluster 3 could be regarded as a sub-cluster of this large cluster.
- See the table below. Cluster 3 consisted of phenotypes covering five biological systems, where phenotypes belonging to the four biological systems ‘adult_trait:skeleton phenotype(MP)’, ‘adult_trait:craniofacial phenotype(MP)’, ‘adult_trait:growth/size/body region phenotype(MP)’, and ‘adult_trait:limbs/digits/tail phenotype(MP)’ were predominantly responsible for the statistical significance (corrected P = 7.9×10^{-10}, 4.1×10^{-4}, 1.7×10^{-3}, and 2.2×10^{-2}, respectively).
- As a representative example of the PPAPs in this cluster, we present a PPAP with the query phenotype ‘adult_trait:abnormal vertebrae morphology’, belonging to the biological system ‘adult_trait:skeleton phenotype(MP)’ (see figure below).

Enrichment analysis of phenotypes in cluster 3 according to their biological systems

stage_type:top level term	Ratio in cluster 3	Ratio in total	Fold enrichment	*P* value	Bonferroni
adult_trait:skeleton phenotype(MP)	25/50	28/453	8.089285714	1.9808E-10	7.92E-10***
adult_trait:craniofacial phenotype(MP)	9/50	11/453	7.412727273	0.00010194	0.00040775***
adult_trait:growth/size/body region phenotype(MP)	8/50	11/453	6.589090909	0.00041252	0.001650084**
adult_trait:limbs/digits/tail phenotype(MP)	7/50	15/453	4.228	0.00572323	0.022892931*
adult_trait:vision/eye phenotype(MP)	1/50	25/453	0.3624		

*corrected $P < 0.05$, **corrected $P < 0.01$, ***corrected $P < 0.001$.

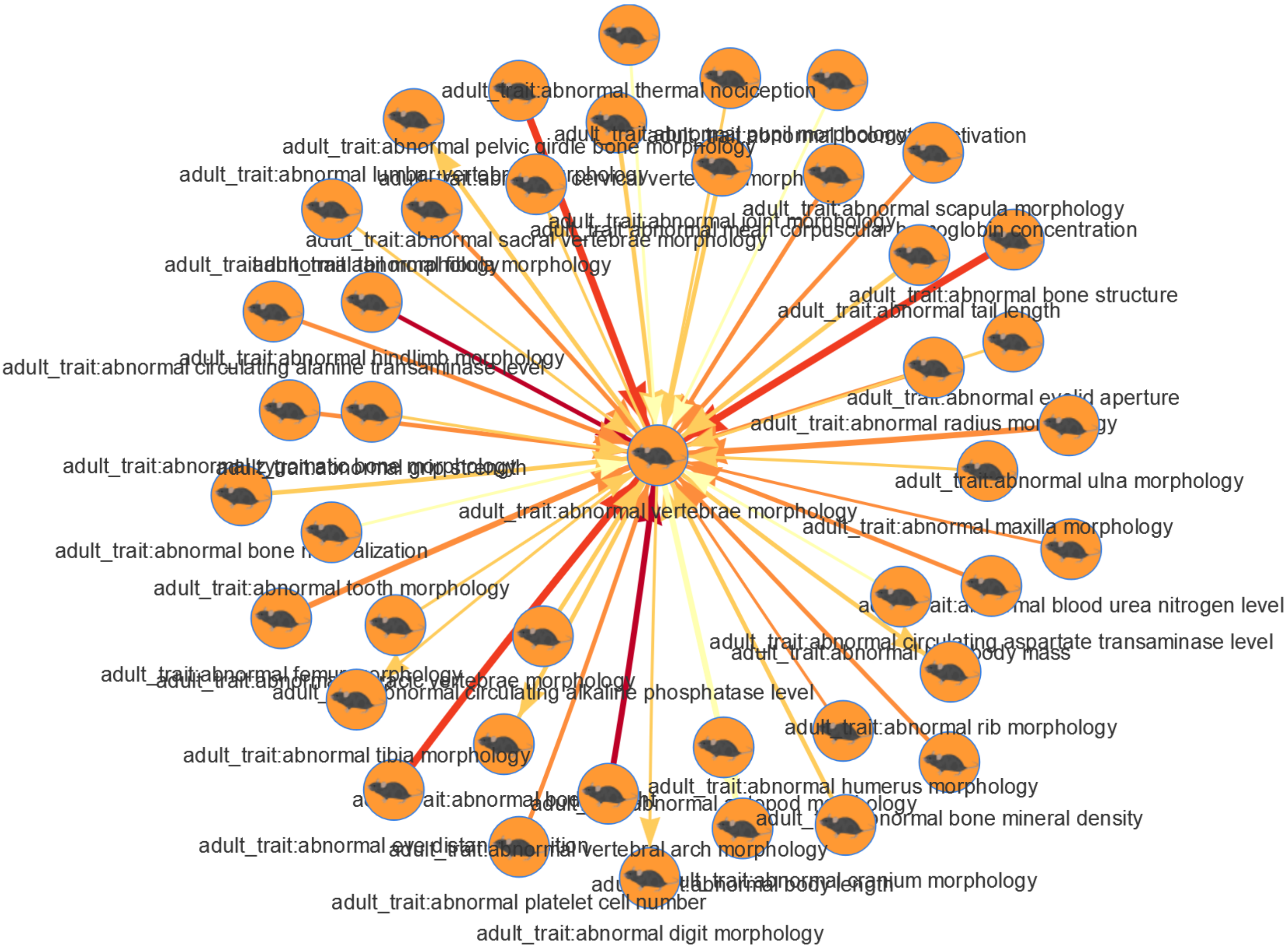

An example of a PPAP in cluster 3. A PPAP with the query phenotype 'adult_trait:abnormal vertebrae morphology' is presented. This PPAP consists of 43 'adult/trait' category phenotypes covering nine biological systems.

Cluster 4

- Cluster 4 consisted of 93 phenotypes (**Supplementary Table 2**), 90 (96.8%) of which belonged to the 'stage/type' 'adult/trait' (90/93 vs. 246/345, two-tailed Fisher's exact test, P = 0.07). The remaining three phenotypes all belonged to the 'embryo/gene' category.
- Eighteen of all nineteen MA ontology-annotated phenotypes derived from gross-/histopathology test parameter measurement were found in this cluster (18/93 vs. 19/345, two-tailed Fisher's exact test, $P = 4.0\times10^{-4}$).

- Clusters 2–4 formed one large cluster (see **Fig. 6**); therefore, cluster 4 could be regarded as a sub-cluster of this large cluster.
- Refer to the table below. Cluster 4 consisted of phenotypes covering 25 biological systems, where only a set of phenotypes belonging to the biological system 'adult_trait:vision/eye phenotype(MP)' predominantly contributed to the statistical significance (corrected $P = 9.2 \times 10^{-3}$).
- As a representative example of a PPAP in this cluster, we present the PPAP with the query phenotype 'adult_trait:abnormal cornea morphology', belonging to the biological system 'adult_trait:vision/eye phenotype(MP)' (see the figure below).

Enrichment analysis of phenotypes in cluster 4 according to their biological systems

stage_type:top level term	Ratio in cluster 4	Ratio in total	Fold enrichment	*P* value	Bonferroni
adult_trait:reproductive system(MA)	5/107	5/453	4.23364486	0.02929792	0.644554
adult_trait:immune system(MA)	3/107	3/453	4.23364486	0.09097229	1
adult_trait:digestive system(MA)	2/107	2/453	4.23364486	0.16997391	1
adult_trait:hearing/vestibular/ear phenotype(MP)	2/107	2/453	4.23364486	0.16997391	1
adult_trait:nervous system phenotype(MP)	2/107	2/453	4.23364486	0.16997391	1
adult_trait:urinary system(MA)	2/107	2/453	4.23364486	0.16997391	1
adult_trait:cardiovascular system(MA)	1/107	1/453	4.23364486	0.34768874	1
adult_trait:endocrine system(MA)	1/107	1/453	4.23364486	0.34768874	1
adult_trait:integumental system(MA)	1/107	1/453	4.23364486	0.34768874	1
adult_trait:nervous system(MA)	1/107	1/453	4.23364486	0.34768874	1
adult_trait:sensory organ system(MA)	1/107	1/453	4.23364486	0.34768874	1
adult_trait:trunk(MA)	1/107	1/453	4.23364486	0.34768874	1
embryo_gene:TS20,alimentary system(EMAP)	1/107	1/453	4.23364486	0.34768874	1
embryo_gene:TS20,integumental system(EMAP)	1/107	1/453	4.23364486	0.34768874	1
embryo_gene:TS20,respiratory system(EMAP)	1/107	1/453	4.23364486	0.34768874	1
adult_trait:vision/eye phenotype(MP)	19/107	25/453	3.217570093	0.00041725	0.00918**
adult_trait:pigmentation phenotype(MP)	3/107	4/453	3.175233645	0.13663165	1
adult_trait:cardiovascular system phenotype(MP)	7/107	12/453	2.469626168	0.05971745	1
adult_trait:integument phenotype(MP)	6/107	11/453	2.309260833	0.09421482	1
adult_trait:reproductive system phenotype(MP)	1/107	2/453	2.11682243	0.47282127	1
adult_trait:limbs/digits/tail phenotype(MP)	7/107	15/453	1.975700935	0.11787784	1
adult_trait:behavior/neurological phenotype(MP)	12/107	27/453	1.881619938	0.06452251	1
adult_trait:hematopoietic system phenotype(MP)	14/107	69/453	0.859000406		
adult_trait:immune system phenotype(MP)	7/107	62/453	0.477992162		
adult_trait:homeostasis/metabolism phenotype(MP)	3/107	31/453	0.409707567		
adult_trait:craniofacial phenotype(MP)	1/107	11/453	0.384876805		
adult_trait:growth/size/body region phenotype(MP)	1/107	11/453	0.384876805		
adult_trait:skeleton phenotype(MP)	1/107	28/453	0.151201602		

**corrected $P < 0.01$.

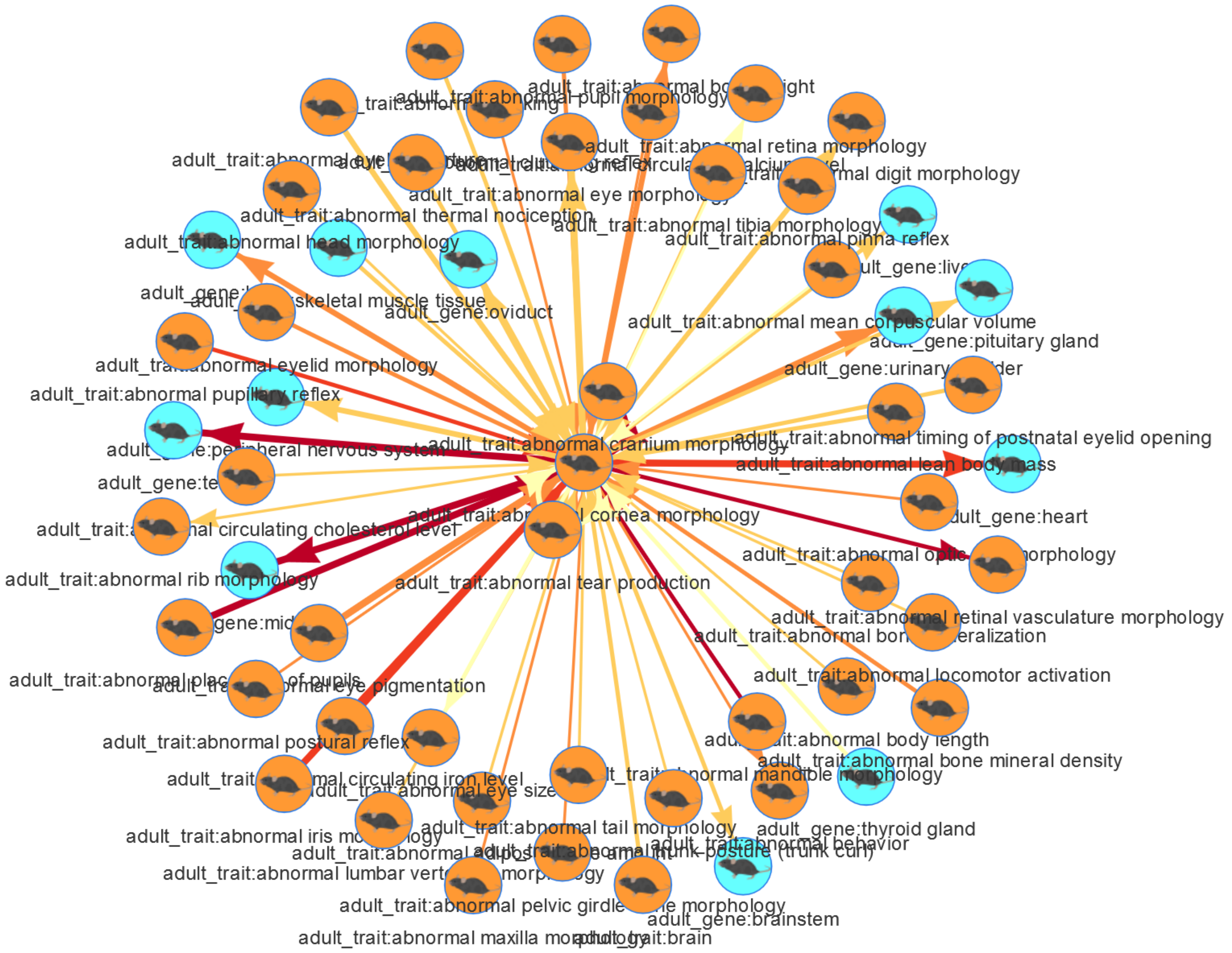

An example PPAP in cluster 4. A PPAP with the query phenotype 'adult_trait:abnormal cornea morphology' is shown. This PPAP consists of 56 phenotypes, comprising 44 'adult/trait' and 12 'adult/gene' category phenotypes, and covers 22 biological systems.

Cluster 5

- Cluster 5 consisted of 29 phenotypes (**Supplementary Table 2**), all 29 of which belonged to the 'stage/type' 'adult/trait' (29/29 vs. 246/345, two-tailed Fisher's exact test, $P = 0.27$).
- Of these 29 phenotypes, 28 belonged to the biological system 'adult_trait:immune system phenotype(MP)'. All 26 phenotypes belonging to the biological system 'adult_trait:hematopoietic system phenotype(MP)' also belonged to the biological system 'adult_trait:immune system phenotype(MP)'.
- Among the 28 phenotypes belonging to the biological system 'adult_trait:immune system phenotype(MP)', many abnormal phenotypes related to T cell immunity, which could affect the acquired immune system, including 'adult_trait:abnormal T cell number' and 'adult_trait:abnormal NK T cell number', were observed.
- Refer to the table below. Cluster 5 consisted of phenotypes covering five biological systems, where phenotypes belonging to the biological systems 'adult_trait:immune system phenotype(MP)' and

‘adult_trait:hematopoietic system phenotype(MP)’ were predominantly responsible for the statistical significance (corrected $P = 1.8 \times 10^{-5}$, 3.3×10^{-4}, respectively).

- As a characteristic example of the PPAPs in this cluster, we present a PPAP with the query phenotype ‘adult_trait:abnormal coping response’, which was the only phenotype belonging to the biological system ‘adult_trait:behavior/neurological phenotype(MP)’ in this cluster (see the figure below).

Enrichment analysis of phenotypes in cluster 5 according to their biological systems

stage_type:top level term	Ratio in cluster 5	Ratio in total	Fold enrichment	*P* value	Bonferroni
adult_trait:digestive/alimentary phenotype(MP)	1/57	1/453	7.947368421	0.2139264	0.8557057
adult_trait:mortality/aging(MP)	1/57	2/453	3.973684211	0.3028002	1
adult_trait:immune system phenotype(MP)	28/57	62/453	3.589134126	4.52E-06	1.808E-05***
adult_trait:hematopoietic system phenotype(MP)	26/57	69/453	2.994660564	8.343E-05	0.0003337***
adult_trait:behavior/neurological phenotype(MP)	1/57	27/453	0.294346979		

***corrected *P* < 0.001.

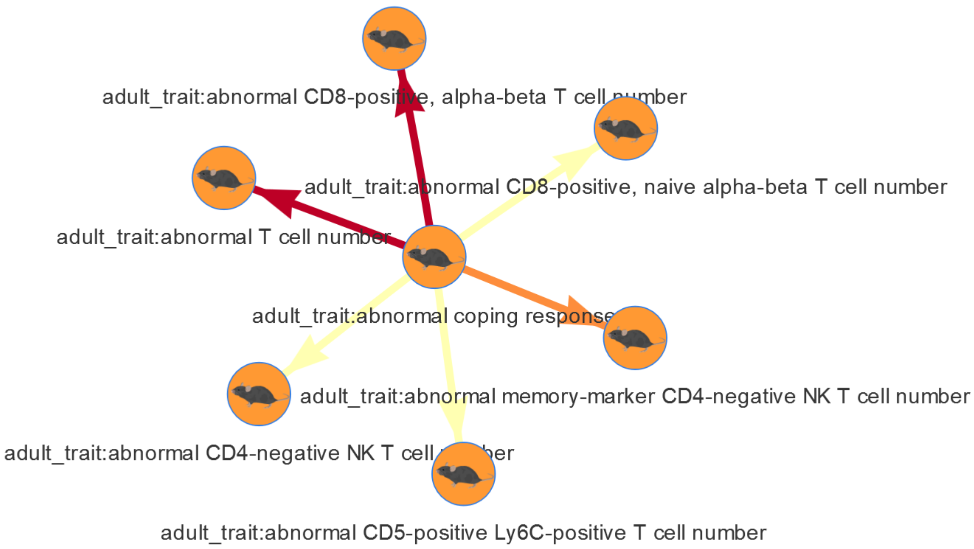

An example PPAP in cluster 5. A PPAP with the query phenotype ‘adult_trait:abnormal coping response’ is shown. All six phenotypes related to the query phenotype belonged to both ‘adult_trait:immune system phenotype(MP)’ and ‘adult_trait:hematopoietic system phenotype(MP)’. These six phenotypes are related to abnormal cell numbers in the T cell lineage. As edge arrows and edge color gradations represent the direction and strength of rule polarity, respectively, all six phenotypes, particularly ‘adult_trait:abnormal T cell number’ and ‘adult_trait:abnormal CD8-positive, alpha-beta T cell number’, exhibit larger numbers of abnormal cases compared with the query phenotype.

Cluster 6

- Cluster 6 consisted of 33 phenotypes (**Supplementary Table 2**). Of the 33 phenotypes, 30 (90.9%) belonged to the ‘stage/type’ ‘embryo/trait’ and were significantly enriched in this cluster (30/33 vs. 35/345, two-tailed Fisher’s exact test, $P = 4.0 \times 10^{-12}$). The remaining three phenotypes (‘adult_trait:abnormal

fertility/fecundity', 'adult_trait:abnormal kidney weight', and 'adult_trait:abnormal circulating unsaturated transferrin level') belonged to the 'adult/trait' category. Note that, as PPAP queries, these three phenotypes had degrees of 5, 1, and 1, respectively, and none were related to 'embryo/trait' phenotypes.

- Refer to the table below. Cluster 6 consisted of phenotypes covering 14 biological systems, where phenotypes belonging to the biological systems 'embryo_trait:embryo phenotype(MP)' and 'embryo_trait:nervous system phenotype(MP)' were predominantly responsible for the statistical significance (corrected $P = 9.2\times10^{-5}$, 1.6×10^{-3}, respectively).
- As a characteristic example of PPAPs in this cluster, we present a PPAP with the query phenotype 'embryo_trait:abnormal eye morphology', with the largest number of constituting phenotypes (see the figure below).

Enrichment analysis of phenotypes in cluster 6 according to their biological systems

stage_type:top level term	Ratio in cluster 6	Ratio in total	Fold enrichment	*P* value	Bonferroni
embryo_trait:embryo phenotype(MP)	9/39	9/453	11.61538462	7.06E-06	9.17643E-05***
embryo_trait:craniofacial phenotype(MP)	3/39	3/453	11.61538462	0.009352	0.121578626
embryo_trait:growth/size/body region phenotype(MP)	3/39	3/453	11.61538462	0.009352	0.121578626
embryo_trait:integument phenotype(MP)	2/39	2/453	11.61538462	0.035981	0.467758007
embryo_trait:vision/eye phenotype(MP)	2/39	2/453	11.61538462	0.035981	0.467758007
adult_trait:renal/urinary system phenotype(MP)	1/39	1/453	11.61538462	0.155538	1
embryo_trait:homeostasis/metabolism phenotype(MP)	1/39	1/453	11.61538462	0.155538	1
embryo_trait:skeleton phenotype(MP)	1/39	1/453	11.61538462	0.155538	1
embryo_trait:nervous system phenotype(MP)	7/39	8/453	10.16346154	0.000119	0.001550187**
embryo_trait:limbs/digits/tail phenotype(MP)	4/39	5/453	9.292307692	0.004339	0.056402944
embryo_trait:cardiovascular system phenotype(MP)	3/39	4/453	8.711538462	0.015322	0.199187436
adult_trait:reproductive system phenotype(MP)	1/39	2/453	5.807692308	0.223777	1
embryo_trait:liver/biliary system phenotype(MP)	1/39	2/453	5.807692308	0.223777	1
adult_trait:homeostasis/metabolism phenotype(MP)	1/39	31/453	0.374689826		

**corrected $P < 0.01$, ***corrected $P < 0.001$.

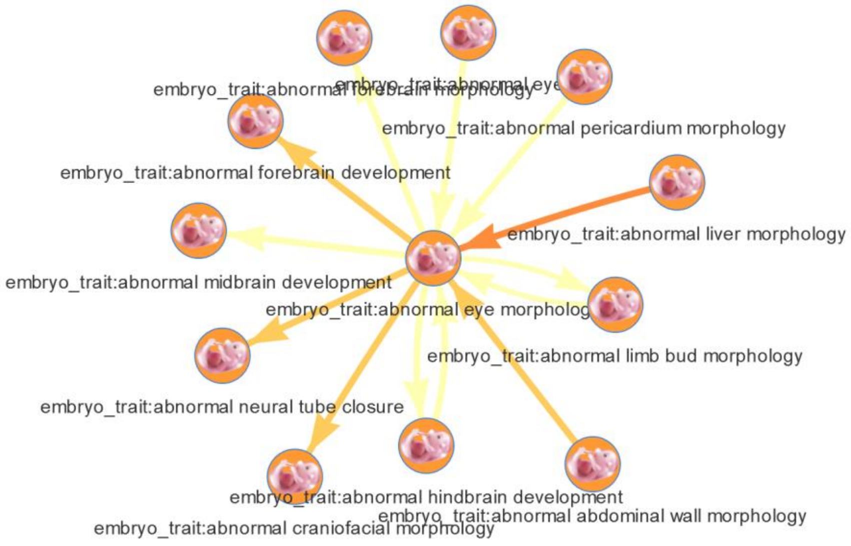

An example PPAP in cluster 6. A PPAP with the query phenotype 'embryo_trait:abnormal eye morphology' is shown. This PPAP comprised 12 'embryo/trait' category phenotypes covering eight biological systems.

Cluster 7

- Cluster 7 consisted of 36 phenotypes (**Supplementary Table 2**). Of the 36 phenotypes, 32 (88.9%) belonged to the 'stage/type' 'adult/trait' (32/36 vs. 246/345, two-tailed Fisher's exact test, P = 0.4). The remaining four phenotypes belonged to the 'embryo/trait' category, and phenotypes in this 'stage/type' category were significantly enriched in the neighboring cluster, cluster 6.
- Refer to the table below. Cluster 7 consisted of phenotypes covering 11 biological systems, where phenotypes belonging to the biological systems 'adult_trait:immune system phenotype(MP)' and 'adult_trait:hematopoietic system phenotype(MP)' were predominantly responsible for the statistical significance (corrected P = 1.5×10^{-3}, 2.5×10^{-3}, respectively). Note that the feature of cluster 7, that phenotypes belonging to each of the above two biological systems were significantly enriched, was the same as that observed in cluster 5.
- Of the 36 phenotypes in this cluster, 24 belonged to the biological systems 'adult_trait:immune system phenotype(MP)' and 'adult_trait:hematopoietic system phenotype(MP)'. Among these 24 phenotypes, those participating in the innate immune system, such as 'adult_trait:abnormal monocyte cell number', 'adult_trait:abnormal dendritic cell number', and 'adult_trait:abnormal neutrophil cell number', and B cell immunity-related phenotypes participating in the acquired immune system, such as 'adult_trait:abnormal B cell number', 'adult_trait:abnormal immature B cell number', and 'adult_trait:abnormal mature B cell number' were particularly prominent.
- As a characteristic example of the PPAPs in this cluster, we present a PPAP with the query phenotype 'adult_trait:abnormal B cell number', which had the largest number of constituting phenotypes (see the figure below).

Enrichment analysis of phenotypes in cluster 7 according to their biological systems

stage_type:top level term	Ratio in cluster 7	Ratio in total	Fold enrichment	*P* value	Bonferroni
adult_trait:mortality/aging(MP)	1/61	2/453	3.713114754	0.318889	1
embryo_trait:liver/biliary system phenotype(MP)	1/61	2/453	3.713114754	0.318889	1
adult_trait:immune system phenotype(MP)	24/61	62/453	2.874669487	0.000217	0.001521**
adult_trait:hematopoietic system phenotype(MP)	25/61	69/453	2.690662865	0.000363	0.002539**
embryo_trait:cardiovascular system phenotype(MP)	1/61	4/453	1.856557377	0.472039	1
embryo_trait:limbs/digits/tail phenotype(MP)	1/61	5/453	1.485245902	0.534988	1
adult_trait:integument phenotype(MP)	2/61	11/453	1.350223547	0.473589	1
embryo_trait:nervous system phenotype(MP)	1/61	8/453	0.928278689		
adult_trait:cardiovascular system phenotype(MP)	1/61	12/453	0.618852459		
adult_trait:vision/eye phenotype(MP)	2/61	25/453	0.594098361		
adult_trait:behavior/neurological phenotype(MP)	2/61	27/453	0.550091075		

**corrected $P < 0.01$.

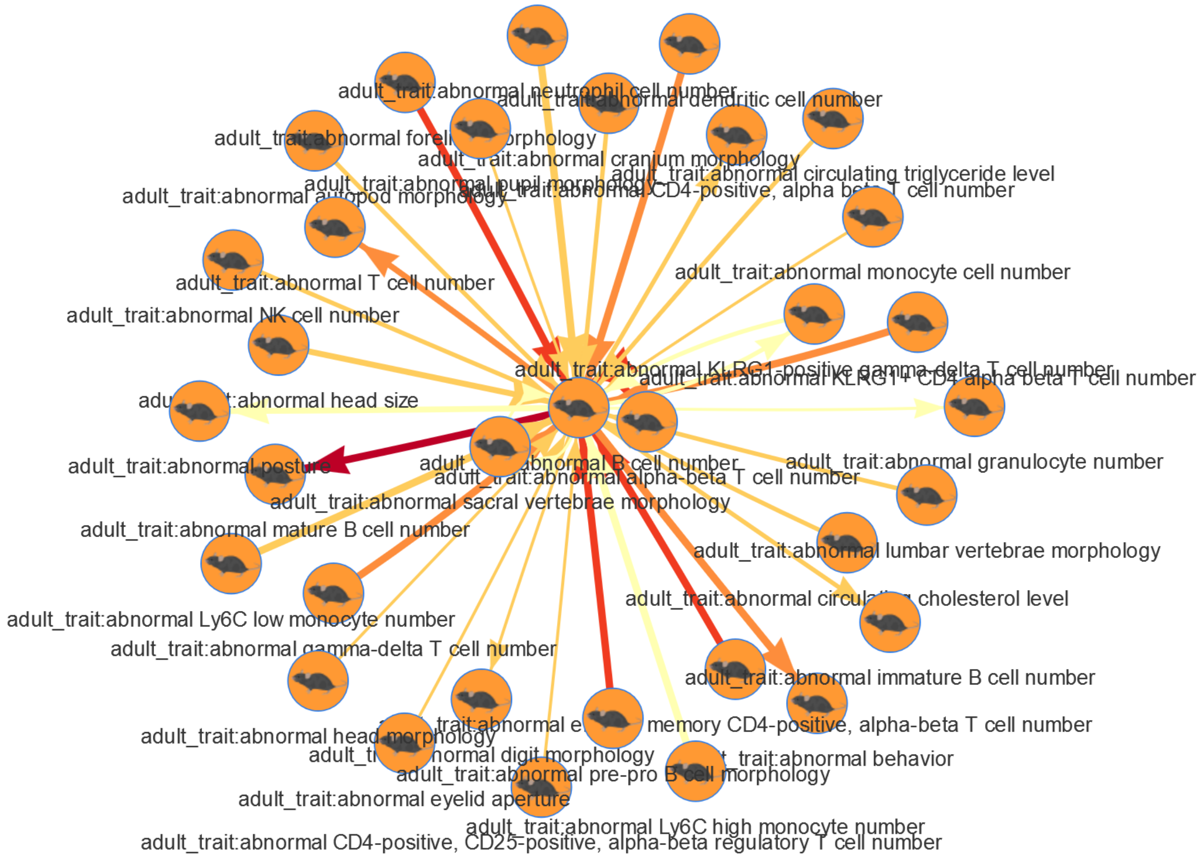

An example PPAP in cluster 7. A PPAP with the query phenotype 'adult_trait:abnormal B cell number' is shown. This PPAP consists of 33 'adult/trait'-categorized phenotypes, covering nine biological systems. Notably, the query phenotype 'adult_trait:abnormal B cell number' is related to numerous phenotypes involving T cells, such as 'adult_trait:abnormal T cell number', which are primarily grouped in cluster 5.

Supplementary Methods

Data preparation and preprocessing for analysis of phenotypic associations

Comprehensive mouse phenotype data and calculation of statistics for phenotypic calls

We obtained a dataset (Release 4.3) from the FTP site (ftp://ftp.ebi.ac.uk/pub/databases/impc/) of the International Mouse Phenotyping Consortium for analysis. To prepare all records (data points) for analysis of phenotypic associations, we first performed data processing using two tables, 'unidimensional_observation' and 'categorical_observation', containing quantitative and qualitative data, respectively. The quantitative dataset was organized into **Supplementary Data 1**, and the qualitative dataset was organized into two tables, **Supplementary Data 2** and **Supplementary Data 3** (from gross pathology and histopathology tests). The dataset consisting of all three tables comprises 18,111,889 records. Using this dataset, basic statistics, *P* values from significance tests, effect sizes (ES, unbiased Hedges' g), and *P* values for the ES (g), were calculated by both colony ID (experimental group) and sex. Note that, for gross pathology and histopathology tests, these values were not calculated according to sex because of small sample sizes. The control strategy (method of selecting control groups) for statistical tests followed output values ('baseline_all' or 'concurrent') from *PhenStat*[1], an R package for standardized analysis of high throughput phenotypic data. *P* values for significance tests were obtained by calculating exact probability; that is, *P* values for quantitative data were acquired by two-tailed permutation test, using the R package *perm*, while *P* values for qualitative data were acquired by two-tailed Fisher's exact test using the R package *exact2x2*. Effect sizes (unbiased Hedges' g) and accompanying *P* values were acquired using the R package *compute.es*. The results of calculations of these statistics are summarized in **Supplementary Data 4** for the quantitative data (**Supplementary Data 1**) and in **Supplementary Data 5** and **Supplementary Data 6** for the two types of qualitative data (**Supplementary Data 2 and 3**).

Assignation of meta-data (gene symbols and ontologies)

A correspondence table for binding meta-data, such as MGI gene and allele IDs, to individual phenotypes was constructed based on the downloaded tables 'biological_model', 'biological_model_allele', 'biological_model_genomic_feature', 'allele', 'allele_acc', and 'genomic_feature' (**Supplementary Data 7**). A correspondence table for binding ontology terms, such as Mammalian Phenotype Ontology (MP)[2], Mouse Anatomy Ontology (MA)[3], and Edinburgh Mouse Atlas Project (EMAP)[4], to measured parameters was also built, based on the downloaded tables 'phenotype_parameter_ontology_annotation' and 'phenotype_parameter_lnk_ontology_annotation'. Further, to enable discrimination by both stage (adult/embryo) and type (trait/gene) of phenotypic expression, we added a column ('stage_type:ontology_name') with phenotype names, in the form of 'stage_type:ontology_name' (e.g., 'adult_trait:abnormal adipose tissue amount'), to the correspondence table. Consequently, a 'parameters-phenotypes' table, in which the 2,254 measured parameters correspond to 512 phenotypes, in the form 'stage_type:ontology name', was created (**Supplementary Data 8**). After adding meta-data from the two tables (**Supplementary Data 7 and 8**) to the three statistical summary tables (**Supplementary Data 4–6**), the three tables containing meta-data were summarized into a single table.

Supplementary Data 9 was then built by deleting records where the number of individuals in the control group was ≤ 1 or the number of individuals in the mutant group was 0 from this table.

Creating a table of phenotypic calls for fertility and viability

Phenotypic calls for two kinds of tests, fertility and viability, linked to each experiment group (colony_id), not each individual, are summarized in **Supplementary Data 10** and **Supplementary Data 11**, respectively. The results of manual calls of phenotypes (normal or abnormal) for each experiment group (colony_id) are presented in the column 'call_fertility_fecundity' in **Supplementary Data 10** and in the column 'call_viability_by_preweaning' in **Supplementary Data 11**. These two types of data were used for subsequent association rule mining as the two phenotypes 'adult_trait:abnormal fertility/fecundity' and 'embryo_trait:abnormal viability by preweaning', respectively.

Integration of semantically identical phenotypes

To integrate semantically identical phenotyping results, we reshaped the data in **Supplementary Data 9**, according to genes (gene symbols), based on the value (phenotype) in the column 'stage_type_ontology name'. Specifically, to make the reshaped dataset (**Supplementary Data 12**), when multiple measured parameters were identified in the same phenotype (from the column 'stage_type_ontology_name') in each mutant gene categorization (from the column 'gene_symbol'), the one with the highest degree of abnormality was selected as representative of the phenotype, in priority order of the one with the smallest *P* value for ES (unbiased Hedge's g), the one with the smallest *P* value for the significance test, and the one with the largest ES.

A dataset for association rule mining

A dataset obtained by adding phenotypic calls from **Supplementary Data 10** (Fertility) and **Supplementary Data 11** (Viability) into the table, **Supplementary Data 12**, was used to construct a normal/abnormal phenotypic call matrix of 532 phenotypes × 3,100 gene symbols (mutant strains) for subsequent association rule mining. The 532 phenotypes were derived from 2,050 measured parameters, and cover 84 distinct biological systems (top level terms), classified into four 'stage/type' phenotype expression categories: 35 in 'adult/trait', 16 in 'adult/gene', 16 in 'embryo/trait', and 17 in 'embryo/gene' (**Supplementary Table 1**).

Data availability

Supplementary Data 1–12 mentioned above are available at the following URL:
https://doi.org/10.6084/m9.figshare.7995911/

References

1. Kurbatova, N., Mason, J. C., Morgan, H., Meehan, T. F. & Karp, N. A. PhenStat a tool kit for standardized analysis of high throughput phenotypic data. *PLoS One* **10**, 1–16 (2015).
2. Smith, C. L., Goldsmith, C. W. & Eppig, J. T. The Mammalian Phenotype Ontology as a tool for annotating, analyzing and comparing phenotypic information. *Genome Biol.* **6**, R7 (2005).
3. Hayamizu, T. F., Baldock, R. A. & Ringwald, M. Mouse anatomy ontologies : enhancements and tools for exploring and integrating biomedical data. *Mamm. Genome* **26**, 422–430 (2015).
4. Hayamizu, T. F. *et al.* Open Access EMAP / EMAPA ontology of mouse developmental anatomy : 2013 update. *J. Biomed. Semantics* **4**, 1–5 (2013).
